# Characterization of Nanoparticle Batch-To-Batch Variability

**DOI:** 10.3390/nano8050311

**Published:** 2018-05-08

**Authors:** Sonja Mülhopt, Silvia Diabaté, Marco Dilger, Christel Adelhelm, Christopher Anderlohr, Thomas Bergfeldt, Johan Gómez de la Torre, Yunhong Jiang, Eugenia Valsami-Jones, Dominique Langevin, Iseult Lynch, Eugene Mahon, Inge Nelissen, Jordi Piella, Victor Puntes, Sikha Ray, Reinhard Schneider, Terry Wilkins, Carsten Weiss, Hanns-Rudolf Paur

**Affiliations:** 1 Institute for Technical Chemistry (ITC), Karlsruhe Institute of Technology (KIT), 76131 Karlsruhe, Germany; hanns-rudolf.paur@kit.edu; 2 Institute for Toxicology and Genetics (ITG), Karlsruhe Institute of Technology (KIT), 76131 Karlsruhe, Germany; silvia.diabate@kit.edu (S.D.); marco.dilger@partner.kit.edu (M.D.); carsten.weiss@kit.edu (C.W.); 3 Institute for Applied Materials (IAM), Karlsruhe Institute of Technology (KIT), 76131 Karlsruhe, Germany; edelhalm@t-online.de (C.A.); Thomas.bergfeldt@kit.edu (T.B.); 4 Institute for Technical Thermodynamics and Refrigeration (ITTK), Karlsruhe Institute of Technology (KIT), 76131 Karlsruhe, Germany; Christopher.anderlohr@kit.edu; 5 Department of Engineering Sciences, Applied Materials Science, Uppsala University, 752 36 Uppsala, Sweden; johan.gomezdelatorre@gmail.com; 6 Department of Architecture and Civil Engineering, Claverton Down, University of Bath, Bath BA2 7AY, UK; y.jiang@bath.ac.uk; 7 School of Geography, Earth and Environmental Sciences, University of Birmingham, Birmingham B15 2TT, UK; e.valsamijones@bham.ac.uk (E.V.-J.); i.lynch@bham.ac.uk (I.L.); 8 Laboratoire de Physique des Solides, CNRS UMR 8502, Université Paris Sud 11, Université Paris Saclay, 91190 Saint-Aubin, France; dominique.langevin@u-psud.fr; 9 Centre for BioNano Interactions, School of Chemistry and Chemical Biology, University College Dublin, Dublin 4, Ireland; eugene.mahon@ucd.ie; 10 Health Department, Flemish Institute for Technological Research (VITO), 2400 Mol, Belgium; inge.nelissen@vito.be; 11 Catalan Institute of Nanoscience and Nanotechnology (ICN2), CSIC and The Barcelona Institute of Science and Technology, 08036 Barcelona, Spain; jordi.piella@icn.cat (J.P.); victor.puntes@icn.cat (V.P.); 12 Science and Technology of Nanosystems (STN), Karlsruhe Institute of Technology (KIT), 76131 Karlsruhe, Germany; Sikha.ray@kit.edu; 13 Laboratory for Electron Microscopy (LEM), Karlsruhe Institute of Technology (KIT), 76131 Karlsruhe, Germany; Reinhard.schneider@kit.edu; 14 Faculty of Engineering, School of Chemical and Process Engineering, University of Leeds, Leeds LS2 9JT, UK; T.A.Wilkins@leeds.ac.uk

**Keywords:** nanosafety, particle size, impurities, reactive oxygen species

## Abstract

A central challenge for the safe design of nanomaterials (NMs) is the inherent variability of NM properties, both as produced and as they interact with and evolve in, their surroundings. This has led to uncertainty in the literature regarding whether the biological and toxicological effects reported for NMs are related to specific NM properties themselves, or rather to the presence of impurities or physical effects such as agglomeration of particles. Thus, there is a strong need for systematic evaluation of the synthesis and processing parameters that lead to potential variability of different NM batches and the reproducible production of commonly utilized NMs. The work described here represents over three years of effort across 14 European laboratories to assess the reproducibility of nanoparticle properties produced by the same and modified synthesis routes for four of the OECD priority NMs (silica dioxide, zinc oxide, cerium dioxide and titanium dioxide) as well as amine-modified polystyrene NMs, which are frequently employed as positive controls for nanotoxicity studies. For 46 different batches of the selected NMs, all physicochemical descriptors as prioritized by the OECD have been fully characterized. The study represents the most complete assessment of NMs batch-to-batch variability performed to date and provides numerous important insights into the potential sources of variability of NMs and how these might be reduced.

## 1. Introduction

Establishment of standards in nanomaterial (NM) synthesis and processing to ensure reproducibility of biological responses is an important task with respect to the validity of scientific conclusions and the enforcement of legislation on NMs. Current industrial and laboratory processes for synthesis of NMs give rise to quite significant variability from batch-to-batch (B2B) [1,2,3]. Such variations are quite well known and considerable efforts are being made to reduce them for high added value applications. However, while such variability may not impact on the industrial applications of NMs (such as catalysis, material strengthening, energy conversion etc.) it may have significant implications for the evaluation of biological impacts of the NMs needed as part of responsible innovation, risk assessment and regulation. Thus, different detailed biological outcomes from different batches (even if none implies any real hazard) introduce a lack of certainty in the science [4,5]. To some degree the (immediate) need for standard test materials (for example the OECD sponsorship program materials) can be addressed by purchase of a large single batch. However, it is clear that in the long term this will not work, as the shelf life of some NM dispersions may be as short as 3 months and full in vivo programs can take 2 years. There is also the question of how ‘representative’ a single batch is of the NM properties across multiple batches. For these reasons, it is necessary to gain control over the reproducibility of batches (or have sufficiently ‘representative’ batches in sufficient quantities) in order to really progress knowledge on the safety of NMs and thus remove uncertainty in the field. Thus, research is needed in order to identify the source of B2B variations within the synthesis processes [6] and to develop strategies to eliminate or reduce them, including evaluation of currently available methods (for example continuous flow) to address the problem. Understanding of the correlation between synthesis route and resulting physicochemical characteristics and toxicity, such as has been shown for colloidal versus flame synthesized silica [7] would also allow disentangling some of the apparently contradictory results present in current literature, where nominally similar materials are reported to have very different toxicities. Representative materials produced by different methods could then be utilized as positive and negative controls respectively, once their properties and toxicities are known [8].

For silica NMs, particle properties such as size, morphology, porosity, crystallinity [9], colloidal stability [3] and surface chemistry [10] are known factors in determining biological impact [4]. Indeed, synthesis route is well known to affect the properties, including toxicity, of resulting NMs, as has been shown for silica [7], ceria [6], silver [11] and many other NMs. Therefore, in order to study and hence design “safer” NM systems for practical applications such as cosmetics [12], silicon wafer polishing, electronics and so forth, high level of control and robust reproducible preparation methods are required. Additionally, in order to correctly study and interpret the biological impact of NMs, it is vital to reach a state where exquisite control can be applied to NM synthesis in order to achieve the level of reproducibility required.

For NMs that are applied as viable biospecific carrier systems for therapeutic delivery, B2B reproducibility is also a prerequisite. Given the surface sensitivity of the dynamic biological environment, small variations at even the sub-nano level may lead to unpredictable outcomes. Consequently, only with a sufficient level of synthetic control we may achieve any useful predefined and designed-in biological impact. For example, three different particles of the same bulk material (ceria) revealed different protein adsorption patterns in alveolar lining fluid, despite having similar surface area and surface chemistry [13].

The Organization for Economic Co-operation and Development (OECD) set up a working party on manufactured nanomaterials (WPMN) to define and update critical issues concerning the analysis of physico-chemical properties [14,15]. Available techniques were compared and validated for a distinct and limited set of NMs. Three major categories were suggested:

- Characterisation (“what they are”)—physical and chemical identification in terms of composition, impurities, size and size distribution, shape, surface characteristics and so forth;
- Fate (“where they go”): biological (toxicokinetics, biodistribution) and environmental fate described by solubility, hydrophobicity, dispersibility, dustiness and so forth and;
- (Re)activity (“what they do”): their reactivity, physical hazards, biological reactivity, toxicodynamics, photoreactivity and so forth

Whereas the focus of these studies was primarily on the identification and validation of suitable methods identifying potential pitfalls and pro and cons dependent on the method and NM under investigation, only a restricted number of NMs were investigated.

Hence, the aim of this work was to extend this work by analysing more NMs produced by the same synthesis route (B2B variability) or different routes and assess the impact and reproducibility of synthesis procedures on physico-chemical properties. We looked at four commonly employed NM types from the OECD priority particle list [16], namely SiO_2_, TiO_2_, CeO_2_ and ZnO. Amine-modified polystyrene was also assessed, as this material is often used as a positive control in nanotoxicity experiments [17]. Aqueous synthesis and flame synthesis routes were employed and evaluated for B2B variability and sources of variability that potentially have biological implications are discussed. Nanomaterial synthesis processes were maintained in highly controlled reactors with specific parameters (e.g., mixing conditions, temperature etc.) and were varied systematically in each case to look at batch-to-batch variation under a range of conditions. Physiochemical properties of the resultant batches including most of the relevant OECD proposed parameters [14,18] complemented with surface structural and compositional analysis were measured by a set of techniques as detailed in Table 1 in an effort to characterize both dry and dispersed NM samples to the highest degree using state of the art approaches. Based on these data, physicochemical properties for the most widely used NMs are discussed which are most vulnerable to B2B variability. Likely potential sources of B2B variability and strategies to mitigate them, as well as the impact of B2B variability for NM safety assessment are discussed. In concordance with previous findings mainly by the OECD WPMN, we confirm the suitability of the proposed techniques and methods including potential shortcomings for a wider range of NMs produced by multiple synthesis routes. Moreover, we used the DCF assay as an additional approach to detect radical formation as a proxy for reactivity. Finally, particle size measurements of especially agglomerated NMs by mobility spectrometry (SMPS) provide more reliable data than by dynamic light scattering (DLS).

## 2. Materials and Methods

To establish many of the analytical methods in an early stage two silica NMs were selected: Representing the commercial materials the flame synthesized Aerosil200^®^ (Evonik, Germany) named SilNP_001 was used and representing the Stöber synthesized silica, SilNP_002 was produced as described in the following chapter.

### 2.1. Production Methods for Silica NMs

#### 2.1.1. Liquid Suspended Silica NMs Using Stöber Synthesis

**Figure 1:**
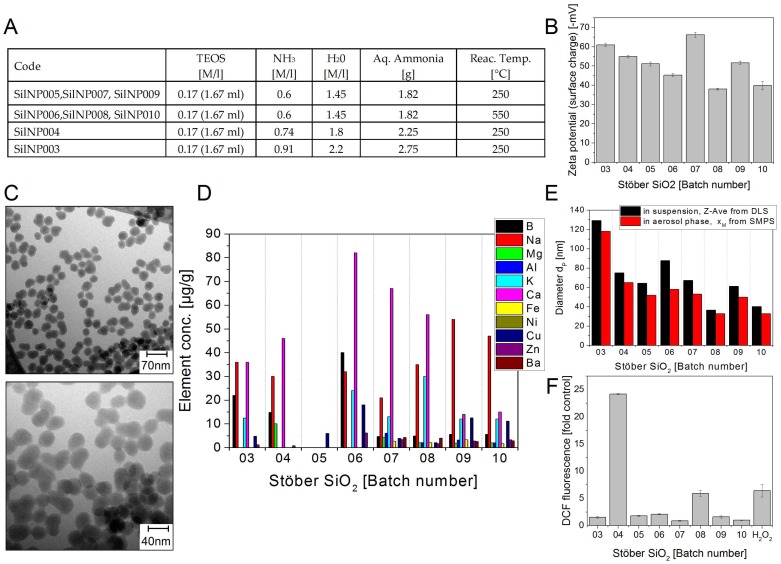
Stöber synthesized silica SilNP03–10 (**A**) Production parameters for the different Stöber synthesized silica nanoparticles; (**B**) Zeta potential representing the surface charge in –mV at pH 8.4 ± 0.57; (**C**) Representative transmission electron microscopy (TEM) images (**D**) Selected impurities present in the different Stöber synthesized SiO_2_ NM batches. The mass concentrations of elements above detection limit are shown. The data represent the mean of two assays; (**E**) Particle diameters d_P_ in nm: Z-Average of d_P_ determined in aqueous suspension using dynamic light scattering (DLS) (black bars) and modal value x_M_ of the number size distribution in the aerosol phase measured by mobility spectrometry (SMPS) (red bars); (**F**) Potential to induce ROS. The test NMs were delivered as suspensions of 5 mg/mL in water. The graph shows the Dichlorofluorescein (DCF) fluorescence induced by the different batches at 400 µg/mL relative to a sample without NMs (control). 0.3 µM H_2_O_2_ was used as a positive control. Additional tested was the commercial material SilNP02. The data represent mean values of two independent experiments with three replicates ± s.e.m.

Aqueous silica NM dispersions were prepared by a typical Stöber type reaction [19] followed by purification into water. This process involves the hydrolysis and condensation of a silicate precursor (tetraethylorthosilicate (TEOS)) in alcohol solutions catalysed by ammonia. There are a number of approaches for controlling the final dispersion properties for such NMs including the TEOS concentration, alcohol type, water content and temperature [20,21]. In this work, we varied the concentration of aqueous ammonia present in the system and also the temperature to modulate the final NM diameter (Figure 1A).

To the required amount (Figure 1A) of aqueous ammonia (28%, Sigma, cat. No. 320145, used as received) in a polypropylene container ethanol (99.9%, Sigma, cat. No. 320145, used as received) was added to a volume of 50 mL. The solution was then stirred at 25 °C, 600 rpm on a plate stirrer. To this rapidly stirring solution TEOS (1.76 g, 99.9%, Sigma, cat. No. 86578, used as received) was added in one aliquot. The solution was then sealed under a blanket of N_2_ and stirred for a further 20 h at 25 °C.

For particle cleaning the resulting NM suspension was divided into fractions and centrifuged down at 14,000 rpm for 20 min and the pellet re-suspended in fresh ethanol aided by bath sonication. This washing procedure was repeated twice with ethanol followed by three water washes and a final re-suspension in pure water to a final concentration of 10 mg/mL NMs.

For NMs smaller than 40 nm in diameter, benchtop centrifugation could not be used to purify the dispersion. For these cases evaporation was employed. Following synthesis, the dispersions were transferred to a rotary evaporator where the ammonia was evaporated at 40 °C. Twenty mL of pure water was then added to the dispersion followed by evaporation of ethanol leaving half the initial volume. A further 20 mL of deionized water was added and the remainder of the ethanol was evaporated to leave an aqueous dispersion.

#### 2.1.2. Dry Bulk Silica NMs from Flame Synthesis

Flame synthesis is one of the most widely used industrial processes to produce NMs. NMs produced at high volume (soot, metal oxides) are frequently manufactured from gaseous precursors by flame synthesis, which provides high purity but invariably yields agglomerates and aggregates of NMs [22]. The degree of agglomeration, however, has a large influence on the possible applications of the particles. The lack of structural and compositional control makes these processes unsuitable for high quality applications, yet these products are widely used as fillers for polymers (polymers, paints) to improve their mechanical and optical properties. Recent laboratory studies attempting to overcome the agglomeration have reduced the flame temperature and applied staged pressure reactors.

To produce silica NMs, gaseous TEOS was injected into a methane flame via a nitrogen carrier gas stream. The precursor (TEOS) was metered by a syringe pump and sprayed utilizing the nitrogen through a 2-phase nozzle into a heated packed column. There it was evaporated into the gas stream. In this production the mass flow of the precursor, temperature and the stoichiometry of the flame were varied.

Downstream of the flame reactor the hot flue gas was diluted by injection of air resulting in a temperature decrease to around 200 °C. The particles were filtered from the gas by polytetrafluoroethylene membrane bag filters (Type 8328, Gore^®^). The NM concentration, as monitored by a condensation particle counter downstream of the fabric filter, was below the detection limit. After the end of the experiment, the filter housing was removed from the setup and opened. By rapping and careful scraping with a metal spoon, the NMs were removed from the filter bags. The recovered NMs were stored in plastic or glass bottles and homogenized with the spoon. Afterwards the containers were sealed and the yield was determined by the weight difference compared to the empty containers. The bag filters were thoroughly cleaned with clean pressurized air and reused for the next production run. Altogether, NMs were produced at two different experimental conditions. Three batches were prepared at each condition, as outlined in Tables S1–S2.

During the production of the first two batches the clean gas leaving the filter unit was drawn off by a fluid ring pump. To compensate for the rising pressure drop over the filter, the vacuum was periodically increased by a valve resulting in a saw tooth like pressure-time-profile. For all other batches, no pump was used, resulting in a constantly increasing pressure drop in the filter unit.

Aerosil^®^200 (Evonik, Frankfurt, Germany) was added to this group (batch name SilNP_001) to optimize the characterization methods.

### 2.2. Zinc Oxide NMs

Polyol mediated synthesis has turned out to be well suited for the preparation of ZnO particles in the size range of 30–100 nm [23,24]. In the present investigation, ZnO NMs were prepared by polyol mediated precipitation. This process involved dissolving zinc acetate as a metal precursor in diethylene glycol (DEG), adding a small amount of water. This mixture is rapidly heated to 180 °C. The precipitation of oxide occurred at a specific temperature. The surface of the growing NMs is immediately complexed by DEG as a chelating agent. As a result of this chelation, grain growth is limited. There are a number of approaches for controlling the final NMs properties, such as metal precursor concentration, water content and temperature. In this work, we varied the concentration of zinc acetate present in the system.

Fifty mL of DEG (Sigma Aldrich, Taufkirchen, Germany, 99% Reagent Plus) in a 100 mL round bottom flask was heated to 50 °C by a temperature controlled oil bath. The required amount of zinc acetate (Sigma Aldrich, Taufkirchen, Germany, 99% ACS Reagent Grade) and 1 mL of MilliQ^®^ triple distilled water was added into the polypropylene container (Tables S1–S3). The container was heated under reflux with an air condenser to 140 °C as quickly as possible and left at this temperature for 1 h until the zinc acetate was dissolved. Then the temperature was increased to 180 °C as quickly as possible and kept at this temperature for 2 h. Finally, the solution was cooled and placed in the furnace for 3 h at 350 °C.

### 2.3. Titanium Dioxide NMs

Titanium dioxide NMs were synthesized following modified published procedures [25] (Tables S1–S4). In a typical preparation, a stock solution of Ti^4+^ ion concentration of 0.7 M was prepared by dilution of TiCl_4_ in HCl (3 M) at room temperature. After 5 min under stirring all the TiCl_4_ was completely dissolved. To 12 mL of the stock solution, 72 mL of Milli-Q^®^ water was added and the pH adjusted to a selected value by the addition of NaOH (3 M). The precipitation of titanium hydroxides occurred instantaneously at a pH above 1 and was complete. The suspension was diluted to 120 mL to obtain a Ti^4+^ concentration of 70 mM and aged in an oven at a fixed temperature without stirring for 24 h. The solid was collected by centrifugation and washed twice with Milli-Q^®^ water before being suspended in an aqueous solution of tetramethylammonium hydroxide (TMAOH) (5 mM). The suspension was kept under stirring for 48 h. During that time NMs stabilized due to a combined effect of TMAOH and pH. Finally, the samples were diluted with Milli-Q^®^ water to a concentration of 12 mM (1 mg/mL) and TMAOH 1 mM. At these conditions NMs were stable for months. To remove large aggregates, the suspensions were finally centrifuged at low speed.

For comparison to a common used industrial product, the titanium Aeroxide^®^P25 (Evonik, Frankfurt, Germany) was included in the DCF assays.

### 2.4. Amine-Modified (Cationic) Polystyrene NMs

Amine-modified (cationic) polystyrene NMs (PS-NH_2_) were formed by a surfactant free co-polymerization of styrene and *N*-(2-aminoethyl) methacrylamide hydrochloride. The water-soluble amino monomer acts both as stabilizer and surface functionalizing agent for this synthesis (Tables S1–S5).

The required amounts of styrene and divinylbenzene (DVB) were initially combined and washed 3 times with an equal volume of 10% NaOH solution, followed by passage through a short column of activated basic aluminium oxide. 40 mL 2-(*N*-morpholino)-ethanesulfonic acid (MES) buffer solution (pH 5, 20 mM) was brought to reaction temperature and degassed by bubbling nitrogen through for 30 min. Under stirring at 600 rpm *N*-(2-aminoethyl)-methacrylamide hydrochloride was then added followed 1 min later by the styrene and DVB mixture. Azobisisobutyronitrile dissolved in 1 mL of water was injected into the solution which was then sealed and allowed to react for a further 24 h.

The NM dispersions (20 mL) were purified by dialysis against pure water (1 L) in cellulose membranes with a molecular weight cut-off (MWCO) of 3500 Daltons with 4 water changes over two days.

### 2.5. Cerium Dioxide NMs

Cerium dioxide NMs were synthetized following modified published procedures [26,27]. In a typical preparation, equal volumes of Ce^3+^ ion concentration (50 mL Ce(NO_3_)_3_·H_2_O 0.0375 M) and 50 mL hexamethylenetetramine (HMT) (0.5 M) were mixed at room temperature and the obtained solution was aged at 25 °C under stirring for different durations. NMs were formed by oxidation of Ce^3+^ to Ce^4+^ and the subsequent formation of the insoluble species CeO_2_. By controlling the reaction time, batches of NMs with different sizes were obtained (Tables S1–S6). NMs were collected by centrifugation (15,000 rcf for 30 min) and re-suspended in MilliQ^®^ water with the use of ultra-sonication. The final concentration of cerium dioxide NMs was 3.2 mg/mL. At these conditions NMs slowly sediment after some days but they could be easily re-dispersed. For NMs synthetized with TMAOH the same procedure was followed but a stock solution of TMAOH (0.04 M) was used instead of HMT (0.5 M).

### 2.6. Physicochemical Characterisation of NM Batches

Numerous methods were used to fully characterize the different NM batches (Table 1). For comparability and reproducibility of data, standard operation procedures (SOPs) were defined for all methods, which are available in the Supplementary Materials (Supp1_SOPs for characterization).

#### 2.6.1. Representative TEM Pictures and Derived Data

For all investigated NMs batches of the different materials (SiO_2_, ZnO, TiO_2_, PS-NH_2_ and CeO_2_), TEM samples of the NMs were prepared by nebulizing a thereof produced dispersion onto a Cu grid (400 mesh) covered with a combined holey and ultrathin (about 3 nm) carbon film. This was done by means of an ultrasonic apparatus. Using the ultrathin C film helps obtaining a larger area-deposition density in comparison to simple holey carbon films. Furthermore, the 3 nm thick supporting carbon does not drastically disturb the TEM inspection of the NMs because of low additional electron scattering.

Crystallite size and the primary particle size distribution were observed. Typical TEM findings are briefly summarized and compared to the results from characterization according to other methods. Particular emphasis is put on determining, or in the case of apparent problems due to agglomeration/aggregation, at least estimating, average NM sizes.

#### 2.6.2. Particle Size Distribution in Aerosol (SMPS)

The airborne particle size was determined by aerosolisation of NMs using an electrospray aerosol generator (Electrospray Aerosol Generator Model 3480, TSI) and measuring the number size distribution by a scanning mobility particle sizer (SMPS, DMA 3071 with CPC 3775, TSI). Most NMs were able to be aerosolized using the electrospray aerosol generator having an upper size limit of 100 nm but especially the ZnO NMs were too agglomerated to become aerosolized for SMPS measurements. Aerosols typically show a log-normal distribution of size. The number size distribution was measured at least three times. The data were corrected regarding sampling losses using the penetration factor for tubing according to Soderholm [34] and analysed by determining the mean ± standard deviation of the three number measurements in each size channel. The resulting particle number size distribution dN/dlog(d_P_) [1/cm³] can be characterized by the three parameters of the log-normal fits: the modal value x_M_ [nm], the geometric standard deviation σ_geo_ [−/−] and the total number concentration c_N_ [1/cm³]. These three parameters are reported in the data sheets.

#### 2.6.3. Radical Formation Potential (DCF Test)

The radical formation potential of the NMs has been determined by their ability to oxidize the non-fluorescent 2′,7′-dichlorodihydrofluorescein (DCFH_2_) to the fluorescent 2′,7′-dichlorofluorescein (DCF) according to Cathcart et al. [33], Foucaud et al. [32] and Diabaté et al. [35]. In a first step the commercial 2′,7′-dichlorodihydrofluorescein-diacetate (DCFH_2_-DA, Invitrogen, Karlsruhe, Germany) was deacetylated with NaOH. 0.1 mL of 5 mM DCFH_2_-DA in ethanol has been mixed with 2.4 mL of 0.01 N NaOH and incubated at room temperature (24 °C) for 30 min. For neutralization, 10 mL phosphate buffered saline without Ca^2+^ and Mg^2+^ (PBS) has been added and kept on ice in the dark until use. Just prior to use, horseradish peroxidase (HRP, Sigma, Taufkirchen, Germany) has been added as a catalyst (2.2 U/mL). The DCFH_2_ concentration in the working solution was 40 µM. Suspensions of test particles have been sonified for 10 min and different concentrations were prepared in PBS. H_2_O_2_ standard preparations (0.04 μM to 10 μM) were prepared as well. The test solutions have been mixed 1:1 (*v*/*v*) with the prepared DCFH_2_ working solution and incubated at 37 °C for 15 min in the dark. The solutions were then centrifuged (20,000 × *g* for 15 min) to remove the particles. Solutions containing polystyrene nanoparticles were centrifuged for 1 h. The fluorescence of the supernatant was monitored at 485 nm excitation and 530 nm emission using a fluorescence microplate reader (BIO-TEK FL600 from MWG-Biotech AG, Ebersberg, Germany). Results of fluorescence intensity were expressed as fold changes of the particle free sample.

The detailed protocols for synthesis and characterization of the different NMs and the complete datasets for each NM batch are provided as Supplementary information (S1 to S6) to the paper, allowing subsequent data mining and modelling.

## 3. Results

### 3.1. Overall Observations from the Physicochemical Characterization of NM Batches

During this project, 46 batches of NMs have been produced and extensively characterized according to the methods suggested by the OECD (2010b): 15 SiO_2_ NMs (9 Stöber, 6 Flame synthesis), 9 ZnO NMs, 8 TiO_2_ NMs, 6 PS-NH_2_ NMs and 9 CeO_2_ NMs. An overview of the NM characterization and the summary of the B2B variability is given in Table 2. For the detailed physical and chemical properties, we refer to the Supplementary tables compiled for all 46 batches of NMs.

An overview of the microstructural peculiarities, including typical TEM micrographs, of all analysed NM batches is given in the respective data summary (Supplementary Materials files S3–S8) together with the data of additionally applied SOPs. In the summary reports, the focus is on the overall characteristics of the batch—for instance agglomeration—of the different NMs and on providing detailed structural imaging of individual particles by high-resolution TEM (HRTEM) to allow any differences between the batches/synthesis conditions to be identified. Details regarding B2B variability and the influence of the particular particle synthesis are discussed in the section “Special behaviour and outliers.”

While the DLS data are reported for all NMs in the data sheets (Supplementary Materials S2 to S7), the DCS data are only reported for Stöber silica NMs (Supplementary Materials S2). The sizes reported from DLS are always larger than those measured by electron microscopy are. This is because the NMs are not monodisperse and frequently aggregated: in the DLS technique, the size distribution is weighted by the square of the particle volume and large aggregates are over estimated. The SMPS and DCS techniques allow determining the number size distributions and these diameter values are closer to them derived from electron microscopy. The agreement is rather good for silica and the amine modified polystyrene NMs. For the other NMs, the individual particles are mostly aggregated, both in solution and in aerosols. A detailed discussion of DLS and DCS data from these as well from other QualityNano NMs is presented by Langevin et al. [31].

The DCF fluorescence induced by reactive oxygen species (ROS) showed a concentration-dependent increase with most NM samples. In the summary reports only the values at the highest NM concentration (400 µg/mL) are shown to provide a better overview and comparison between the different batches and 0.3 µM H_2_O_2_ was used as a positive assay control.

Except for the flame-synthesized silica NMs (all samples), no detectable presence of NMs was found in the organic phase. Note, however, that there are challenges in the use of this method for NMs as shown by Hristovski et al. [36].

### 3.2. Highlights of Variations in Physicochemical Properties between Different Batches per NM

#### 3.2.1. Silica NMs

As shown in Figure 1F the Stöber synthesized silica NM batches SilNP03, 05, 06, 07, 09 and 10 only slightly induced DCF oxidation at 400 µg/mL (around 2-fold over control). However, batch 08 was more reactive (5.9-fold over control) and batch 04 was extremely reactive (24.2-fold over control). Further analysis of the batches by ICP-MS and ICP-OES was performed in order to identify any potential contamination that might explain the unusual behaviour of batches SilNP04 and SilNP08. Figure 1D shows the mass concentration of alkali and alkaline earth metals detected in the different batches.

The Stöber synthesized SilNP04 batch, which showed a significantly increased ROS induction compared to control (Figure 1F) also had one other noticeable characteristic: It is the only batch with a detectable amount of magnesium (Figure 1D). Different Mg concentrations in cerium dioxide NMs have previously been shown to have a modest influence on the ROS production by Iqbal et al. [37], therefore this might explain the different behaviour of SilNP04 in terms of its ROS production. For SilNP08 no conspicuous properties were detected which could explain the increased DCF fluorescence.

**Figure 2:**
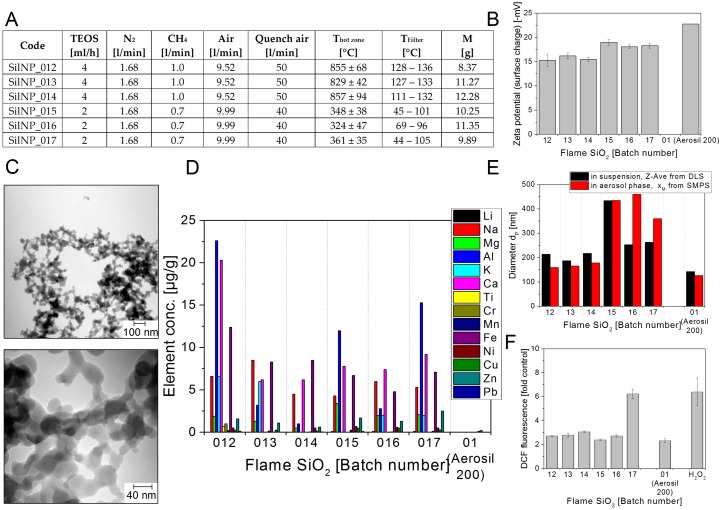
Flame synthesized silica SilNP012–017. (**A**) Production parameters for the different flame synthesized silica nanoparticles; (**B**) Zeta potential representing the surface charge in –mV at pH ~4.3; (**C**) Representative TEM images; (**D**) Selected impurities present in the different flame synthesized SiO_2_ NM batches. The mass concentrations of elements above detection limit are shown. The data represent the mean of two assays; (**E**) Particle diameters d_P_ in nm: Z-Average of d_P_ determined in aqueous suspension using DLS (black bars) and modal value x_M_ of the number size distribution in the aerosol phase measured by SMPS (red bars); (**F**) Potential to induce ROS. The test particles were delivered as powder. The graph shows the DCF fluorescence induced by the different batches at 400 µg/mL relative to a sample without NMs (control). 400 µg/mL commercial SiO_2_ (SilNP01, Aerosil^®^200, Evonik, Essen, Germany) and 0.3 µM H_2_O_2_ was used as positive controls. The data represent mean values of 2 independent experiments with three replicates ± s.e.m.

As shown in Figure 2F, the flame synthesized SilNP012 to SilNP016 moderately induced DCF oxidation (around 3-fold over control), which was comparable to the reactivity of commercial SiO_2_ (Aerosil^®^200, Evonik, Essen, Germany). Only batch 17 was more reactive (6.2-fold over control). Interestingly, none of the metals determined in batch SilNP017 stood out in terms of their concentration, meaning that the source of the high ROS production potential of this batch is not related to metal contaminants.

Data from literature showed no potential of amorphous SiO_2_ NMs, either flame or Stöber synthesized, to generate ROS in a cell-free system, while quartz particles did. The tests were performed using electron paramagnetic resonance (EPR) spectroscopy [38] but also by the DCF assay [39]. However, in a very recent study flame or Stöber synthesized NMs were tested for their potential to induce ROS, again by EPR analysis [7]. Both NMs induced ROS, however, flame synthesized NMs were much more efficient presumably due to the presence of siloxane rings at the surface. Whether the discrepancy of these different studies is due to differences in synthesis conditions needs to be addressed in the future. Additionally, whether SilNP017 had additional siloxane rings relative to the other batches and the cause of this, could be a topic for future research.

In all silica NMs, elements like Na, K, Ca, Al and Fe are the main impurities. These elements seem to be derived from both reactants and reactors. In some cases, the degree of impurities can be related to different parameters of preparation. For example, the mean total amount of impurities in silica nanopowders produced by flame synthesis (except one sample of batch SilNP_015, 31–141 µg/g) is lower by a factor of 2 than the level of impurities present in the water dispersions of Stöber silica NMs. In flame silica NMs the Cu content is ten times lower (0.2 to 0.5 µg/g) as in the Stöber batches wherein the Cu content rises from the level 1.7–6 µg/g in those samples produced at 25 °C to 11.7–18 µg/g in those produced at 5 °C. The higher Cu content in Stöber silica NMs may be caused by the reactant NH_3_.

Regarding size distribution of SiO_2_ NMs by Stöber synthesis, it was found by TEM (Figure 1C) that the distribution gets narrower with decreasing aq. ammonia concentration, for instance from 70 to 120 nm for batch SilNP_003 (2.75 g aq. ammonia concentration at 25 °C reaction temperature) to between 40 and 70 nm for SilNP_004 (2.25 g aq. ammonia). Likewise, the mean particle diameter decreases with lower aq. ammonia concentration. Also, an increase of the reaction temperature from 25 °C to 55 °C results in smaller SiO_2_ NMs (typical diameter of 20 nm). Contrary to Stöber synthesized SiO_2_ NMs, for those synthesized in the flame any particle size analysis was complicated because of extremely strong particle aggregation. Nevertheless, size and morphology of the individual primary NMs, as well of aggregated particles can clearly be resolved by TEM, which is not possible by means of integrative measurement methods like, for example, DLS.

#### 3.2.2. Zinc Oxide NMs

**Figure 3:**
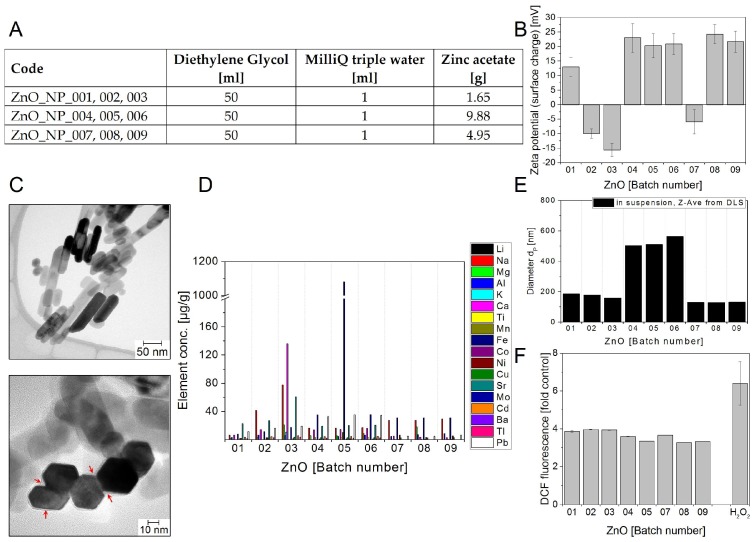
Zinc oxide NM batches ZnO NP01–09 (**A**) Production parameters for the different zinc oxide nanoparticles; (**B**) Zeta potential representing the surface charge in mV at pH ~ 6; (**C**) Representative TEM images. Regions marked with arrows seem to be amorphous material in between and around agglomerated single-crystalline ZnO nanorods; (**D**) Selected impurities present in the different flame synthesized ZnO NM batches. The mass concentrations of elements above detection limit are shown. The data represent the mean of two assays; (**E**) Particle diameters d_P_ in nm: Z-Average of d_P_ determined in aqueous suspension using DLS; (**F**) Potential to induce ROS. The test NMs were delivered as suspensions of 5 mg/mL in water. The graph shows the DCF fluorescence induced by the different batches at 400 µg/mL relative to a sample without NMs (control). 0.3 µM H_2_O_2_ was used as positive control. The data represent mean values of 2–6 replicates ± s.e.m obtained in one to two experiments.

As shown in Figure 3F all ZnO batches moderately induced DCF oxidation (around 4-fold over control) independent of the synthesis conditions (Figure 3A).

Zn is known to be a redox-inert metal which does not participate in oxidation-reduction reactions [40,41,42]. However, impurities in the sample may have caused ROS-induced DCF fluorescence, although no clear correlation with the impurities indicated in Figure 3D could be identified.

All three batches of the third production run of ZnO NMs (ZnONP007—009, 4.95 g zinc acetate) show the lowest and constant amount (<100 µg/g) of impurities, while the other runs rise to around 1000 µg/g. Depending on the further use of ZnO NMs (e.g., for toxicity testing), the presence of toxic elements such as Cu and Pb and sometimes Cd and Tl should be considered. As found by TEM inspection, at first glance, the primary sizes of the different ZnO NM batches seem to be very similar. Most of the ZnO particles are single-crystalline and have the hexagonal wurtzite structure but show amorphous material in between and around the agglomerated single-crystalline ZnO nanorods (Figure 3C). That would mean that there is no essential influence of the zinc acetate concentration. However, since the particles dominantly exhibit the shape of rods, having a length up to 300 nm and more, presumably the aspect ratio does vary with the zinc acetate concentration. This ratio seems to be higher for NMs produced at increased zinc acetate concentrations. A quantitative assessment of the relative percentage of distinct populations of ZnO NMs appearing as rods with specific aspect ratios in a given sample is quite challenging and requires a high number of individual specimens to be analysed by TEM to reach statistical significance and was therefore not performed in the present work. Nevertheless, in the future such information would be of interest to better understand the impact of geometrical parameters on toxicokinetics and adverse effects.

#### 3.2.3. Titanium Dioxide NMs

**Figure 4:**
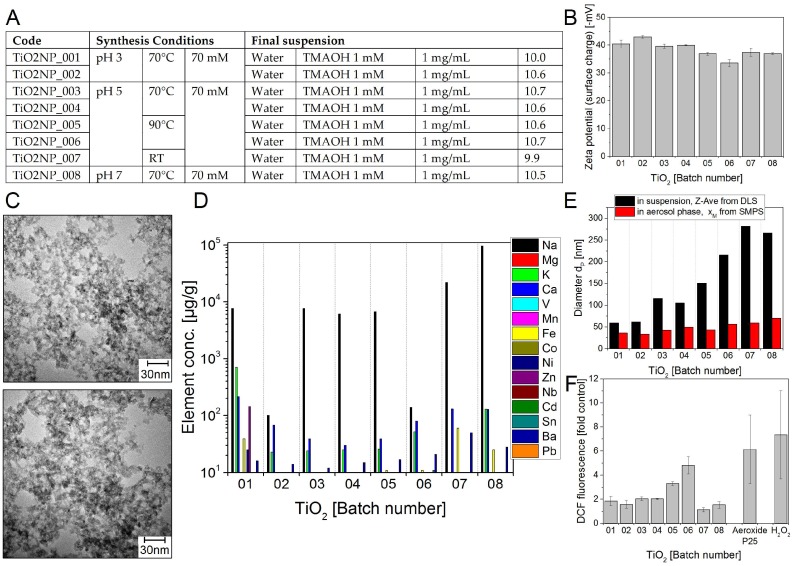
Titanium dioxide NM batches TiO_2_ NP01–08 (**A**) Production parameters for sol-gel synthesized titania nanoparticles; (**B**) Zeta potential representing the surface charge in –mV at pH ~10; (**C**) Representative TEM images (**D**) Selected impurities present in the different TiO_2_ NM batches. The mass concentrations of elements above detection limit are shown. The data represent the mean of two assays; (**E**) Particle diameters d_P_ in nm: Z-Average of d_P_ determined in aqueous suspension using DLS (black bars) and modal value x_M_ of the number size distribution in the aerosol phase measured by SMPS (red bars); (**F**) The test NMs were delivered as suspensions of 1 mg/mL in water. The graph shows the DCF fluorescence induced by the different batches at 400 µg/mL relative to a sample without NMs (control). 400 µg/mL commercial TiO_2_ (Aeroxide^®^P25, Evonik, Essen, Germany) and 0.3 µM H_2_O_2_ was used as positive controls. The data represent mean values of 2 independent experiments with three replicates ± s.e.m.

As shown in Figure 4F, TiO_2_ NP05 and TiO_2_ NP06 strongly induced DCF oxidation (3.3-fold and 4.8 fold over control, respectively), which was comparable to commercial TiO_2_ (Aeroxide^®^P25, Evonik, Essen, Germany) with a 6.1-fold value over control. The other batches were only weakly reactive (1- to 2-fold over control).

Plenty of data on ROS formation by TiO_2_ NPs using the DCF test are available in the literature [43]. Significant ROS formation by 10 nm TiO_2_ produced by sol-gel synthesis NPs was observed.

The provided TiO_2_ content in the water dispersions could be reached with synthesis conditions at pH 3, pH 5 and 70 °C or 90 °C (Figure 4A). Nevertheless, the total amount of analysed impurities is the highest of the investigated NMs. The lowest impurity content of about 230–320 µg/g show the samples TiO_2_NP_001 and _002 (Figure 4D). The twenty fold amount (6200 to 8900 µg/g) is detected in TiO_2_NP_003 to _006. In TiO_2_NP_007 and _008 the impurity content rises further up to the percentage range of 2.2 to 9.6 wt %. Na generates the main part of the impurity increase while Ca and K do not exceed the limit of 220 or 710 µg/g in sample TiO_2_NP_006. Almost all impurities were introduced during NM synthesis by sodium which should facilitate further cleaning with water. It should be investigated whether the contamination with Pb (1 µg/g), Sn (10 µg/g) and Zn (10 µg/g) has a biological impact. It is known from trace analysis that complex cleaning procedures have to be undertaken to reduce the blank level. Before reducing impurities in nanopowder production which elements are tolerable, and to what extent, should be evaluated. For all eight different TiO_2_ NM batches very tiny (e.g., 10 nm and even smaller) aggregated NMs were detected by TEM imaging. For this reason, any automated particle size determination failed and only a rough estimate of sizes was possible by directly measuring them in electron micrographs. There was no influence found of the pH value (3, 5, or 7) or the synthesis temperature (70 °C or 90 °C) during synthesis on resulting NM shape and size.

#### 3.2.4. PS-NH_2_ NMs

**Figure 5:**
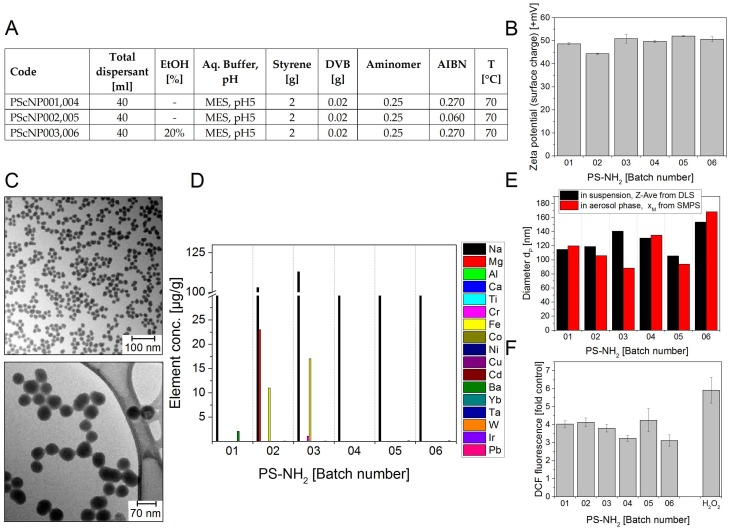
PS-NH_2_ nanoparticle batches PS NP01–06. (**A**) Production parameters for sol-gel synthesized polystyrene nanoparticles; (**B**) Zeta potential representing the surface charge in +mV at pH 6.1; (**C**) Representative TEM images (**D**) Selected impurities present in the different PS NM batches. The mass concentrations of elements above detection limit are shown. The data represent the mean of two assays; (**E**) Particle diameters d_P_ in nm: Z-Average of d_P_ determined in aqueous suspension using DLS (black bars) and modal value x_M_ of the number size distribution in the aerosol phase measured by SMPS (red bars); (**F**) Potential to induce ROS. The test particles were delivered as suspensions of 10 mg/mL in water. The graph shows the DCF fluorescence induced by the different batches at 200 µg/mL concentration relative to a sample without NMs (control). 0.3 µM H_2_O_2_ was used as positive control. The data represent mean values of 2 independent experiments with three replicates ± s.e.m.

For PS-NH_2_ NPs the standard test procedure had to be changed. The centrifugation time was enhanced to 1 h due to the low density of the particles. The samples showed a clear increase in DCF oxidation (3.2- to 4.2-fold over control) (Figure 5F). No significant outliers were observed in the DCF signal, nor in the analysis of impurities.

#### 3.2.5. Cerium Dioxide NMs

**Figure 6:**
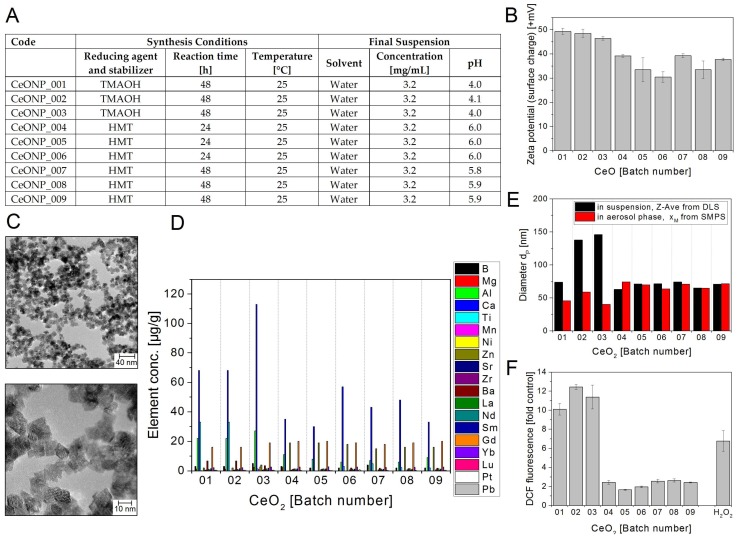
Cerium dioxide NM batches CeO_2_ NP01–09 (**A**) Production parameters for sol-gel synthesized CeO_2_ nanoparticles; (**B**) Zeta potential representing the surface charge in +mV at pH 3.8 to 6.1; (**C**) Representative TEM images of CeONP_008; (**D**) Selected impurities present in the different CeO_2_ NM batches. The mass concentrations of elements above detection limit are shown. The data represent the mean of two assays; (**E**) Particle diameters d_P_ in nm: Z-Average of d_P_ determined in aqueous suspension using DLS (black bars) and modal value x_M_ of the number size distribution in the aerosol phase measured by SMPS (red bars); (**F**) Potential to induce ROS. The test particles were delivered as suspensions of 3.2 mg/mL in water. The graph shows the DCF fluorescence induced by the different batches at 400 µg/mL relative to a sample without NMs (control). 0.3 µM H_2_O_2_ was used as positive control. The data represent mean values of 2 independent experiments with three replicates ± s.e.m.

As shown in Figure 6F, CeO_2_NP01–03 strongly induced DCF oxidation (up to 12-fold over control), while CeO_2_NP04–09 were only weakly reactive (around 2-fold over control).

The level of the DCF oxidation (Figure 6E) was also compared with the amount of chemical impurities (Figure 6D). In the three batches with increased DCF signal the mass concentrations of aluminium were increased compared to the six others. Increased values of calcium were also observed but these are not proportional and additionally, calcium is also increased in batch 6, which has no strong increase in the DCF signal.

The second lowest total amount of impurities of all NMs tested was detected in the CeO_2_ nanopowder dispersions and did not exceed 190 µg/g. The third production run to obtain CeO_2_ NP007–009 reached almost 2.8 mg/mL compared to the calculated CeO_2_ content of 3.2 mg/mL and this series had the lowest and constant impurity content (100 µg/g). The use of HMT correlates with the higher Zn amounts in the batches 04 to 09. TEM characterization showed more or less regular ceria nanocrystals with sizes in the range from 3 to 20 nm.

## 4. Discussion

For safety assessment of NMs, laboratories performing the tests require reference materials of highest quality in sufficient quantities. In addition, applications of NMs which imply potential contact with the human body need to be safe (e.g., at the work place or drug carriers for medical treatment) and such features can be designed in if the relevant physicochemical NM properties can be defined and controlled during NM synthesis. 

Identification of potential sources of variability of NM batches and their reduction is therefore an important task. As a first step towards this goal, OECD proposed parameters for NM characterization have been measured for numerous batches of NMs of different core compositions. These batches were prepared under carefully controlled conditions to relate the synthesis conditions to the measured results.

The following main conclusions based on different parameters can be drawn based on this work and on previous studies [44,45,46,47,48]. A comparison of the B2B data to the data reported by JRC is given in the Supplementary Material S8.

### 4.1. Size Distributions

- Depending on the synthesis conditions, the size distributions of NM batches can be reasonably well controlled with low variability and high reproducibility.
- The particle size distributions of NMs from liquid phase synthesis are narrow, however partial agglomeration is observed, especially for ZnO, TiO_2_ and CeO_2_ NMs, both in solution and in aerosols.
- Silica materials from flame synthesis are mostly aggregated and have broader size distributions.
- The particle sizes determined by DLS are usually larger and more variable even for NMs produced by the same synthesis route than the diameters determined by SMPS. Moreover, some particles become de-agglomerated by spraying them into the gas phase and drying afterwards, on the other hand further agglomeration could occur in solution after the synthesis.
- Post-synthesis treatments commonly applied in the purification of NM dispersions, such as centrifugation approaches, can cause shifts in dispersion size distributions due to particle aggregation [49].
- For liquid phase synthesis, dispersion reproducibility can be “designed into” the synthetic procedure by following optimization processes which involve careful parameter variation as precursor concentrations or temperature (see Supplementary Materials S1) coupled with extensive characterization.

### 4.2. Zeta Potential

Irrespective of the synthesis conditions, the zeta potential is fairly constant and is also highly reproducible also for batches produced by the same synthesis route except for zinc oxide, which vary between −15 and +24 mV at a constant pH ~ 6.

In contrast, Singh et al. and Rasmussen et al. reported zeta potential values covering a wide range (S8). For silica, the values were comparable to our results. However, for titania −40 to more than +30 mV were measured [45] and for ceria between −7 and +33 mV [46]. As shown by Rasmussen et al. the zeta potential is highly dependent on the pH value of the suspension, which might explain to some extent the differences observed.

### 4.3. Crystallinity

It is well known that differences in the crystalline phase of NMs with the same composition may affect their toxic effects. This has been shown for example, SiO_2_ NPs where the crystalline quartz particles are more toxic than amorphous silica NMs [50]. In this study, all SiO_2_ preparations were amorphous. The TiO_2_ NM in this study were found to consist of 80% anatase and 20% brookite, a similar content of anatase as in the reference NM Aeroxide^®^P25 which consists of 80% anatase and 20% rutile. It is known that the anatase form is chemically more reactive and more toxic than the other crystalline forms [51]. However, only batch 06 (Figure 4F). showed a similar radical formation potential as Aeroxide^®^P25. All other batches induced lower radical formation. Thus, the crystallinity alone does not entirely predict ROS formation.

### 4.4. Impurities

- The batches produced by liquid phase synthesis are partly contaminated by low concentrations of metals (e.g., Cu, Al, Mg) which are too low to be of toxicological concern and of which the sources remain unclear but most likely they were present in the starting reagents.
- Silica nanoparticles from flame synthesis have a higher chemical purity than the ones produced by Stöber synthesis. Due to the use of smaller amounts of precursors, mostly pure gases, the particles formed in a high temperature process are therefore of higher purity. This is in agreement with recent studies by Rasmussen et al. comparing precipitated synthetic amorphous silica (SAS) with fumed SAS [46].
- The levels of impurities and the main elemental concentrations in water dispersions (TiO_2_, CeO_2_, Stöber silica, polystyrene and ZnO) vary from batch to batch dependent on different synthesis parameters. However, also in batches synthesized in the same way (Stöber silica, TiO_2_ and ZnO) the composition and amount of impurities varied. Yet, interestingly for ceria produced by the same synthesis route this variability of trace contaminants was largely absent.
- Ca was most often detected as impurity at higher levels. Similar findings were reported by Rasmussen et al. and by Singh et al. for the JRC representative manufactured NMs [45,46,47,48]. The dispersant (water) has been suggested as a potential source.

### 4.5. ROS Generation

The radical formation potential of NMs is an important intrinsic parameter for predicting the formation of ROS in cells and organisms. Together with cellular ROS production (e.g., activation of NADPH oxidases) this is considered as a key event in inducing oxidative stress and adverse effects such as oxidative damage of proteins, lipids or DNA. Different detection methods of the radical formation potential have been developed. They are summarized in a recent review by Hellack et al. [52] and strengths and weaknesses of the singe assays were discussed. The DCF assay was evaluated as suitable in principle, however, other methods seem to be more sensitive in a cell-free system. The authors proposed to use multiple methods to identify potential artefacts in individual assays. They furthermore found that some types of NMs caused oxidative stress in cells while producing low amounts of radicals in a cell-free assay. Other NMs triggering marked cell-free radical formation induced only minimal oxidative stress in cells [53]. Therefore, a correlation between radical formation potential of NMs and toxicity is difficult and should be studied for each type of NM.

In the OECD WPMN testing program, a suite of techniques was evaluated to determine physico-chemical properties of NMs [14,15]. With respect to radical formation, methods different to the DCF assay were assessed for silica, ceria, zinc oxide and titania. Electron spin resonance seemed to be most appropriate to detect radical formation by the respective NMs. Here we used the DCF assay which also indicates reactivity of these NMs but also of PS-NH_2_.

**Figure 7:**
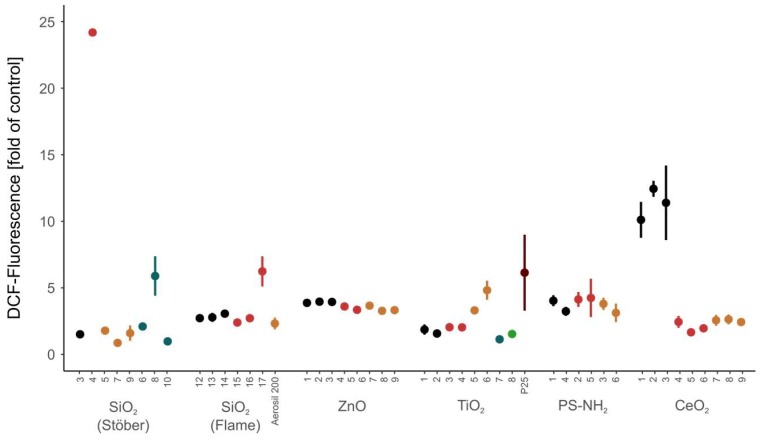
Comparison of the ability of all tested NMs to induce DCF fluorescence. Same colour code within one NM type indicates that the NMs have been synthesized by the same method.

Figure 7 shows a comparison of the ability to induce DCF fluorescence of all tested NMs which indicates their potential to generate ROS. Some NMs such as ZnO and PS-NH_2_ moderately induced DCF fluorescence but showed no difference between the batches. The other NMs showed a moderate (silica-flame, titania) to high (silica-Stöber, ceria) B2B variability when assessing the formation of ROS in a cell-free assay. As ROS production is one of the central mechanisms to explain and predict toxicity of NMs, in the future there is certainly a need to investigate and understand the basis for this B2B variability. Correlation between impurity level and ROS generation was found in some cases but not in general. Note that in the case of carbon NMs, the correlation between toxicity and impurity level is well recognized [54,55].

For silica, either produced by flame or colloidal synthesis, outliers could be identified with strong potential to produce ROS. Although the reason for this different behaviour could not be resolved, also in previous studies with colloidal silica a high B2B variability of the surface chemistry, which is critically linked to ROS production, could be observed [56]. Therefore, future studies should correlate differences in ROS formation as measured here in cell-free assays with toxicity for example, in cells to investigate the relevance of reactivity as proposed by the OECD WPMN for safety assessment in case by case studies.

It is likely that other parameters such as size distribution, crystallinity and surface properties also play a role in ROS generation. Concerning the surface properties, the surface charge does not seem to be significantly affected by the preparation method, as the zeta potential varies only with the pH of the solutions. Interestingly, clustering of the batches produced by the same synthesis conditions revealed in the case of ceria a strong correlation of lower DCF oxidation with the presence of the reducing agent HMT. Overall, in the case of ROS formation there seems to be a higher B2B variability between batches produced by different synthesis conditions compared to batches produced in the same way, although other parameters such as size and the zeta potential are fairly similar.

Finally, also some technical recommendations can be made to characterize B2B variability:

- As the NMs are frequently agglomerated or even aggregated, the particle size distributions should be measured by several methods such as DLS and/or SMPS/electrospray and TEM to minimize the variability of the determined size due to technical limitations. In the case of TEM, dedicated methods, which combine manual masking means and automated digital image analysis routines, have to be developed for particle-size determination.
- In general, particle size distributions measured by SMPS/electrospray yield more reproducible data than DLS measurements especially for agglomerated nanomaterials.
- Questions remain around the reproducibility of measurement techniques such as DLS for non-ideal (polydispersed) NM dispersions. This can be observed especially for agglomerated/aggregated NM types when dispersing from dry powders where the goal is limited to producing homogenously aggregated populations.
- The size distribution and crystallinity of the primary particles in agglomerates and aggregates should be characterized by HRTEM/SEM.
- ICP-MS and ICP-OES are valuable tools to identify the variability of NM batches due to contamination by trace metals.

To summarize, whereas the variability of a number of parameters suggested by the OECD WPMN is rather low that is, the values are rather stable and reproducible such as the zeta potential and size others for example, impurities are less well controlled. Specifically, ROS formation as an indicator of reactivity seems to vary especially for batches produced by different conditions although other parameters remain constant. Given the importance of ROS levels in adverse responses the sources for this B2B variability deserve further investigations. Specifically, the reliability of different assays to monitor radical formation needs be compared and validated in the future and correlated with biological activity in order to provide predictive parameters for hazard assessment. Overall, we confirm for a wider range of NMs the suitability of proposed techniques to characterize physico-chemical properties of NMs. Yet, with respect to reactivity and biological activity there are still uncertainties as to which techniques to use and physico-chemical properties to select. Although of academic interest to design and control such properties in the future, NMs already on the market need to be tested as produced for example, in an agglomerated state to address the impact of physico-chemical properties on material safety.

## Figures and Tables

**Table 1:** Analytical methods chosen to characterize the particle properties.

Particle Property	Analytical Method	References	Supplementary Materials
Trace impurities	Optical Emission Spectrometry with Inductively Coupled Plasma (ICP-OES),	-	S1-1
	Mass Spectrometry with Inductively Coupled Plasma (ICP-MS)		
Water Solubility	Concentration of free ions in solution	[28]	S1-2
Crystalline phase	Electron Microscopy and Focussed Ion Beam Nanostructuring	-	S1-3
Crystallite size	X-ray Diffraction (XRD)	-	S1-4
Primary particle size	Transmission Electron Microscopy (TEM)	-	S1-6a,b
Agglomeration/aggregation state	Transmission Electron Microscopy (TEM)	-	S1-6a,b
Morphology	Transmission Electron Microscopy (TEM)	-	S1-6a,b
Diameter of aerosolized NMs	Electrospray aerosol generator coupled with Scanning Mobility Particle Sizer (SMPS)	[29]	S1-7
Hydrodynamic diameter	Dynamic Light Scattering (DLS)	[30,31]	S1-8a,b
Porosity and surface area	Gas sorption analysis (BET method)	-	S1-9
Surface charge	Zeta potential	-	S1-10
Photocatalytic activity	UV-Vis spectrophotometer	-	S1-11
Octanol-water partition coefficient	Mass Spectrometry with Inductively Coupled Plasma (ICP-MS)	-	S1-12
Radical formation potential	Dichlorofluorescein (DCF) assay	[32,33]	S1-13

**Table 2:** Summarized physicochemical parameters characterized for the five selected priority nanomaterials (NMs), highlighting the B2B variability.

Physicochemical Parameter	Silica NMs (Stöber Synthesis)	Silica NMs (Flame Synthesis)	Zinc Oxide NMs	Titanium Dioxide NMs	Amine-Modified Polystyrene NMs	Cerium Dioxide NMs
Varied synthesis parameter	Concentration of NH_3_ and aqueous ammonium	TEOS concentration in the flame and flame conditions	Zinc acetate concentration	pH value and temperature	Concentrations of Azobisisobutyronitrile and ethanol	Reducing agent and reaction time
Number of batches	8	6	9	8	6	9
Trace impurities (Main elements)	Up to 67 µg/g (Ca and Na)	Up to 75 µg/g (Al)	Up to 1080 µg/g (Fe, Ca, Na and Sr)	Up to 96 mg/g (Na)	42 to 113 µg/g (Na)	87 to 187 µg/g (Ca)
Dissolution in water	n.d.	6 to 10 wt %	7 wt %	n.d.	n.d.	n.d.
Crystal structure	Amorphous	Amorphous	Crystalline: hexagonal zinc oxide or wurtzite	Crystalline: anatase with a small fraction of brookite	Amorphous	Crystalline: cubic lattice
Crystal size	--	--	20 to 30 nm	4.5 to 6 nm	--	5 to 8 nm
Mobility diameter	33 to 118 nm	32 to 160 nm.	25 to 55 nm	33 to 70 nm.	88 to 170 nm.	40 to 74 nm.
Hydrodynamic diameter (DLS)	25 to 120 nm	200 to 380 nm	130 to 900 nm	60 to 280 nm	105 to 150 nm	63 to 146 nm
Zeta potential (surface charge)	−45 to −66 mV (pH 8.4 ± 0.57)	−15 to −18 mV (pH ~ 4.3)	−15 to +24 mV (pH ~ 6)	+33 to +40 mV (pH ~ 10)	+44 to +50 mV (pH 6.1)	+30 to +49 mV (pH 3.8 to 6.1)
Photocatalytic activity	No	No	High	Low	No	No
Octanol- water partition coefficient	Not analysed	<detection limit	<detection limit	<detection limit	<detection limit	<detection limit
Radical formation	2–24.2-fold of control	2–6.2-fold of control	3- 4-fold of control	1–4.8-fold of control	3.2- 4.2-fold of control	2- 12-fold of control
Physicochemical characterization data sheets	Supplementary Materials document S2	Supplementary Materials document S3	Supplementary Materials document S4	Supplementary Materials document S5	Supplementary Materials document S6	Supplementary Materials document S7

## Supplementary Materials

The following are available online at http://www.mdpi.com/2079-4991/8/5/311/s1, document S1: Standard operation procedures (SOP) for physicochemical characterization, document S2: Physicochemical characterization data sheets for silica NMs from Stöber synthesis, document S3: Physicochemical characterization data sheets of silica NMs from flame synthesis, document S4: Physicochemical characterization data sheets of zinc oxide NMs, document S5: Physicochemical characterization data sheets of titania NMs, document S6: Physicochemical characterization data sheets of PS-NH_2_ NMs, document S7: Physicochemical characterization data sheets of ceria NMs, document S8: Comparison of B2B data to JRC reported data.

## Abbreviations

B2B	Batch-to-batch
DEG	Diethylene glycol
DLS	Dynamic Light Scattering
DCS	Differential centrifugal sedimentation
DCF	Dichlorofluorescein [= oxidized form of 2′,7′-dichlorofluorescein diacetate (H2DCF)]
DVB	Divinylbenzene
EDX	energy dispersive X-ray analysis
EPR	Electron paramagnetic resonance
HMT	hexamethylenetetramine
HRTEM	high-resolution Transmission Electron Microscopy
ICP-MS	mass spectrometry with inductively coupled plasma
ICP-OES	optical spectrometry with inductively coupled plasma
MES	2-(*N*-morpholino)-ethanesulfonic acid
MWCO	Molecular weight cut-off
NM	Nanomaterial
OECD	Organization for Economic Cooperation and Development
PES	Photoemission spectroscopy
rcf	relative centrifugal force
ROS	Reactive Oxygen Species
SEM	Scanning Electron Microscopy
s.e.m.	Standard error of the mean
SMPS	Scanning Mobility Particle Sizer
SOP	standard operation procedure
TEM	Transmission Electron Microscopy
TEOS	Tetraethylorthosilicate
TMAOH	Tetramethylammonium hydroxide
