# Supplementary material for: Characterization of Nanoparticle Batch-To-Batch Variability
Source: Nanomaterials (Basel). 2018 May 8;8(5):311. doi: 10.3390/nano8050311 (PMC5977325; doi:10.3390/nano8050311)
Supplement: Supplementary file 1 [file nanomaterials-08-00311-s001.pdf]

Supplemental S1: Standard operation procedures (SOP) for physico-chemical characterization

S1-1 Trace impurities

Inductively Coupled Plasma Mass Spectrometry (ICP-MS)

Inductively Coupled Plasma Optical Emission Spectrometry (ICP-OES)

Technology:
Mass an Optical Emission Spectrometry of Materials after dissolution in acids

Equipment:

- ICP-MS: 7500 ce, Agilent
- ICP-OES OPTIMA 4300DV Perkin Elmer

Category:
C. Particle Characterisation ex-situ

Institute: KIT
Location:
Karlsruhe Institute of Technology
Institute for Applied Materials (IAM-AWP)
Building 688
Hermann-v.-Helmholtz-Platz 1
D-76344 Eggenstein-Leopoldshafen
Germany

Contact Details of Technology Expert:
Name: Dr. Christel Adelhelm
Phone: +49 721 608-22914
Fax: +49 721 608-922914
E-mail: christel.adelhelm@kit.edu

Short technology description and Main Features:

ICP-MS is a sensitive analytical method (0.000001 – 100%) for rapid multi-element (Li - U except C, N, O, F, Cl) determination in the trace and ultra-trace range of different solid and liquid sample materials, technical products and biological samples. Solid samples are dissolved by subboiled acids. The liquids are aspirated into a spray-chamber, dried, atomized and ionized in an Ar-Plasma (ICP). The element ions are accelerated into a quadrupole mass spectrometer (MS) and detected according to their mass. To eliminate mass interferences the ions pass a reaction cell. The detector intensities are correlated to the intensities of calibration solutions. ICP-OES is a less sensitive (0.0001 – 100%) but robust and precise method for multi-element analysis (Li - U except C, N, O, Halogens). Sample introduction is similar to ICP-MS. In the Argon plasma excited atoms or ions emit light dispersed by an Echelle optic and detected by a segmented diode array. Calibration solutions provide quantification.

Typical Samples & Images:

H																	He
Li 0,002	Be 0,0005											B 0,01	C	N	O	F	Ne
Na 0,03	Mg 0,002											Al 0,005	Si 5	P 1	S 10	Cl 5	Ar
K 5	Ca 0,5	Sc 0,005	Ti 0,01	V 0,002	Cr 0,005	Mn 0,0005	Fe 5	Co 0,0005	Ni 0,01	Cu 0,01	Zn 0,002	Ga 0,001	Ge 0,002	As 0,01	Se 0,02	Br 0,5	Kr
Rb 0,0005	Sr 0,0001	Y 0,0002	Zr 0,0005	Nb 0,0005	Mo 0,001	Tc	Ru 0,0005	Rh 0,0002	Pd 0,001	Ag 0,0001	Cd 0,0005	In 0,0001	Sn 0,0005	Sb 0,001	Te 0,002	J 0,002	Xe
Cs 0,001	Ba 0,0005	La 0,0001	Hf 0,0005	Ta 0,0005	W 0,0005	Re 0,0005	Os 0,01	Ir 0,0005	Pt 0,001	Au 0,0005	Hg 0,001	Tl 0,0001	Pb 0,001	Bi 0,0002	Po	At	Rn
		Ce 0,0001	Pr 0,0001	Nd 0,0005	Pm	Sm	Eu 0,0002	Gd 0,0002	Tb	Dy 0,0005	Ho 0,0001	Er 0,0005	Tm 0,0002	Yb	Lu 0,0001		
Fr	Ra	Ac	Th 0,0002	Pa	U 0,0002	Np	Pu	Am	Cm	Bk	Cf	Es	Fm	Md	No	Lw	

Typical detection limits of ICP-MS in µg/L

Any further Information

The most sensitive analytical methods have been chosen to determine most other elements than the nanomaterials consisted of. Two methods have been used ICP-OES (optical emission spectrometry with inductively coupled plasma) for elements with concentrations above 10 ng/mL or ICP-MS (mass spectrometry with inductively coupled plasma) below 10 ng/mL. Because of the sensitivity of these methods precleaned vessels, subboiled or ultrapure acids and deionized water (18 Ω/cm^2) have been used. To identify matrix effects and spectral interferences the purest available substances of SiO_2, Ti metal, ZnO or $Ce(NO_3)3*(6-7)H_2O$ have been analyzed to. Blank solutions have been prepared with the same acid and water to control the blank.

The main compounds have been determined additionally either to show the batch to batch variability and/or to correlate the impurity concentration. For precise element determination ICP-OES and the use of internal standard provides the lowest repeatability.

Nanomaterials dispersed in water or as powder has to be dissolved in acids previous to spectrometric investigation:

SiO_2: 0.4 g nanopowder was dissolved in 2 mL HF ultrapure and diluted to 100 mL or 5 mL of homogenized dispersion was acidified with 0.5 mL HF ultrapure and diluted to 15 mL with water. For ICP-MS further dilution was necessary.

ZnO: 0.05 g nanopowder was dissolved in 2 mL HCl subboiled at 80 °C and made up to volume 50 mL with deionized water. For ICP-MS further dilution was necessary.

TiO_2: 5 mL of homogenized dispersion was acidified with 1 mL HF ultrapure and 0.2 mL HNO_3 subboiled. After heating at 80 °C the clear solution was diluted to15 mL with deionized water. For ICP-MS further dilution was necessary.

CeO_2: 5 mL of homogenized dispersion was acidified with 2 mL HNO_3 subboiled and H_2O_2 suprapure .After heating at 80 °C the clear solution was diluted to10 mL with deionized water. For ICP-MS further dilution was necessary.

S1-2 Solubility

Water Solubility- dissolution test	Category:<br>C. Particle Characterisation in and ex-situ and/or<br><br>Institute: Institut Català de Nanotecnologia (ICN)<br>Location: Campus UAB<br>Contact Details of Technology Expert:<br>Jordi Piella<br>Tel. +34-937374624<br>Jordi.piella@icn.cat

Short technology description

Water solubility is defined as the saturation mass concentration of the substance in water at a given temperature. However, when talking about nanoparticles it may be a relatively misleading term and a more accurate definition is necessary. Two main processes exist when adding nanoparticles in a specific environment: dispersion and dissolution. The first one is related to the number of particles in suspension, while the second one refers to the release of ions form the particles. The OCDE procedure does not clearly differentiate these two concepts. Moreover, dissolution and dispersion are often mutually affected. In the present project, the solubility of a nanoparticle is understood has the concentration of free ions in the solution in equilibrium with the particle in a controlled environment.

Material

All experiments are performed in Milli-Q® water. Concentration of ions in the sample is determined spectrophotometrically and the standard procedure depends on the ions to be determined.

Procedure

Test is performed in Milli-Q® water at 25°C, circumneutral pH and under static equilibrium conditions to avoid interferences. The concentration of the substance is chosen to a value that it is approximated to a concentration used for in vitro experiments.

The sample is diluted with Milli-Q® water until a concentration of 100 ppm and incubated at 25°C. If it is in powder form, the solution is sonicated (Brandson 2510) for 15 min to achieve well dispersion. Aliquots are taken at different time points (until 30 days), centrifuged at high speed and the supernatant filtrated with the use of a regenerated cellulose centricone (Millipore) to ensure removal of all the nanoparticles from the sample. The amount of ions absorbed by the filter is calculated with the use of standards. Finally, the concentration of free ions is measured spectrophotometrically (UV-Vis spectrophotometer, Shimadzu UV-2401) with the corresponding standard procedure for each substance (SiO_2[13], ZnO[14], TiO_2[15, 16]). Due to a lack in the procedure, tests for Polystyrene and CeO_2 nanoparticles were not done. Further discussion is presented in later sections.

Main Features (Equipment capabilities):

UV-Vis spectrophotometer (Shimadzu UV-2401) for absorbance measurement from 200 nm to 900 nm

Typical Samples & Images:

Any further Information:

- OECD guideline for the testing of chemicals. Test 105.[12]
- 4500-SiO_2 D. Heteropoly Blue Method: Greenberg, A. E., Standard methods for the examination of water and wastewater. 16th edition. American Public Health Association, Washington, 1985.
- 3500-Zn F. Zincon Method: Greenberg, A. E., Standard methods for the examination of water and wastewater. 18th edition. American Public Health Association, Washington, 1985.

S1-3 Crystalline phase

Electron Microscopy and Focussed Ion Beam Nanostructuring

Technology**:**

- Scanning Electron Microscopy (SEM)
- Transmission Electron Microscopy (TEM)
- Focussed Ion Beam (FIB) Nanostructuring

Equipment:

- TEM FEI TITAN 80-300 with Cs image corrector, EDX spectrometer, monochromator, and high-resolution imaging energy filter
- Philips CM200 FEG/ST
- ZEISS922 Omega
- Field-emission (FE)-SEM FEI Quanta 650 ESEM
- FE-SEM ZEISS LEO 1530
- FIB FEI Strata 400

Category:
C. Particle Characterisation in- and ex-situ

Institute: Laboratory for Electron Microscopy (LEM), KIT

Location:
Laboratory for Electron Microscopy (LEM)
Karlsruhe Institute of Technology (KIT)
Campus South, Building 30.22
Engesserstr. 7
D-76131 Karlsruhe
Germany

Contact Details of Technology Expert:
Name: Dr. Reinhard Schneider
Phone: +49 721 608-43719
E-mail: reinhard.schneider@kit.edu

To determine the inner structure of solid-body particles, transmission electron microscopy (TEM) techniques are a well-established analytical means besides, e.g., X-ray diffraction (XRD) (Figure 1a). In more detail, the real structure of a material can directly be determined by high-resolution TEM (HRTEM) imaging, where the contrast features do clearly reveal whether the particles are amorphous or crystalline.

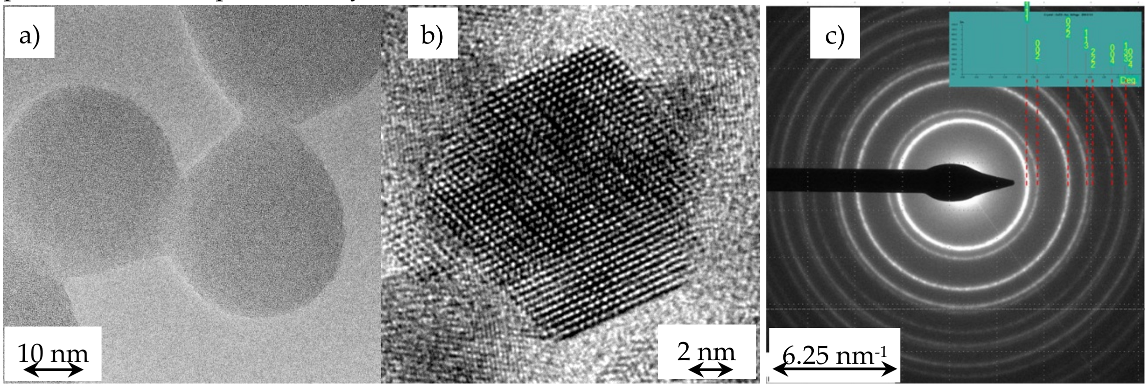

Fig. 1: HRTEM image of silica (a) and CeO_2 nanoparticles (b), SAED pattern recorded from a large number of CeO_2 nanoparticles (c).

Exemplary, this can be recognized from the HRTEM images in Figs. RS1a and b), showing SiO_2 nanoparticles of the batch SilNP002 with amorphous structure and an individual single-crystalline CeO_2 particle (CeO_2NP_006) of approximately 12 nm in size, respectively. For the latter particle the viewing direction is along the [110] zone axis and the corresponding distances of visible {111} and {002} lattice planes amount to about 0.312 nm and 0.271 nm. Nowadays, through the use of state-of-the-art instruments equipped with electron-optical systems for correcting the spherical aberration of the objective lens, a lateral resolution of 0.1 nm and better can be achieved, which allows to visualize clusters of atoms or even single metal atoms lying on extremely thin carbon films (Figure 1b, Figure 1c). However, the accuracy in measuring the crystal-lattice spacing strongly depends on the quality of the microscope calibration, which is usually done by reference samples with known structure.

Keeping this in mind, differences in determining lattice parameters from laboratory to laboratory can also be caused by not well performed calibration procedures. In general, reliable structural data with ± 0.005 nm as a typical number can be obtained by HRTEM imaging. Thus, XRD measurements, yielding lattice parameters in nanometers with uncertainties in the 5th digit, are more accurate [RS5]. In addition to imaging techniques, structure analyses are also possible in TEM by applying electron-diffraction methods as given via the selected-area electron diffraction (SAED) pattern of ceria nanoparticles (batch CeO_2NP_009) in Fig. RS1c. In this figure the experimentally obtained SAED pattern is compared with a simulated one, which distinctly furnishes proof of the presence of CeO_2. Thus as shown in this example, electron diffraction gives information not only about the lattice parameters and symmetry of a crystal, but also about the stoichiometry of a compound, i.e. the present chemical phase.

Any further Information:

S1-4 Crystalline Size

<table>
<tr><td>Equipment Name:

XRD</td><td>Category:
C. Particle Characterisation in and ex-situ
Institute: Institut Català de Nanotecnologia (ICN)
Location: Campus UAB
Contact Details of Technology Expert:
Jordi Piella
Tel. +34-937374624
Jordi.piella@icn.cat</td></tr>
</table>

Short technology description / Overview

Maybe the most versatile and widely used characterization tool for materials science and now also for nanotechnology. XRD is based on the optical interferences when a monochromatic radiation passes through a gap that has the same length than its wavelength. As X-Ray have similar wavelengths than the interatomic lengths inside crystals (in the order of Angstroms) when pass through a crystal, this acts as a diffracting net: diffracts the x-rays with a concrete direction and intensity and a pattern of peaks is obtained

Procedure

XRD measurements are performed using a powder diffractometer (PANalytical X′Pert) operating with a Cu Kα radiation source (λ=1.541Å). If the sample is suspended in liquid phase, the solid is extracted out by centrifugation and dried in an oven at 70°C to eliminate all the moisture.

For crystalline size determination, D_{hkl} is calculated applying the Scherrer formula, $D_{hkl} = 0.9\ \lambda/B_{hkl} \cos\theta$, to the line width, B_{hkl}, at half maximum corrected for the instrumental broadening assuming Gaussian profiles.

Equipment:

- PANalytical X′Pert diffractometer operating with a Cu Kα radiation source (λ=1.541Å) or Co Kα (λ=1.789Å).
- Composition of the NPs (and purity)
- Arrangement of the atoms inside the NPs (crystalline phase)
- Size of the crystal using Scherrer's formula
- Size, crystal face, purity, crystal defects, degree of oxidation
- Sonicator (Branson 2510)
- Centrifuge (Hermle Z36HK)

Typical Samples & Images:

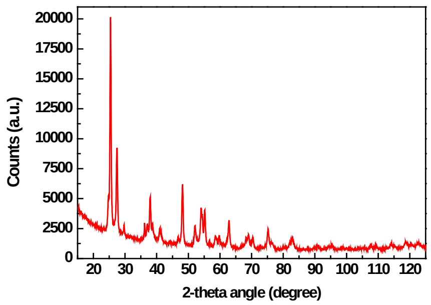

Figure 1: XRD spectrum of commercial Degussa P25 purchased from Sigma-Aldrich.

Any further Information:

S1-5 Crystallite-size / particle-size distribution by TEM

Electron Microscopy and Focussed Ion Beam Nanostructuring

Technology:

- Scanning Electron Microscopy (SEM)
- Transmission Electron Microscopy (TEM)
- Focussed Ion Beam (FIB) Nanostructuring

Equipment:

- TEM FEI TITAN 80-300 with Cs image corrector, EDX spectrometer, monochromator, and high-resolution imaging energy filter
- Philips CM200 FEG/ST
- ZEISS922 Omega
- Field-emission (FE)-SEM FEI Quanta 650 ESEM
- FE-SEM ZEISS LEO 1530
- FIB FEI Strata 400

Category:
C. Particle Characterisation in- and ex-situ

Institute: Laboratory for Electron Microscopy (LEM), KIT

Location:
Laboratory for Electron Microscopy (LEM)
Karlsruhe Institute of Technology (KIT)
Campus South, Building 30.22
Engesserstr. 7
D-76131 Karlsruhe
Germany

Contact Details of Technology Expert:
Name: Dr. Reinhard Schneider
Phone: +49 721 608-43719
E-mail: reinhard.schneider@kit.edu

Because of its high lateral resolution, TEM is certainly well suited for measuring the size of an individual nanoparticle. However, problems can arise when nanoparticles are strongly agglomerated, hindering to distinguish between single particles. Moreover, the specific shape of a particle – i.e. spherical, plate-like, completely irregular, and so on – can complicate particle-size analyses since then a definition is needed what "particle size" does mean. Hence, from this point of view the simplest and therefore also best case for particle-size analysis is well-separated spherical particles. Here, automated routines of digital image analysis can often be applied successfully to find the size distribution. For more complicated configurations like, e.g., agglomerated particles or particles with elongated form, there are sometimes solutions of the above-mentioned problems which combine manual analyses with automated ones. Few corresponding examples of our QNano project, namely where fully digital routines failed, are presented in the following.

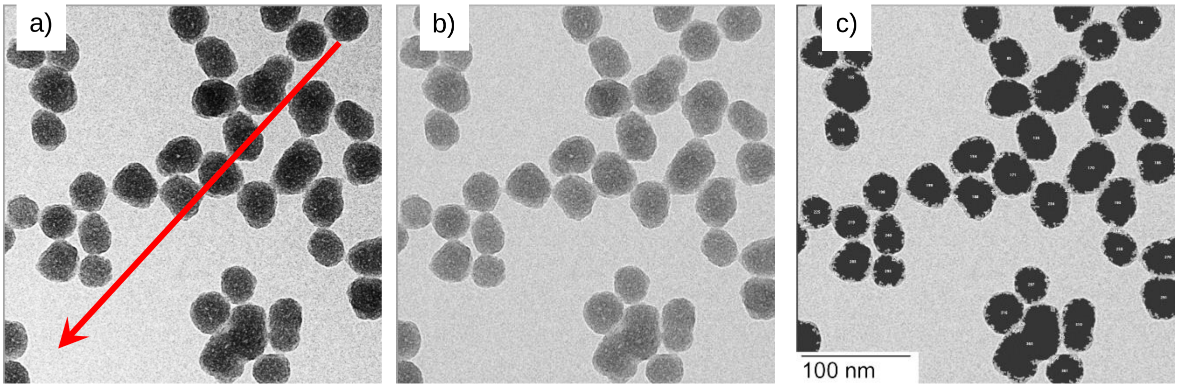

Fig. 1: Original TEM image of silica nanoparticles (a), image after background equalization (b), and with marked particles by applying a gray-level threshold (c).

The first example of silica nanoparticles concerns agglomeration. As demonstrated in Fig. 1, even for slightly agglomerated nanoparticles the sizes cannot be measured automatically. Fig. 1a shows the raw-data image taken from the SiO_2 particles (batch SilNP007), exhibiting a nearly similar size. Obviously, they touch each other which causes problems in the automatic particle separation.

In addition, before performing any digital image analysis, the background-brightness level has to be corrected in the case of not evenly illuminated pictures (there is a gradual increase in intensity from the upper right to the lower left, see arrow in Fig. 1a). This can be done by suited correction tools implemented within different image-processing programs (e.g., Adobe Photoshop). Then, after this background equalization (Fig. 1b) a particle-analysis tool as, e.g., the software package Digital Micrograph (Gatan) can be applied. First, a threshold in the gray values is set to select particles. As particles are connected, the threshold must be adjusted to identify single particles (cf. Fig. 1c). Since the further analysis counts slightly smaller particles, this discrepancy is measured and added to determined particle sizes. Finally, the results of the digital image analysis are filtered to rule out interconnected particles.

As imaginable, the size distribution of strongly interconnected particles cannot be analyzed directly with this routine. In this case, a possible way-out is a fit-by-eye of ellipses to discernible particles as shown in Fig. 2 for silica nanoparticles (batch SilNP010). The ellipses can be further processed with the above-mentioned automatic routine. Since the chosen ellipse areas are smaller than the real particles, after analysis a certain offset value has to be added to the measured size.

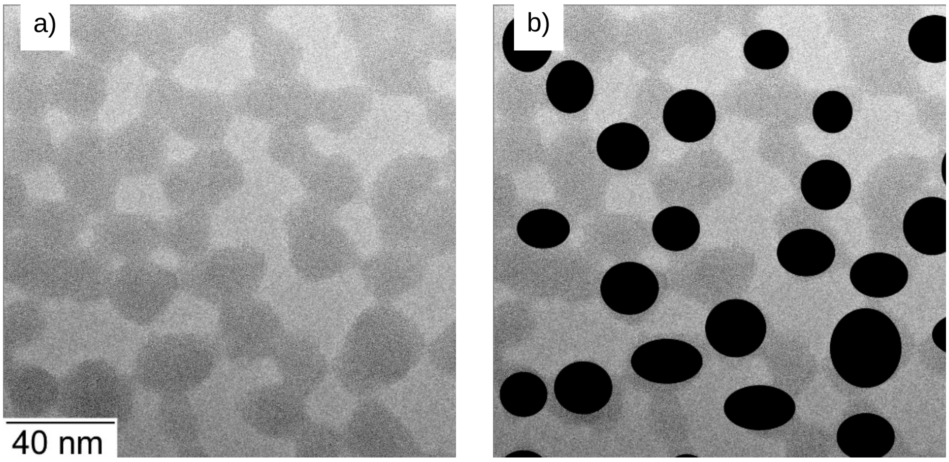

Fig. 2: Original TEM image of strongly interconnected silica nanoparticles (a), same image with drawn ellipses for further digital image analysis (b).

As evident from Fig. 2 the particles are only partly analyzed as the ellipses must not touch each other for the applied processing technique (i.e. Digital Micrograph). Also not all particles have an ellipsoid shape, so this procedure introduces some errors. To overcome this problem, another software tool was applied, as shown in Fig. 3. In Fig. 3a the particles have irregular shapes, so their contours were drawn free-hand. The situation is quite different for the CeO_2 nanoparticles shown in Fig. RS4b. In this projection, where the particles appear rhombus-like, their contours were marked with a polygon tool in the image analysis software (i.e. Adobe Photoshop). Afterwards the contours of all particles were analyzed automatically. The output of the routine can be varied widely, e.g. a circular diameter can calculated from the circumferences of the individual particles (Fig. 3a) or their length and width can be measured (Fig. 3). The advantage of this method is the analysis of the individual contours, so overlapping particles pose no difficulties.

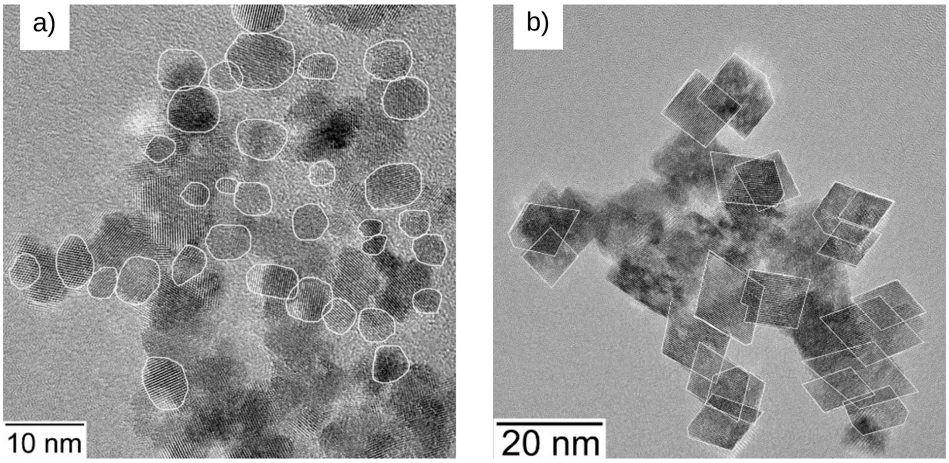

Fig. 3: HRTEM image of CeO_2 nanoparticles, (a) batch CeO_2NP_002 and (b) CeO_2NP_007. For determining the particle-size distribution either irregularly-shaped contours (a) or polygon-like ones (b) were chosen to mark individual nanoparticles and to measure their sizes.

References

[1] D.B. Williams, C.B. Carter, Transmission Electron Microscopy - A Textbook for Materials Science, Springer Science+Business Media, New York, 2009.

[2] Q. Zhang, J. Xie, Y. Yu, J. Yang, J.Y. Lee, Tuning the crystallinity of Au nanoparticles, small 6 (2010), 523-527.

[3] H. Hofmeister, Forty years study of fivefold twinned structures in small particles and thin films, Cryst. Res. Technol. 33 (1998), 3-25.

[4] K. Dub, F. Ernst, M.C. Pelsozy, J. Barthel, K. Tillmann, Expansion of interatomic distances in platinum catalyst nanoparticles, Acta Materialia 58 (2010) 836–845.

[5] B. Gamm, H. Blank, R. Popescu, R. Schneider, A. Beyer, A. Gölzhäuser, D. Gerthsen, Quantitative high-resolution transmission electron microscopy of single atoms, Microscopy and Microanalysis 18 (2012), 212–217.

[6] F.H. Herbstein, How precise are measurements of unit-cell dimensions from single crystals?, Acta Cryst. B56 (2000), 547-557.

Any further Information:

S1-6a Representative Electron Microscopy (TEM) picture(s)

<table>
<tr>
<td colspan="3">

Electron Microscopy and Focussed Ion Beam Nanostructuring

Technology:

- Scanning Electron Microscopy (SEM)
- Transmission Electron Microscopy (TEM)
- Focussed Ion Beam (FIB) Nanostructuring

Equipment:

- TEM FEI TITAN 80-300 with Cs image corrector, EDX spectrometer, monochromator, and high-resolution imaging energy filter
- Philips CM200 FEG/ST
- ZEISS922 Omega
- Field-emission (FE)-SEM FEI Quanta 650 ESEM
- FE-SEM ZEISS LEO 1530
- FIB FEI Strata 400

</td>
<td>

Category:
C. Particle Characterisation in- and ex-situ
Institute: Laboratory for Electron Microscopy (LEM), KIT

Location:
Karlsruhe Institute of Technology (KIT)
Campus South, Building 30.22
Engesserstr. 7
D-76131 Karlsruhe
Germany

Contact Details of Technology Expert:
Name: Prof. Dr. Dagmar Gerthsen
Phone: +49 721 608-43200
Fax: +49 721 608-43207
E-mail: dagmar.gerthsen@kit.edu

</td>
</tr>
<tr>
<td colspan="4">

Short technology description/Overview:

Different electron-microscopic techniques, including scanning electron microscopy (SEM), transmission electron microscopy (TEM) as well as scanning TEM (STEM), are applied to characterize the properties of nanoparticles and agglomerates thereof. Detailed information about the particle size and surface topography is obtained by field-emission SEM with a lateral resolution down to approximately 1 nm, whereas the crystal structure can be elucidated by electron diffraction in TEM and direct imaging at atomic resolution via high-resolution TEM (HRTEM). Both SEM and TEM investigations are combined with energy dispersive X-ray spectroscopy (EDXS) to reveal the chemical composition of the material in a quantitative manner, which can also be characterized along a line (line profiling) and two-dimensionally (X-ray mapping). An additional analytical tool for TEM inspection of particles at a high spatial resolution in the order of 1 nm is electron energy loss spectroscopy (EELS), which allows their quantitative element analysis, but also chemical-bond analyses by characterizing energy-loss near-edge structures (ELNES). Element distributions can be imaged through EELS line profiles and by energy-filtered TEM (EFTEM).

A target preparation of nanoparticle assemblies is facilitated by combined focused ion beam (FIB) milling and SEM imaging, allowing for example a stepwise sectioning of single particles. These experiments can be supplemented by in-situ EDXS analyses and low-kV HAADF (high-angle annular dark-field) STEM imaging, yielding strong atomic-number contrast.

</td>
</tr>
<tr>
<td colspan="4">

Main Features (Equipment Capabilities):

</td>
</tr>
<tr>
<td>

TEM/STEM FEI TITAN 80-300 cubed

- Operation modes and techniques (at 80 kV and 300 kV): TEM, Lorentz-mode TEM, aberration-corrected HRTEM, STEM, electron diffraction (SAED, CBED), EDXS, EELS/ EFTEM,

</td>
<td>

FE-SEM ZEISS LEO 1530 and FE SEM FEI Quanta 650 ESEM

- Accelerating voltages from 1 kV to 30 kV
- Secondary-electron (SE) imaging with optimum resolution of ~1 nm

</td>
<td colspan="2">

Dual-beam FIB FEI Strata 400

- Materials milling with Ga+ ions in the energy range from 1 to 30 kV – minimum structure size of ~10 nm
- Preparation and lift-out of TEM lamellae
- In-lens SE imaging (about 1 nm resolution with electron

</td>
</tr>
</table>

electron tomography, electron holography<br>• 0.08 nm information limit in HRTEM imaging<br>• 0.14 nm STEM resolution<br>• 0.7 eV energy resolution (FWHM of zero-loss peak) at 300 kV,<br>• ≤ 0.2 eV with monochromator<br>• Analytical double-tilt holder, ± 80° tomography holder	• Backscattered electron (BSE) imaging with optimum resolution of~ 2.5 nm<br>• EDXS analysis (detection of elements with Z ≥ 5)<br>• Low-vacuum operation up to 4000 Pa<br>• Liquid nitrogen cooled stage	excitation), < 1 nm resolution in HAADF STEM mode<br>• Material deposition (Pt, W) with ion or electron beam<br>• EDX spectrometer with silicon-drift detector (SDD), element detection for Z ≥ 5

Limitations / constrains

For investigations, powder material can be directly put on an electron-conducting support. For TEM/STEM characterization the particle size should be smaller than 200 nm. All samples have to be stable under high-vacuum conditions (except for SEM imaging with the FEI Quanta ESEM). Depending on the composition, the sample might be damaged by the electron beam (this holds especially organic material and polymers). EDXS allows detection of elements with atomic number Z ≥ 5 (EELS is able to detect Z ≥ 3).

Typical structures & designs

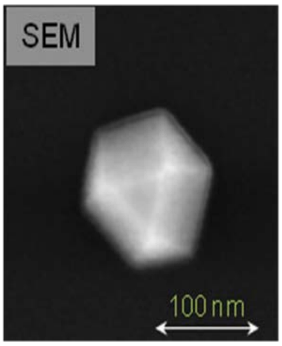

Secondary-electron image of a single Pt particle

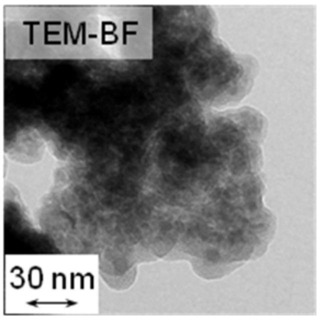

TEM bright-field image of a mixture of Fe2O3 nanoparticles in soot

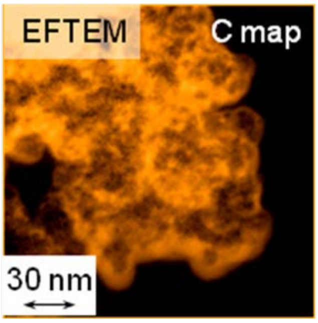

Carbon map of a Fe2O3 nanoparticles/ soot mixture obtained by EFTEM

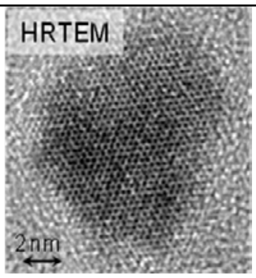

HRTEM image of an individual ZnO particle with about 10 nm size

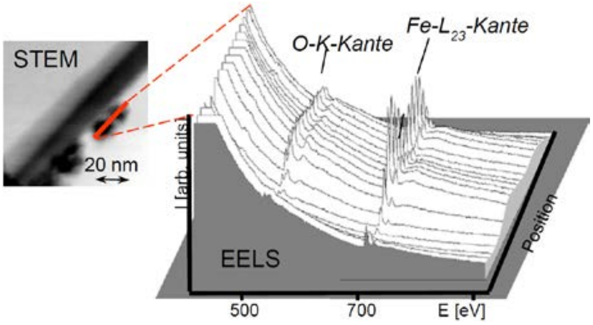

Series of EEL spectra across two single Fe_2O_3 particles embedded in soot

Any further Information:

S1-6b Representative Electron Microscopy (TEM)

Technology:<br>*Transmission Electron Microscopy (TEM)*<br><br>***Equipment:***<br>Gatan SC600 CCD camera<br>Oxford Instruments 80 mm2 X-max SDD EDX detector running INCA software	Category:<br>C. Particle Characterisation in- and ex-situ<br>Location: Univleeds<br>Contact Details of Technology Expert:<br>Name: Yunhong Jiang<br>E-mail: pedyj@leeds.ac.uk
FEI Tecnai F20 FEGTEM has been responsible for testing all batches of nanoparticles in this task. The equipment was operated at 200 kV fitted with Gatan SC600 CCD camera and Oxford Instruments 80 mm^2 X-max SDD EDX detector running INCA software. The microscope is operated in bright field mode with an extraction voltage of 3800 V and at spot size 3. The high resolution capabilities will obtain the information about atomic structure of the specimen. This TEM system is fully loaded including high angle annular dark field detector and X-ray energy disperse spectrometry. TEM samples of the NPs were prepared by nebulizing a thereof produced dispersion on a Cu grid (400 mesh) covered with a combined holey and ultrathin (about 3 nm) carbon film.<br><br>*TEM sample preparation:*<br><br>Sample preparation is very important for TEM observation and analysis. There are many ways available for preparing TEM sample. The facilities of TEM sample preparation has been focused on metallic materials, semiconductors, ceramics, and geo-science samples. The thickness of sample is between 10 and 200 nm depending on the material and the kind of observation. Samples are also to be electrically conductive, stable under vacuum and free of hydrocarbon contamination.	
Any further Information:	

S1-7 Particle size distribution of the aerosol

Scanning Mobility Particle Sizer (SMPS)	Category: C. Particle Characterization in and ex-situ<br>Institute: KIT<br>Location: Institute for Technical Chemistry (ITC)<br>Contact Details of Technology Expert:<br>Name: M.Sc. Sonja Mülhopt<br>Phone: ++49-721/608-2-3807<br>Fax: ++49-721/608-2-4303<br>E-mail: muelhopt@kit.edu

Short technology description/Overview:

As inhalation is one of the main uptake routes of nanoparticles into human bodies the particle size distribution of aerosolized NP is of interest. In Quality-Nano it is determined by Scanning Mobility Particle Sizer (SMPS) after suspending them in clean air.

Aerosolization

Depending on the kind of particle collective the corresponding method of aerosolization was chosen: bulk material of higher amounts was dispersed by rotating brush generator (RBG1000, Palas GmbH, Karlsruhe, Germany) whereas liquid suspensions were sprayed with the electrospray aerosol generator 3840 (TSI Inc., MN, USA). Nanoparticle powders of low amounts were suspended in liquid and also sprayed with the electrospray aerosol generator 3840.

Preparation of nanoparticle suspensions

From test particles delivered as powder a stock solution in pure water was prepared and sonified in the bath (USR 57, Merck EuroLab). This stock solution was suspended in 1 ml electro conductive solution, mostly in 5 mMol HNO_3.

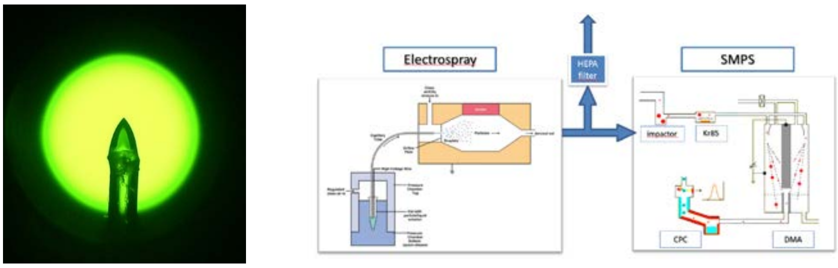

Figure 1: ***left***: view to the cone of the electrospray generator while aerosol generation; ***right***: the electrospray aerosol generator 3840 (TSI Inc., MN, USA) coupled with the scanning mobility particle sizer SMPS (TSI Inc., MN, USA)

Table 1: relevant specifications of the Model 3480 Electrospray Aerosol Generator

Mode of operation	Generation of aerosols from liquid solutions or suspensions using an electrospray method
Capillary dimension	25 µm, 250 mm length
Particle type	Solids or nonvolatile liquids soluble in 20 mM ammonium acetate solution in ultrapure water or 0.05% trifluoroacetic acid in ultrapure water. Suspensions in some solvents up to a particle size of 100 nm.
Initial droplet diameter	150 nm nominal
Particle generation rate	>10^7 particles/cm^3
Particle size range	2 to 100 nm
Liquid conductivity	0.2 S/m nominal

Measuring the number size distribution

Number size distributions were measured using a scanning mobility particle sizer (SMPS, DMA 3071 with CPC 3775, TSI). Aerosols typically show a log-normal distribution of size. The number size distribution was measured at least three times. The data were corrected regarding sampling losses using the penetration factor for tubing according to Soderholm [34] and analyzed by determining the mean +- standard deviation of the three number measurements in each size channel. The resulting particle number size distribution dN/dlog(d_P) [1/cm³] can be characterized by the three parameters of the log-normal fits: the modal value x_m [nm], the geometric standard deviation σ_{geo} [-/-] and the total number concentration c_N [1/cm³]. These three parameters are reported in the data sheets.

Typical Samples & Images:

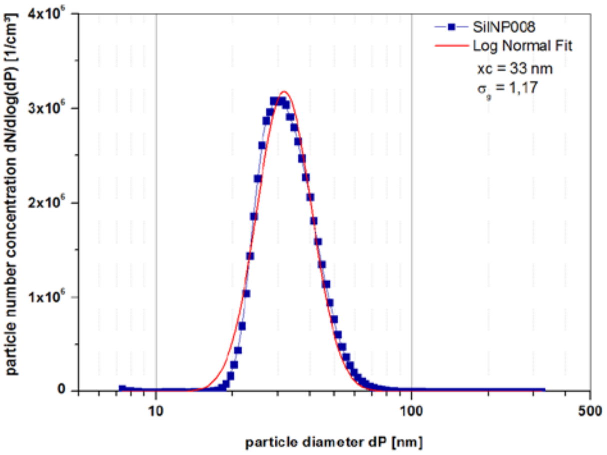

Any further Information:

S1-8a Particle size distribution (DLS)

Equipment Name:<br><br>*Malvern Zetasizer Nano ZS*	Category: C. Particle Characterization<br>Institute: Campus UCD<br>Contact Details of Technology Expert:<br>eugene.mahon@cbni.ucd.ie

Overview

Dynamic Light Scattering which can measure suspended particle hydrodynamic radius across a large range.

Sample Protocol: Stöber Silica Aqueous Suspension DLS Size Measurement Protocol

(modified from protocol developed by Dr. Sonia Ramirez)

1. Wear appropriate personal protective gear and take appropriate precautions when handling nanomaterials.
2. Power up instrument at least 30 min in advance of measurements.
3. Suspend particle sample to the required concentration in MilliQ water.
4. Take the required volume of particle solution to fill the DLS cuvette to the recommended mark. Quartz or optical-quality glass cuvettes are generally preferred, but good quality plastic cuvettes may be substituted. All cuvettes, but in particular the more scratch-prone plastic cuvettes, should be routinely inspected prior to use and discarded if surface scratches or defects are visible.
5. Pre-rinse clean cuvette with filtered deionised water at least 3 times prior to loading sample. This is best done in a clean bench if available. Disposable plastic cuvettes should also be rinsed to eliminate any dust that might have deposited on them.
6. Recommendations for loading cuvette prior to analysis:
   6.1. Load sample into cuvette using minimum volume necessary to ensure liquid level is at least 2 mm above the entrance height of the laser beam for your particular instrument configuration (or refer to the instrument manual for recommended filling height for your cell and instrument configuration). ***Do not overfill cuvettes past the recommended level, as overfilling can lead to thermal gradients that will adversely impact measurement accuracy.*** For microcuvettes with a sample well insert, fill the well with sample, but do not fill beyond the well lip.
   6.2. Take care not to touch the cuvette windows with your bare hands while loading. Wipe the outside of glass or quartz cuvettes with lens paper to ensure cleanliness. If using disposable plastic cuvettes, do not wipe outside surface as this may leave scratches that will interfere with measurements.
   6.3. Cap the cuvette to prevent dust contamination and evaporation of solution.
   6.4. Inspect the cuvette to insure that air bubbles are not attached to the optical window area. If necessary, ***gently*** tap cuvette on a non-metallic padded surface to release bubbles before placing the cuvette in the sample holder, or, if necessary, repeat the loading procedure with fresh sample. ***Never shake cuvette, as this may introduce air bubbles or entrap air in the sample well of some microcuvettes.***
   6.5. Place the cuvette correctly in the sample holder; i.e. optical windows should be facing the incident beam and detector.
7. The recommended measurement temperature (temperature of the test material) is near to or slightly above laboratory temperature. Measurements should not be conducted outside the range (20 to 25.5) ºC. Recommended refractive index and viscosity values for the suspending medium (H2O) used in the calculation of z-average size from DLS measurements are given in table 1. If your instrument permits accurate temperature control, perform the size measurement at 25°C.

Refractive Index: 1.332 (suitable for wavelengths in the 488-750 nm range within the temperature range of 20 to 25.5°C)

If your instrument does not allow or require user input of viscosity and refractive index values, use the default settings for pure water at the measurement temperature, and report these values with your data.

Table 1. Viscosity values used in the calculation of z-average size from DLS when water is used as suspending medium

Temperature T [°C]	Absolute Viscosity [mPa·s]	Temperature T [°C]	Absolute Viscosity [mPa·s]
20	1.002	20.5	0.989
21	0.977	21.5	0.966
22	0.954	22.5	0.943
23	0.932	23.5	0.921
24	0.910	24.5	0.900
25	0.890	25.5	0.880

Other values can be interpolated using the above data.

8. Allow 4 min equilibration time at the measurement temperature (25oC if possible) if the volume of sample used is 1mL or less. Larger sample volumes may require longer equilibration times. The temperature should be controlled and measured with a precision of 0.1 °C. Accuracy of the temperature measurement device should be better than 0.5 °C.
9. Take 3 different aliquots from the diluted sample (as per point 3) and make 3 independent measurements or repeats. This should be done for each received material. This will allow us to establish measurement repeatability. Measurement duration should be set according to instrument manufacturer's recommendations.4
10. If scattering intensity exceeds the optimum count rate for your instrument as stipulated by the manufacturer, the test materials can be diluted. To perform the dilutions use deionised water (18m resistance) filtered to at least 0.2 µm or smaller.
11. If contamination or dust particles appear to be present in the test material (e.g., based upon high data variability or the occurrence of large size modes)
    11.1. Change the cuvette and use fresh sample. Repeat steps 6-10
    11.2. If the problem is not solved, the test materials can be filtered prior to analysis using a syringe filter in the range 0.2 – 0.45 µm pore size.

Main Features (Equipment Capabilities):

- Size measurement from 1nm (diameter) to 10 microns using patented *NIBS* (Non-Invasive Back Scatter) technology
- Zeta potential of proteins and particles from 3.8 nm up to 100 microns (diameter) using patented *M3-PALS* technology
- Sample concentrations from 0.1ppm to 40%w/v

Typical Samples & Images:

DLS distribution (bottom left) of fluorescent Stöber silica with for comparison TEM and DCS (Differential Centrifugal Sedimentation) results.

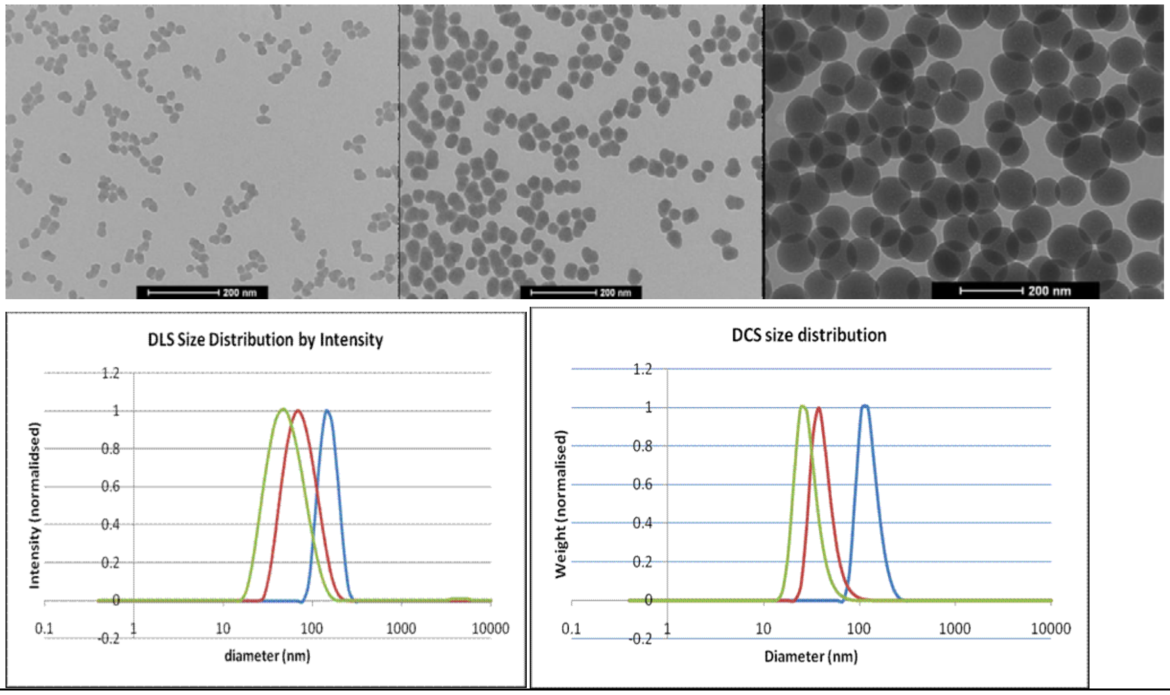

Any further Information:

S1-8b Particle size distribution (DLS)

<table>
<tr><td>Technology:
Dynamic Light Scattering (DLS)</td><td>Category:
C. Particle Characterisation in- and ex-situ
Location:
Univleeds
Contact Details of Technology Expert:
Name: Yunhong Jiang
E-mail: pedyj@leeds.ac.uk</td></tr>
<tr><td colspan="2">Short technology description / Overview:

Dynamic Light Scattering (DLS) is an important tool for characterizing the size of nanoparticles in solution. DLS measures the light scattered from a laser that passes through a colloidal solution and by analyzing the modulation of the scattered light intensity as a function of time, the hydrodynamic size of particles and particle agglomerates can be determined. DLS is a valuable tool for determining and measuring the agglomeration state of nanoparticles as a function of time or suspending solution. When DLS sizing data is compared to Transmission Electron Microscopy images, the aggregation state of the particles can be determined. In an unagglomerated suspension, the DLS measured diameter will be similar or slightly larger than the TEM size. If the particles are agglomerated, the DLS measurement is often much larger than the TEM size and can have a high polydispersity index (large variability in the particle size).
The Malvern Zetasizer series combines a particle size analyzer, zeta potential analyzer and molecular weight analyzer for particles and molecules from below a nanometer in size to several microns. The systems measure size using dynamic light scattering. Zeta potential and electrophoretic mobility use electrophoretic light scattering, and molecular weight use static light scattering. For particles in situ in biofluids (e.g. cell culture media), additional care is needed in performing the experiments and in interpreting the data, especially given the known relationship between size and scattering (i.e. a few large agglomerates can mask the presence of a large number of small particles).

DLS sample preparation:

A concentration should be chosen such that the result is independent of the concentration chosen. The maximum concentration should be less than 1% by volume to avoid particle interaction. The minimum concentration should generate a minimum count rate of 10,000 counts per second (10kcps) for water as the dispersant. The recommended concentration is in the range of 0.1% (for particles as small as a few nm) to 0.0001% (for particles as large as 1 μm) by volume. Ultrasonication was used to remove air bubbles or to breakup agglomerates. Keep in mind that in some cases the primary particles may be damaged. The size range is from 1 nm up to 6 μm, based on 1 g/cm^3 density.</td></tr>
<tr><td colspan="2">Any further Information:</td></tr>
</table>

S1-9 Gas sorption analysis

Gas sorption analysis	Category:<br>C. Particle Characterisation in and ex-situ<br>Institute: UU<br>Location: Ångström lab<br>Contact Details of Technology Expert:<br>Johan Forsgren johan.forsgren@angstrom.uu.se

Short technology description / Overview

Gas sorption analysis enables measurement of specific surface area, pore volume and pore size distribution of dry particles. The technique is based on the gradual multilayer formation of physisorbed gas molecules on solid surfaces at different pressures. An isotherm is obtained by measuring the amount of adsorbed gas molecules on the sample at different pressures, and the data is used for calculations of specific surface area, pore volume and pore size distribution. The adsorption branch in the isotherm is used to determine the specific surface area according to the BET-equation and information about the pores in the powder can for instance be obtained by applying the BJH-equation to the desorption branch. The BJH-equation is a modified version of the Kelvin equation describing desorption of molecules from condensed gas contained in small pores. Another way to determine the porous properties of a powder is to use density functional theory (DFT) on the isotherm, which is a more modern and accurate method. The nature of the technique only allows for analysis of pores in the meso to micro size range as the limited size of these pores makes the gas condense inside the pores below the saturation pressure (capillary condensation), and it is this phenomenon that enables analysis of the pores in the sample.

For accurate measurement, the sample needs to be degassed properly in order to remove all water from the surface prior to analysis, preferably over 110°C. Between 0.1g and 0.5g of sample is generally a good quantity for analysis.

The analysis is carried out using an ASAP 2020 (Micromeritics) at 77K (-196°C) with N_2 as analysis gas.

Main Features (Equipment Capabilities):

- Specific surface area
- Pore volume
- Pore size distribution

Typical Samples & Images:

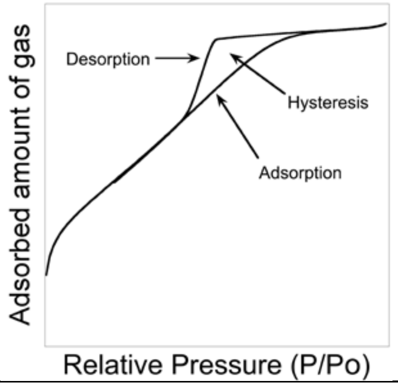

Any further Information:

S1--10 Zeta potential (surface charge)

Zeta potential	Category:<br>C. Particle Characterisation in and ex-situ<br>Institute: UU, Location: Ångström lab<br>Contact Details of Technology Expert:<br>Johan Forsgren **johan.forsgren@angstrom.uu.se**

Short technology description / Overview

The zeta potential describes the electrical potential at the slipping plane in the electrical double layer of dispersed particles, i.e. the potential between the dispersion medium and the stationary layer of fluid surrounding the particles. The magnitude of the zeta potential relates to the surface charge of the particles and the pH of the dispersion medium. The ions in the dispersion medium (and hence the pH) affect the nature of the electrical double layer around the particles where a positive charge is built up under acidic conditions and vice versa. Therefore is pointless to measure the zeta potential without knowing the pH of the suspension. The pH value where the zeta potential is zero is called the isoelectric point (IEP) and is the point where the stability of the colloidal suspension is lowest. A colloidal suspension is generally considered stabile at zeta potentials greater than ±30mV. At low zeta potential, the electrostatic repulsion between the particles in suspension is low and the particles tend to flocculate.

The zeta potential does not only serve as a measure of the stability of the colloidal suspension of particles, but can also be used as an indicator of the surface charge of the particles as there is no direct way to measure this property. The zeta potential is measured using electrophoresis where particles in suspension are subjected to an electric field and the velocity of the moving particles is measured using light scattering techniques. From the velocity of the particles, the zeta potential can be obtained using the Henry equation.

The zeta potential is preferably measured in aqueous electrolytes containing 10^{-2}-10^{-3} M salt. For example, the particles can be suspended in an 10^{-3} M KCl electrolyte where the pH is adjusted with 10^{-2} M solutions of HCl and NaOH.

Main Features (Equipment Capabilities):

- Measurement of zeta potential of particles in suspension at different pH
- Assessment of colloidal suspension stability
- Determination of isoelectric point of particles

Typical Samples & Images:

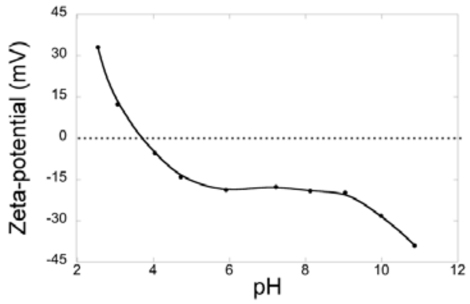

Any further Information:

S1-11 Photocatalytic activity

Equipment Name:<br><br>*Photocatalytic Activity test*	Category:<br>C. Particle Characterisation in and ex-situ<br>Institute: Institut Català de Nanotecnologia (ICN)<br>Location: Campus UAB<br>Contact Details of Technology Expert:<br>Jordi Piella<br>Tel. +34-937374624<br>Jordi.piella@icn.cat

Short technology description/Overview

The principle of the photo catalysis by nanoparticles is straightforward. Under a radiation source, and upon adsorption of photons with energy higher than the band gap, electrons are excited from the valence band to the conduction band, creating electron-hole pairs. These charge carriers, in particular the electrons from the conduction band, migrate to the surface of the particle -where the reactions take place- and facilitate reduction reactions. The capture of holes and electrons by adsorbed species generate a variety of highly reactive radicals and intermediate species such as OH·, O2-, H2O2 capable of producing the oxidation of organic matter. In an aqueous media, the kinetic of the photocatalytic reactions mediated by nanoparticles can be followed spectrophotometrically by studying the degradation of organic dyes such as Rhodamine B (RhB), Methylene Blue or some phenol derivatives during photo-oxidation. Thus, by testing the photocatalytic degradation of RhB as a model contaminant it is possible to have an approximation of the potential environmental applications for the removal of contaminants from wastewater.

Material

Rhodamine B (RhB)

Procedure

Photocatalytic activity test is performed in Milli-Q® water at ambient temperature (25ºC) and circumneutral pH as a function of the decolorization of RhB with time.

An aqueous solution of tested particles is stirred with RhB dye (1 mM). If the sample is in powder form, the solution is previously sonicated for 15 minutes to properly disperse the particles before RhB addition. The solution is kept in dark conditions and allowed to reach the absorption-desorption equilibrium among the photocatalyst for 30 min. UV Lamp (100 W, 365 nm, Ted Pella, Inc.) is used as a light source to trigger the photocatalytic reaction. The average light intensity striking on the surface of the reaction solution is around 8.9 mW/cm^2. After UV irradiation for some time, 1 mL of the sample is taken and the absorbance of RhB remaining in the supernatant is measured using a UV-Visible spectrophotometer (Shimadzu UV-2401). In the case that the particles absorb in the same range as RhB, centrifugation of the solution at high speed is done prior to the measurements.

Main Features (Equipment capabilities):

UV-Vis spectrophotometer (Shimadzu UV-2401) for absorbance measurement form 300 nm to 800 nm.

Typical Samples & Images:

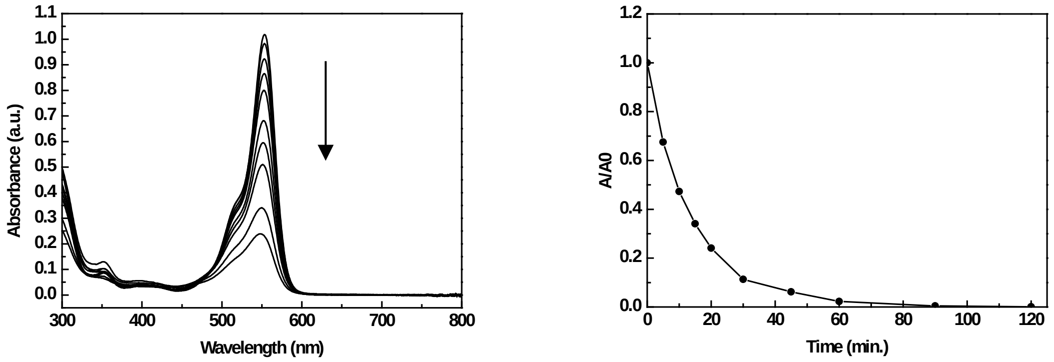

Figure 1: Photocatalytic degradation of rhodamine B in the presence of commercial Degussa P25 nanoparticles (Sigma-Aldrich) under UV light irradiation (left) Temporal Evolution of UV–Vis spectrum of RhB, (right) Photocatalytic performance of RhB.

Kinetic of the reaction

$$\frac{A}{A_0} = \exp(-kt)$$

A = absorbance

K = kinetic constant

t = time

Any further Information:

Beydoun, D.; Amal, R.; Low, G.; McEvoy, S. Role of nanoparticles in photocatalysis. Journal of Nanoparticle Research 1999, 1, 439-458.

S1--12 Octanol-Water partition coefficient Pow

Equipment Name:<br><br>*Octanol-Water partition coefficient*	Category:<br>C. Particle Characterisation in and ex-situ<br>Institute: Institut Català de Nanotecnologia (ICN)<br>Location: Campus UAB<br>Contact Details of Technology Expert:<br>Jordi Piella<br>Tel. +34-937374624 Jordi.piella@icn.cat

Short technology description

The partition coefficient (P) is defined as the ratio of the equilibrium concentrations of a dissolved substance in a two-phase system consisting of two largely immiscible solvents. In the case of n-octanol and water:

$$P_{ow} = \frac{C_{n-octanol}}{C_{water}}$$

The partition coefficient, being the quotient of two concentrations or the quotient of the fractions of the test substance in the two phases multiplied by a fixed volume ratio, is dimensionless and is usually given in the form of its logarithm to base ten.

Octanol-water partition coefficient can be measured in two different ways: regarding the concentration of ions from the nanoparticles or in terms of the number of nanoparticles in each phase. The last one is the one presented here. However, in this case the results become highly dependent on the type of coating on the surface of the particles and thus it partially loses its sense. Moreover, particles synthetized in aqueous solution are obvious to be more affine to water than octanol.

Material

1-Octanol (99%) was purchased from Sigma-Aldrich. Milli-Q® water is used in all the experiments. Water taken directly from an ion exchange should not be used.

Procedure

Before a partition coefficient is determined, the two solvents are mutually saturated at the temperature of the experiment. To do this, it is practical to shake two large stock bottles, one containing n-octanol and a sufficient quantity of water, and the other containing water and a sufficient quantity of n-octanol, for 24 hours on a mechanical shaker, and then to let them stand long enough to allow the phases to separate.

Equal volume of dispersed nanoparticles in water and n-octanol are mixed for 12 hours at 25°C. Then the two phases are allowed to separate for 2 hours and the concentration of nanoparticles in the n-octanol phase are analysed by Inductively Coupled Plasma Mass Spectrometry (ICP-MS) Agilent instrument (Model: 7500cx) with a detection limit of 0.02386 ppb . In case that the nanoparticles are in powder form, the sample is sonicated (Brandson 2510) for 15 min for better dispersion. Since ICP-MS cannot be used for polystyrene nanoparticles, the presence of these particles in the octanol phase is measured by comparing the absorbance of the n-octanol phase before and after the experiment.

Main Features (Equipment capabilities):

Inductively Coupled Plasma Mass Spectrometry (ICP-MS) Agilent instrument (Model: 7500cx) with a detection limit of 0.02386 ppb.

Typical Samples & Images:

S1-13 Radical Formation Potential

Test for reactive oxygen species by DCF (DCF test):	Category: C. Particle Characterization in and ex-situ<br>Institute: KIT<br>Location: Eggenstein-Leopoldshafen, Campus North<br>Contact Details of Technology Expert:<br>Name: Dr. Silvia Diabate<br>Phone: ++49-721/608-2-2692<br>E-mail: silvia.diabate@kit.edu

Material

- 5 mM 2′,7′-dichlorodihydrofluorescein-diacetate ($DCFH_2$-DA, Invitrogen, Karlsruhe, Germany) in ethanol, stored in aliquots at -20°C
- 0.01 N sodium hydroxide (NaOH) solution, prepared from NaOH pellets (Merck, Darmstadt, Germany).
- Phosphate buffered saline (PBS) without Ca^{2+}, without Mg^{2+}, pH 7.4 (Invitrogen, Karlsruhe, Germany)
- Peroxidase from horseradish (HRP, Sigma, Taufkirchen, Germany)
- 30% hydrogen peroxide (H_2O_2, Sigma, Taufkirchen, Germany)

Test procedure

The test uses the oxidation of the non-fluorescent 2′,7′-dichlorodihydrofluorescein ($DCFH_2$) to the fluorescent 2′,7′-dichlorofluorescein (DCF) as an indicator for the presence of reactive oxygen species. Therefore, the commercial substance $DCFH_2$-DA must be deacetylated with NaOH. 0.1 ml of 5 mM $DCFH_2$-DA in ethanol is mixed with 2.4 mL of 0.01 N NaOH and incubated at room temperature (24°C) for 30 min. For neutralization, 10 ml PBS is added and kept on ice in the dark until use. Just prior to use, HRP is added as a catalyst (2.2 U/ml). The $DCFH_2$ concentration in the working solution is 40 µM.

Suspensions of test particles are sonified for 10 min and different concentrations are prepared in PBS. H_2O_2 standard preparations (0.04 to 10 µ*M*) are prepared as well. The test solutions are mixed 1:1 (v/v) with the prepared $DCFH_2$ solution and incubated at 37°C for 15 min in the dark. The solution is then centrifuged (20,000 × g for 15 min) to remove the particles and the fluorescence of the supernatant is monitored at 485 nm excitation and 530 nm emission using a fluorescence microplate reader (e.g. BIO-TEK FL600 from MWG-Biotech AG, Ebersberg, Germany). Solutions containing polystyrene nanoparticles were centrifuged for 1 h. Results are expressed as fold changes of the particle free sample.

Main Features (Equipment Capabilities):

Fluorimeter, 485 nm excitation and 530 nm emission

References

Cathcart, R., Schwiers, E., and Ames, B.N. (1983). Detection of picomole levels of hydroperoxides using a fluorescent dichlorofluorescein assay. Anal. Biochem. 134, 111-116.

Foucaud, L., Wilson, M.R., Brown, D.M., and Stone, V. (2007). Measurement of reactive species production by nanoparticles prepared in biologically relevant media. Toxicol. Lett. 174, 1-9.

Any further Information:

about 100 mg of particles are necessary for the test

Supplemental S2: Physicochemical characterization data sheets of silica NMs from Stöber synthesis

**REPRESENTATIVE TEST PARTICLES**<br>Nanomaterial Identification<br>according to OECD<br>ENV/JM/MONO(2009)20/REV pp29.	Research Infrastructure QualityNano
Nanomaterial name — Silica-NP<br>Particle Code — SilNP_002<br>Composition — SiO_2<br>Method of production — Stöber-Synthesis	**Manufacturer /Institute:**<br>**Eugene Mahon , provided by UCD**<br>**Technology Expert:** Eugene Mahon
**Kind of suspension:**	**Suspension** ☒ **Powder** ☐<br>Suspended in: Pure water<br>pH: ??<br>stabilizer: none

**Property**		**unit**	**Method / Institute**	**Value**
Agglomeration/aggregation			HRTEM (KIT)	Aggregated
Crystalline phase			D5000 Diffractometer /UU<br>PANalytical X´Pert diffractometer /ICN	Amorphous
Crystallite size			HRTEM (KIT)	n.a.
Octanol-water partition coefficient			Extraction/ ICP-MS (ICN)	P_{OW}=0
Photocatalytic activity			Rhodamin-B Bleaching/UV-B-100<br>10mg/ml in Water (ICN)	10% / 10 min
Porosity		-/- or %	n.a.	n.a.
Pour density		cm3/g	(BJH) Pore Size Distribution and Volume by ASAP2020 (UU)	n.a.
Redox potential				
Size distribution: (suspension in water)	Modal value X_M (PDI)	nm	Zetasizer Nano ZS /UU<br>Zetasizer Nano ZS /UCD	82.7±0.35 (PDI: 0.049)<br>87.4(PDI:0.057)/NM: 67.5
	Total concentration	mg/ml		0.1 – 2 mg/ml
Size distribution: (Aerosol)	Modal value X_M (σ_{geo})	nm	SMPS TSI, DMA 3071 with CPC 3022A / KIT<br>dispersed by AGF2.0	40 nm (soluble comp.?)<br>70 nm (σ_{geo}=1.42)
	Total concentration	mg/cm³ or #/cm³		1 x 10^5 /cm^3
Solubility in _____		g/l		
Solubility in H_2O		g/l	Extraction/ ICP-MS (ICN)	30.8±2.1
Specific surface area		cm²/g	BET by ASAP2020 (UU)	n.a.
Surface chemistry				
Zeta potential (surface charge)		eV	Zetasizer Nano ZS /UU	-51.7 ± 1.2eV (pH 9.23)

<table>
<tr><td>Summary of trace analyses</td><td></td><td>ICP-MS / KIT</td><td></td></tr>
<tr><td>Ni</td><td>µg/g</td><td></td><td><0.5</td></tr>
<tr><td>Cu</td><td>µg/g</td><td></td><td>1.0</td></tr>
<tr><td>Electron microscopy
Method / technical data of microscope:
SOP: See KIT (LEM)

Magnification: 450.000

FEI Tecna i 120
SOP: see UCD</td><td colspan="3">Electron Microscopy by KIT (LEM)
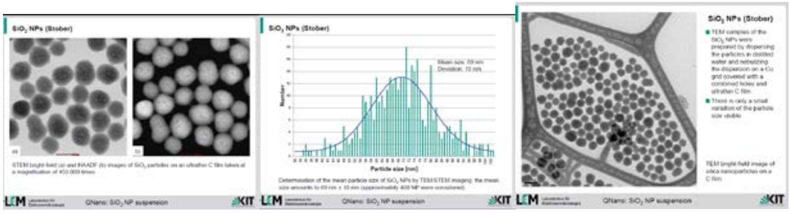
Mean Diameter : 69 nm

Electron Microscopy by UCD
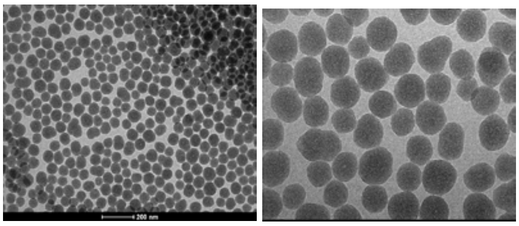
Av. Diameter (ImageJ,200 particles) = 68nm</td></tr>
<tr><td>Radical formation potential by DCF-Test:
(H_2O_2 as positive control) (KIT)</td><td colspan="3">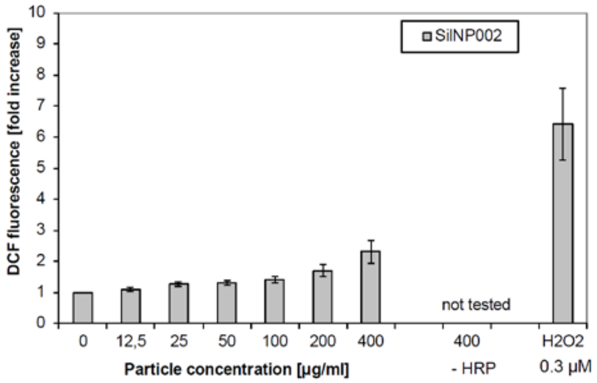

Result: SiINP_002 NPs induced a very low increase of DCFH oxidation at high concentrations indicating a negligible potential to produce reactive oxygen species.</td></tr>
<tr><td colspan="4">Comments and other relevant physical-chemical properties and material characterization information</td></tr>
<tr><td colspan="2">Place, Date Karlsruhe, 18.03.2013</td><td colspan="2">Responsible H.-R. Paur, KIT</td></tr>
</table>

**REPRESENTATIVE TEST PARTICLES**<br>Nanomaterial Identification<br>according to OECD<br>ENV/JM/MONO(2009)20/REV pp29.	Research Infrastructure QualityNano
Nanomaterial name: Silica-NP<br>Particle Code: SilNP_003<br>Composition: SiO_2<br>Method of production: Stöber-Synthesis	**Manufacturer /Institute/Date**<br>**Eugene Mahon ; provided by UCD; 10/2011**<br>**Technology Expert:** Eugene Mahon
**Kind of suspension:**	**Suspension** ☒ **Powder** ☐<br>Suspended in: Pure water<br>pH: n.d<br>stabilizer: none

**Property**		**unit**	**Method / Institute**	**Value**
Agglomeration/aggregation			HRTEM (KIT)	Agglomerated
Crystalline phase			D5000 Diffractometer (UU)<br>PANalytical X´Pert diffractometer (ICN)	Amorphous
Crystallite size			HRTEM (KIT)	n.a.
Octanol-water partition coefficient			Extraction/ ICP-MS (ICN)	n.a.
Photocatalytic activity			Rhodamin-B Bleaching/UV-B-100<br>10mg/ml in Water (ICN)	medium
Porosity		-/- or %	n.a.	n.a.
Pour density		cm3/g	(BJH) Pore Size Distribution and Volume by ASAP2020 (UU)	n.a.
Size distribution:<br>(suspension in water)	Modal value X_M (PDI)	nm	Zetasizer Nano ZS /UCD<br>DCS (UCD) (RWtAv / RNumAv)	129.3 (PDI:0.017)/ NM: 109.4<br>102.7/99.8
	Total concentration	mg/ml		1mg/ml
Size distribution:<br>(Aerosol)	Modal value X_M (σ_{geo})	nm	SMPS TSI, DMA 3071 with CPC 3022A / KIT<br>dispersed by electrospray	118 (σ_{geo}=1.12)
	Total concentration	mg/cm³ or #/cm³		7.31x 10^3/cm^3
Solubility in _____		g/l		
Solubility in H_2O		g/l	Extraction/ ICP-MS (ICN)	392.2 g/L
Specific surface area		cm²/g	BET by ASAP2020 (UU)	n.a.
Surface chemistry				
Zeta potential (surface charge)		eV	Zetasizer Nano ZS /UU	-60.9±10.2eV<br>(pH 8.74)
Summary of trace analyses			ICP-MS / KIT	
B		µg/g		22.0
Na		µg/g		36.0
K		µg/g		12.4
Ca		µg/g		36.0

<table>
<tr><td>Electron microscopy
Method / technical data of microscope:
SOP: See KIT (LEM)

magnification: 450.000</td><td>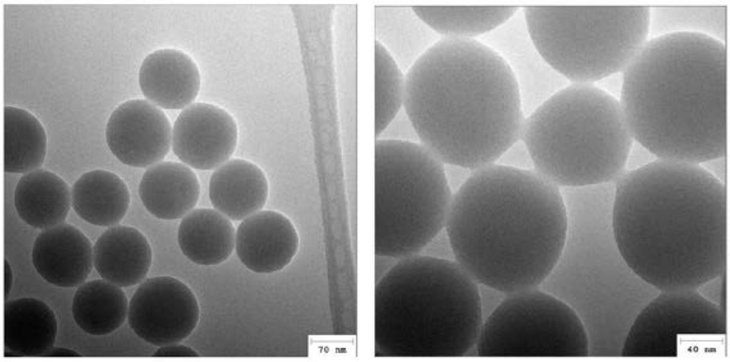

Electron Microscopy by KIT (LEM); Diameter : 70-120 nm</td></tr>
<tr><td>FEI Tecna i 120
SOP: see UCD</td><td>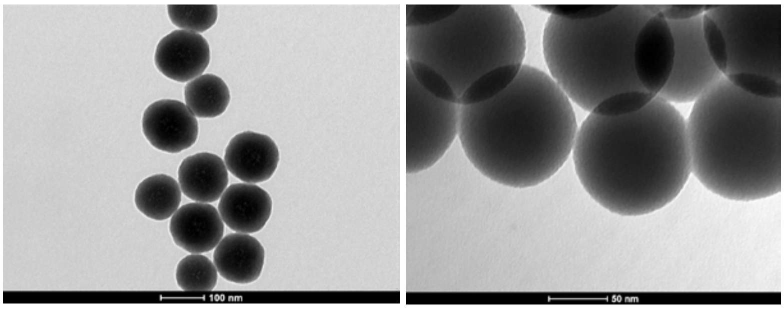

Electron Microscopy by UCD
Av. Diameter (ImageJ, XX particles) = 122nm</td></tr>
<tr><td>Radical formation potential by DCF-Test:
(H_2O_2 as positive control) (KIT)</td><td>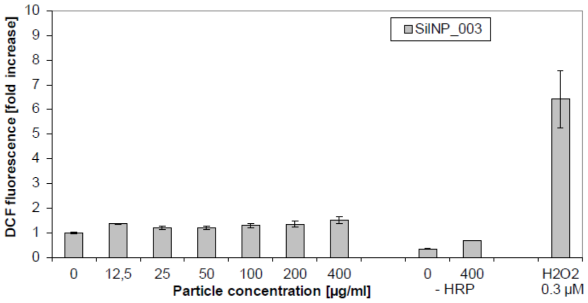

Result: SilNP_003 NPs induced no significant DCFH oxidation indicating no potential to produce reactive oxygen species.</td></tr>
<tr><td>SilNP_003_Corona Fingerprint_UCD</td><td>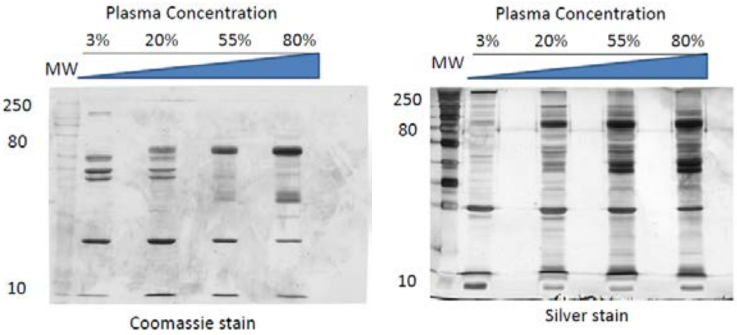
</td></tr>
<tr><td colspan="2">Comments and other relevant physical-chemical properties and material characterization information</td></tr>
</table>

<table>
<tr><td>Place, Date Karlsruhe, 29.11.2016</td><td>Responsible H.-R. Paur, KIT</td></tr>
</table>

REPRESENTATIVE TEST PARTICLES
Nanomaterial Identification
according to OECD
ENV/JM/MONO(2009)20/REV pp29.

Research Infrastructure QualityNano

Nanomaterial name	Silica-NP	**Manufacturer /Institute/Date**
Particle Code	SilNP_004	**Eugene Mahon ; provided by UCD; 10/2011**
Composition	SiO_2	**Technology Expert:** Eugene Mahon
Method of production	Stöber-Synthesis	

Kind of suspension:

**Suspension**	☒	**Powder**	☐
Suspended in	Pure water		
pH	n.d.__________		
stabilizer	none________		

**Property**		**unit**	**Method / Institute**	**Value**
Agglomeration/aggregation			HRTEM (KIT)	Agglomerated
Crystalline phase			D5000 Diffractometer (UU)<br>PANalytical X´Pert diffractometer (ICN)	Amorphous
Crystallite size			HRTEM (KIT)	n.a.
Octanol-water partition coefficient			Extraction/ ICP-MS (ICN)	
Photocatalytic activity			Rhodamin-B Bleaching/UV-B-100 10mg/ml in Water (ICN)	none
Porosity		-/- or %	n.a.	n.a.
Pour density		cm3/g	(BJH) Pore Size Distribution and Volume by ASAP2020 (UU)	n.a.
Size distribution: (suspension in water)	Modal value X_M (PDI)	nm	Zetasizer Nano ZS /UCD<br>DCS (UCD) (RWtAv / RNumAv)	75.15 (PDI:0.042) NM: 59.9<br>61.9/56.9
	Total concentration	mg/ml		1mg/ml
Size distribution: (Aerosol)	Modal value X_M (σ_{geo})	nm	SMPS TSI, DMA 3071 with CPC 3022A / KIT<br>dispersed by electrospray	65 (σ_{geo}=1.125)
	Total concentration	mg/cm³ or #/cm³		1.04x 10^4/cm^3
Solubility in _____		g/l		
Solubility in H_2O		g/l	Extraction/ ICP-MS (ICN)	298.0 g/L
Specific surface area		cm²/g	BET by ASAP2020 (UU)	n.a.
Surface chemistry				
Zeta potential (surface charge)		eV	Zetasizer Nano ZS /UU	-54.9 ± 14.0eV (pH 8.93)
Summary of trace analyses			ICP-MS / KIT	
B		µg/g		14.8
Na		µg/g		30.0
Mg		µg/g		10.0
Ca		µg/g		46.0
Zn		µg/g		0.8

**Electron microscopy**<br>Method / technical data of microscope:<br>SOP: See KIT (LEM)<br><br>Magnification: 450.000	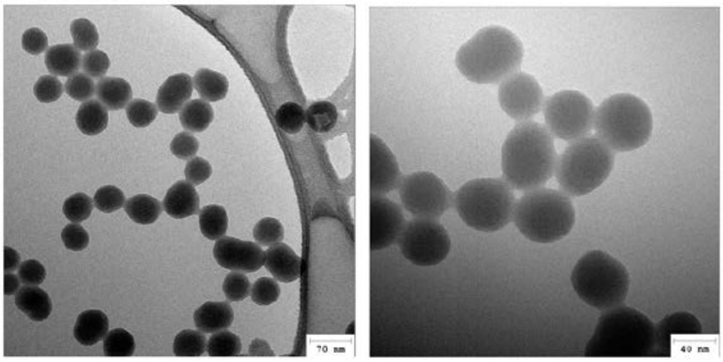<br><br>Electron Microscopy by KIT (LEM); Diameter : 40-70 nm
FEI Tecna i 120<br>SOP: see UCD	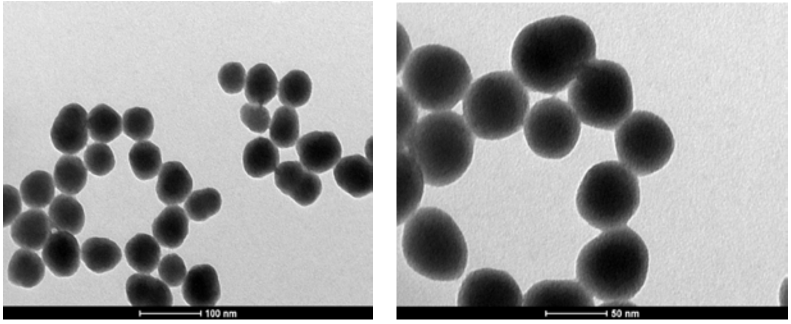<br><br>Electron Microscopy by UCD<br><br>Av. Diameter (ImageJ) = 68.45nm
Radical formation potential by DCF-Test:<br>(H_2O_2 as positive control) (KIT)	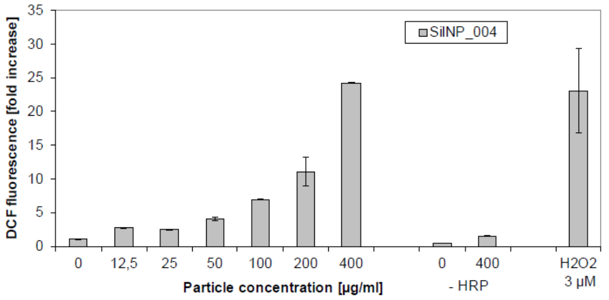<br><br>Result: SilNP_04 NPs induced a significant DCFH oxidation at high concentrations indicating a potential to produce reactive oxygen species.
SilNP_004_Corona Fingerprint_UCD	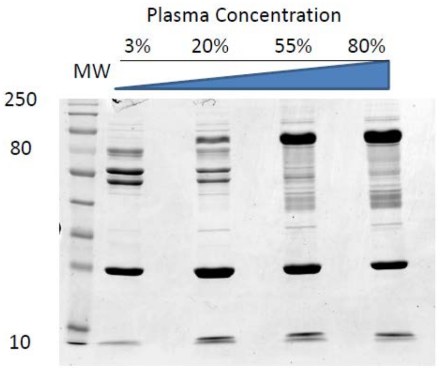

Comments and other relevant physical-chemical properties and material characterization information

**Place, Date Karlsruhe, 28.02.2013**	**Responsible H.-R. Paur, KIT**

**REPRESENTATIVE TEST PARTICLES**<br>Nanomaterial Identification<br>according to OECD<br>ENV/JM/MONO(2009)20/REV pp29.	Research Infrastructure QualityNano
Nanomaterial name: Silica-NP<br>Particle Code: SilNP_005<br>Composition: SiO_2<br>Method of production: Stöber-Synthesis	**Manufacturer /Institute/Date**<br>**Eugene Mahon ; provided by UCD; 10/2011**<br>**Technology Expert:** Eugene Mahon
**Kind of suspension:**	**Suspension** ☒ **Powder** ☐<br>Suspended in: Pure water<br>pH: n.d.<br>stabilizer: none

**Property**		**unit**	**Method / Institute**	**Value**
Agglomeration/aggregation			HRTEM (KIT)	Agglomerated
Crystalline phase			D5000 Diffractometer (UU)<br>PANalytical X´Pert diffractometer (ICN)	Amorphous
Crystallite size			HRTEM (KIT)	n.a.
Octanol-water partition coefficient			Extraction/ ICP-MS (ICN)	n.a.
Photocatalytic activity			Rhodamin-B Bleaching/UV-B-100 10mg/ml in Water (ICN)	none
Porosity		-/- or %	n.a.	n.a.
Pour density		cm3/g	(BJH) Pore Size Distribution and Volume by ASAP2020 (UU)	n.a.
Size distribution: (suspension in water)	Modal value X_M (PDI)	nm	Zetasizer Nano ZS /UCD<br>DCS (UCD) (RWtAv / RNumAv)	64.4(PDI:0.091)/ NM: 43.9<br>45.5 / 41.9
	Total concentration	mg/ml		1 mg/ml
Size distribution: (Aerosol)	Modal value X_M (σ_{geo})	nm	SMPS TSI, DMA 3071 with CPC 3022A / KIT<br>dispersed by electrospray	52 nm (σ_{geo}=1.25)
	Total concentration	mg/cm³ or #/cm³		1.05x 10^5/cm^3
Solubility in _____		g/l		
Solubility in H_2O		g/l	Extraction/ ICP-MS (ICN)	165.0 g/L
Specific surface area		cm²/g	BET by ASAP2020 (UU)	n.a.
Surface chemistry				
Zeta potential (surface charge)		eV	Zetasizer Nano ZS /UU	-51.1 ± 13.9eV (pH 8.91)
Summary of trace analyses			ICP-MS / KIT	
Cu		µg/g		6.0

Electron microscopy

Method / technical data of microscope:

SOP: See KIT (LEM)

Magnification: 450.000

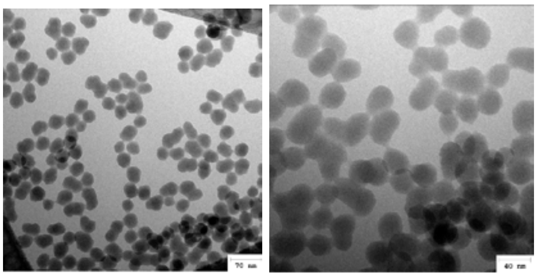

Electron Microscopy by KIT (LEM); Diameter : 30-50 nm

FEI Tecna i 120

SOP: see UCD

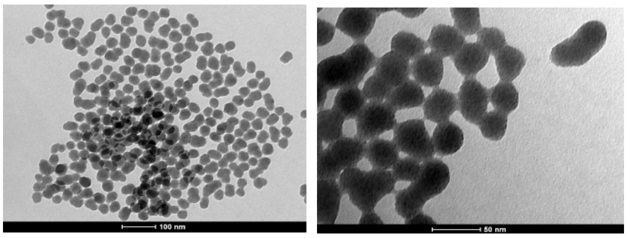

Electron Microscopy by UCD

Av. Diameter (ImageJ) = 40.6nm

Radical formation potential by DCF-Test:

(H_2O_2 as positive control) (KIT)

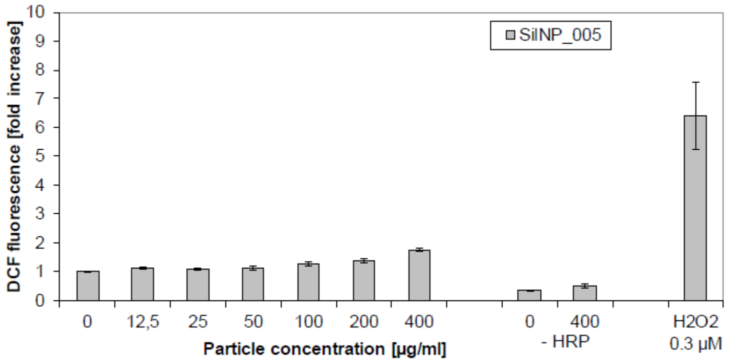

Result: SiINP_005 NPs induced a very low increase of DCFH oxidation at high concentrations indicating a negligible potential to produce reactive oxygen species.

SiINP_005_Corona Fingerprint_UCD

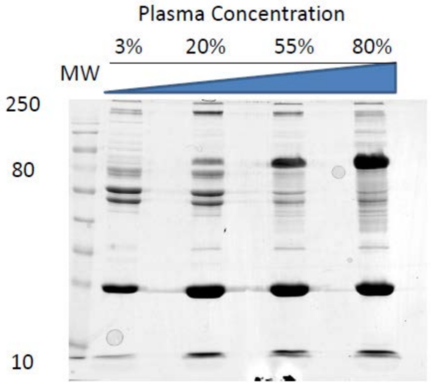

Comments and other relevant physical-chemical properties and material characterization information

**Place, Date Karlsruhe, 29.11.2016**	**Responsible H.-R. Paur, KIT**

**REPRESENTATIVE TEST PARTICLES**<br>Nanomaterial Identification<br>according to OECD<br>ENV/JM/MONO(2009)20/REV pp29.	Research Infrastructure QualityNano
Nanomaterial name: Silica-NP<br>Particle Code: SilNP_006<br>Composition: SiO_2<br>Method of production: Stöber-Synthesis	**Manufacturer /Institute/Date**<br>**Eugene Mahon ; provided by UCD; 10/2011**<br>**Technology Expert:** Eugene Mahon
**Kind of suspension:**	**Suspension** ☒ **Powder** ☐<br>Suspended in: Pure water<br>pH: n.d.<br>stabilizer: none

**Property**		**unit**	**Method / Institute**	**Value**
Agglomeration/aggregation			HRTEM (KIT)	Agglomerated
Crystalline phase			D5000 Diffractometer (UU)<br>PANalytical X´Pert diffractometer (ICN)	Amorphous
Crystallite size			HRTEM (KIT)	n.a.
Octanol-water partition coefficient			Extraction/ ICP-MS (ICN)	n.a.
Photocatalytic activity			Rhodamin-B Bleaching/UV-B-100 10mg/ml in Water (ICN)	medium
Porosity		-/- or %	n.a.	n.a.
Pour density		cm3/g	(BJH) Pore Size Distribution and Volume by ASAP2020 (UU)	n.a.
Size distribution: (suspension in water)	Modal value X_M (PDI)	nm	Zetasizer Nano ZS /UCD<br>DCS (UCD) (RWtAv / RNumAv)	87.7 (PDI:0.231) NM: 41.2<br>48.8/37.25
	Total concentration	mg/ml		1mg/ml
Size distribution: (Aerosol)	Modal value X_M (σ_{geo})	nm	SMPS TSI, DMA 3071 with CPC 3022A / KIT<br>dispersed by electrospray	58 (σ_{geo}=1.46)
	Total concentration	mg/cm³ or #/cm³		5.88x 10^4/cm^3
Solubility in _____		g/l		
Solubility in H_2O		g/l	Extraction/ ICP-MS (ICN)	55.6 g/L
Specific surface area		cm²/g	BET by ASAP2020 (UU)	n.a.
Surface chemistry				
Zeta potential (surface charge)		eV	Zetasizer Nano ZS /UU	-45.3±6.6eV (pH 7.72)
Summary of trace analyses			ICP-MS / KIT	
B		µg/g		40.0
Na		µg/g		32.0
K		µg/g		24.0
Ca		µg/g		82.0
Cu		µg/g		18.0
Zn		µg/g		6.2

<table>
<tr>
<td>Electron microscopy
Method / technical data of microscope:
SOP: See KIT (LEM)

Magnification: 450.000</td>
<td>
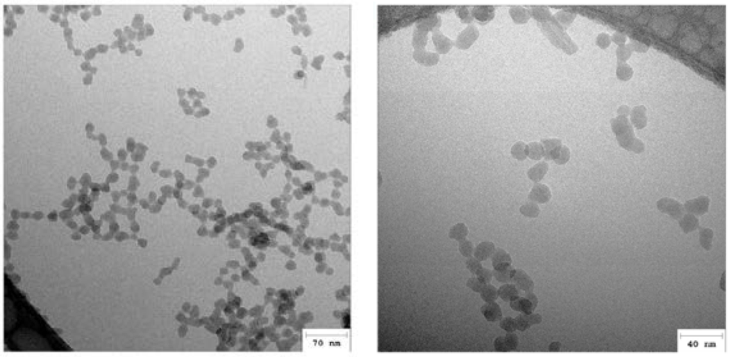

Electron Microscopy by KIT (LEM);
Diameter : Typical particle size is about 20 nm
</td>
</tr>
<tr>
<td>FEI Tecna i 120
SOP: see UCD</td>
<td>
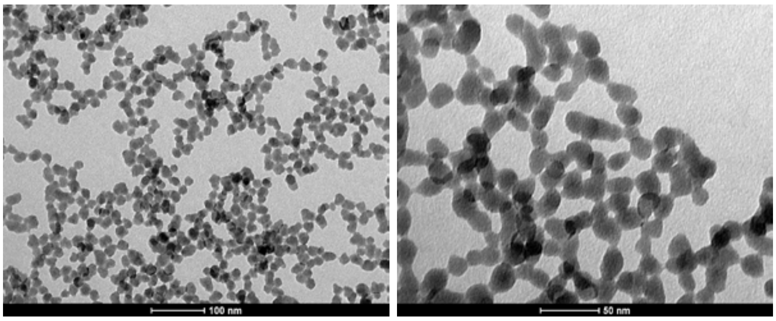

Electron Microscopy by UCD
Av. Diameter (ImageJ) = 21.92nm
</td>
</tr>
<tr>
<td>Radical formation potential by DCF-Test:
(H_2O_2 as positive control) (KIT)</td>
<td>
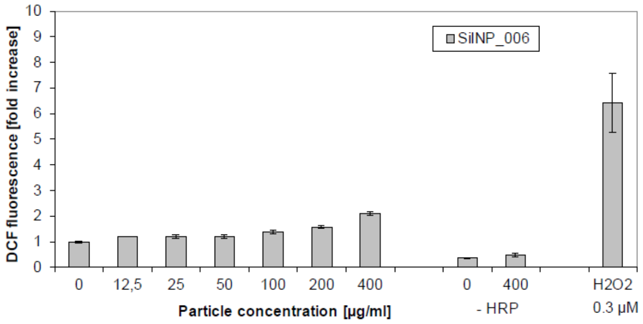

Result: SilNP_006 NPs induced a very low increase of DCFH oxidation at high concentrations indicating a negligible potential to produce reactive oxygen species.
</td>
</tr>
<tr>
<td>SilNP_006_Corona Fingerprint_UCD</td>
<td>
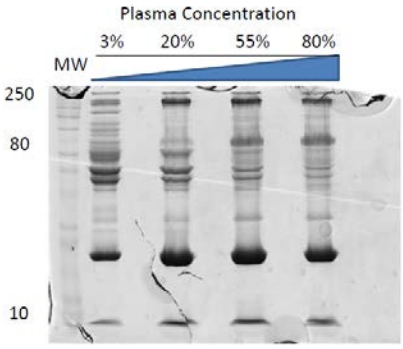

</td>
</tr>
<tr>
<td colspan="2">Comments and other relevant physical-chemical properties and material characterization information</td>
</tr>
<tr>
<td>Place, Date Karlsruhe, 29.11.2016</td>
<td>Responsible H.-R. Paur, KIT</td>
</tr>
</table>

**REPRESENTATIVE TEST PARTICLES**<br>Nanomaterial Identification according to OECD ENV/JM/MONO(2009)20/REV pp29.			Research Infrastructure QualityNano	
Nanomaterial name: Silica-NP<br>Particle Code: SilNP_007<br>Composition: SiO_2<br>Method of production: Stöber-Synthesis			**Manufacturer /Institute/Date**<br>**Eugene Mahon ; provided by UCD; 10/2011**<br>**Technology Expert:** Eugene Mahon	
**Kind of suspension:**			**Suspension** ☒ **Powder** ☐<br>Suspended in: Pure water<br>pH: n.d.<br>stabilizer: none	
**Property**		**unit**	**Method / Institute**	**Value**
Agglomeration/aggregation			HRTEM (KIT)	Agglomerated
Crystalline phase			D5000 Diffractometer (UU)<br>PANalytical X´Pert diffractometer (ICN)	Amorphous
Crystallite size			HRTEM (KIT)	n.a.
Octanol-water partition coefficient			Extraction/ ICP-MS (ICN)	n.a.
Photocatalytic activity			Rhodamin-B Bleaching/UV-B-100 10mg/ml in Water (ICN)	none
Porosity		-/- or %	n.a.	n.a.
Pour density		cm3/g	(BJH) Pore Size Distribution and Volume by ASAP2020 (UU)	n.a.
Size distribution: (suspension in water)	Modal value X_M (PDI)	nm	Zetasizer Nano ZS /UCD<br>DCS (UCD) (RWtAv / RNumAv)	67.2 (PDI:0.131) NM: 42.5<br>47.1/43.8
	Total concentration	mg/ml		1mg/ml
Size distribution: (Aerosol)	Modal value X_M (σ_{geo})	nm	SMPS TSI, DMA 3071 with CPC 3022A / KIT dispersed by electrospray	53.0
	Total concentration	mg/cm³ or #/cm³		$3.62 \times 10^4/cm^3$
Solubility in _____		g/l		
Solubility in H_2O		g/l	Extraction/ ICP-MS (ICN)	109.0 g/L
Specific surface area		cm²/g	BET by ASAP2020 (UU)	n.a.
Surface chemistry				
Zeta potential (surface charge)		eV	Zetasizer Nano ZS /UU	-66.2mV (pH 8.80)
Summary of trace analyses			ICP-MS (KIT)	
B		µg/g		4.7
Na		µg/g		21
Mg		µg/g		4.4
Al		µg/g		6.1
K		µg/g		13
Ca		µg/g		67
Fe		µg/g		2.7
Cu		µg/g		3.9

<table>
<tr><td>Zn</td><td>µg/g</td><td></td><td>3.4</td></tr>
<tr><td>Ba</td><td>µg/g</td><td></td><td>4.3</td></tr>
<tr><td>Electron microscopy
Method / technical data of microscope:
SOP: See KIT (LEM)

Magnification: 450.000

FEI Tecna i 120
SOP: see UCD</td><td colspan="3">
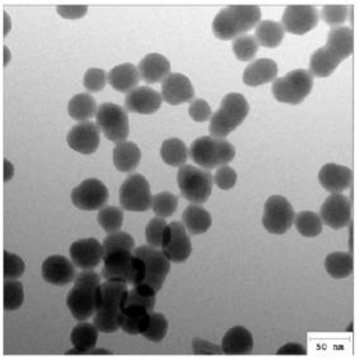

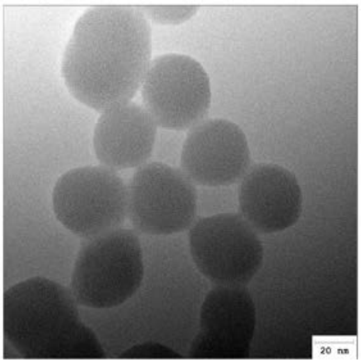

Electron Microscopy by KIT (LEM)
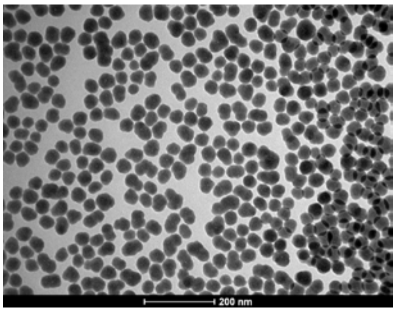

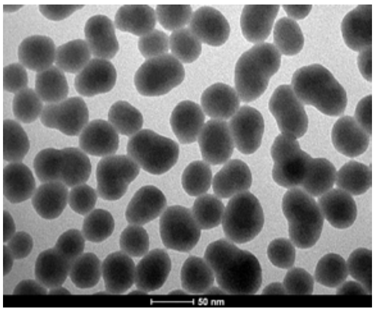

Electron Microscopy by UCD
Av. Diameter (ImageJ) = 44.3 nm (7.28)</td></tr>
<tr><td>Radical formation potential by DCF-Test:
(H_2O_2 as positive control) (KIT)</td><td colspan="3">
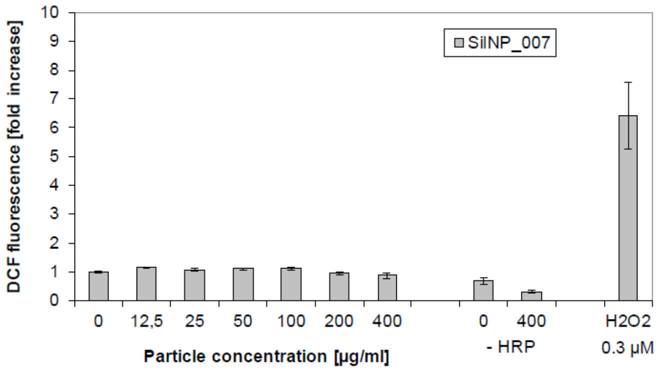

Result: SiINP_007 NPs induced no increase of DCFH oxidation at concentrations between 12.5 and 400 µg/ml.</td></tr>
<tr><td colspan="4">Comments and other relevant physical-chemical properties and material characterization information</td></tr>
<tr><td colspan="2">Place, Date Karlsruhe, 29.11.2016</td><td colspan="2">Responsible H.-R. Paur, KIT</td></tr>
</table>

REPRESENTATIVE TEST PARTICLES
Nanomaterial Identification
according to OECD
ENV/JM/MONO(2009)20/REV pp29.

Nanomaterial name	Silica-NP	**Manufacturer /Institute/Date**
Particle Code	SiINP_008	**Eugene Mahon ; provided by UCD; 10/2011**
Composition	SiO_2	**Technology Expert:** Eugene Mahon
Method of production	Stöber-Synthesis	

Kind of suspension:

Suspension ☒ **Powder** ☐

Suspended in	Pure water
pH	n.d.
stabilizer	none

**Property**		**unit**	**Method / Institute**	**Value**
Agglomeration/aggregation			HRTEM (KIT)	Agglomerated
Crystalline phase			D5000 Diffractometer (UU)<br>PANalytical X´Pert diffractometer (ICN)	Amorphous
Crystallite size			HRTEM (KIT)	n.a.
Octanol-water partition coefficient			Extraction/ ICP-MS (ICN)	n.a.
Photocatalytic activity			Rhodamin-B Bleaching/UV-B-100<br>10mg/ml in Water (ICN)	none
Porosity		-/- or %	n.a.	n.a
Pour density		cm3/g	(BJH) Pore Size Distribution and Volume by ASAP2020 (UU)	n.a
Size distribution:<br>(suspension in water)	Modal value X_M (PDI)	nm	Zetasizer Nano ZS /UCD<br>DCS (UCD) (RWtAv / RNumAv)	36.5 (PDI: 0.175) NM: 19.9<br>23.2/22.6
	Total concentration	mg/ml		1mg/ml
Size distribution:<br>(Aerosol)	Modal value X_M (σ_{geo})	nm	SMPS TSI, DMA 3071 with CPC 3022A / KIT<br>dispersed by electrospray	33 (σ_{geo}=1.17)
	Total concentration	mg/cm³ or #/cm³		8.3x 10^4/cm³
Solubility in _____		g/l		
Solubility in H_2O		g/l	Extraction/ ICP-MS (ICN)	62.37 g/L
Specific surface area		cm²/g	BET by ASAP2020 (UU)	n.a.
Surface chemistry				
Zeta potential (surface charge)		eV	Zetasizer Nano ZS /UU	-38.0mV<br>(pH 7.78)
Summary of trace analyses			ICP-MS (KIT)	
B		µg/g		4.9
Na		µg/g		35
Mg		µg/g		2.1
Al		µg/g		2.1
K		µg/g		30
Ca		µg/g		56
Fe		µg/g		2.1
Cu		µg/g		2.1

Zn	µg/g		1.7
Ba	µg/g		4

Electron microscopy

Method / technical data of microscope:

SOP: See KIT (LEM)

Magnification: 450.000

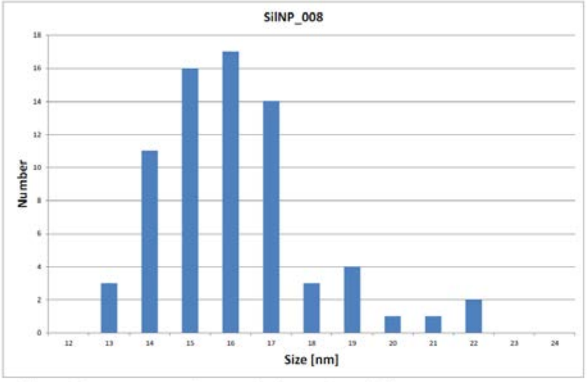

- The particles are very small, mean size is approximately 16 nm
- The size distribution is extremely narrow

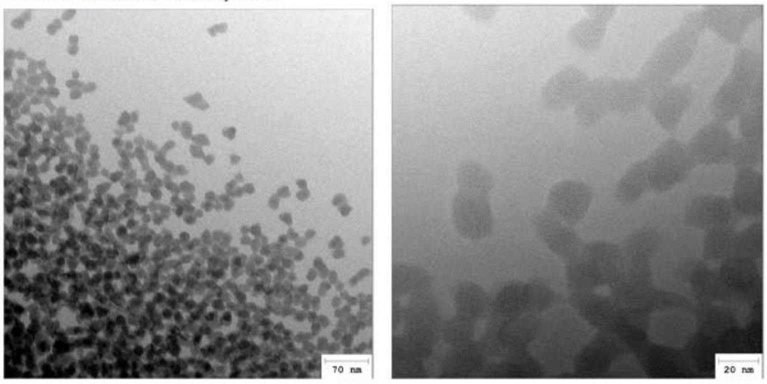

Electron Microscopy by KIT (LEM)

FEI Tecna i 120

SOP: see UCD

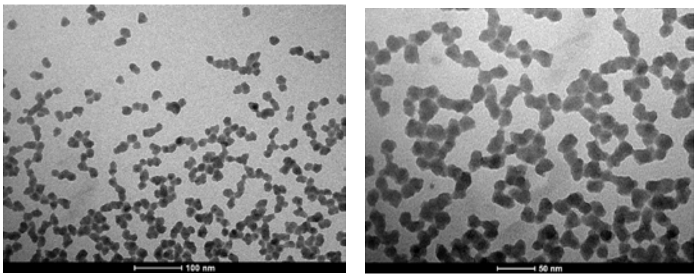

Electron Microscopy by UCD

Av. Diameter (ImageJ) = 26.6(5.16) nm

Radical formation potential by DCF-Test:

(H_2O_2 as positive control) (KIT)

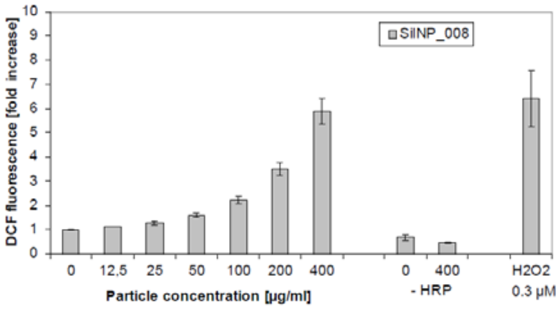

Result: SilNP_008 NPs induced a significant DCFH oxidation at high concentrations indicating a potential to produce reactive oxygen species.

Comments and other relevant physical-chemical properties and material characterization information

**Place, Date Karlsruhe, 29.11.2016**	**Responsible H.-R. Paur, KIT**

**REPRESENTATIVE TEST PARTICLES**<br>Nanomaterial Identification<br>according to OECD<br>ENV/JM/MONO(2009)20/REV pp29.	Research Infrastructure QualityNano
Nanomaterial name: Silica-NP<br>Particle Code: SilNP_009<br>Composition: SiO_2<br>Method of production: Stöber-Synthesis	**Manufacturer /Institute/Date**<br>**Eugene Mahon ; provided by UCD; 10/2011**<br>**Technology Expert:** Eugene Mahon
**Kind of suspension:**	**Suspension** ☒ **Powder** ☐<br>Suspended in: Pure water<br>pH: n.d.<br>stabilizer: none

**Property**		**unit**	**Method / Institute**	**Value**
Agglomeration/aggregation			HRTEM (KIT)	Agglomerated
Crystalline phase			D5000 Diffractometer /UU<br>PANalytical X´Pert diffractometer /ICN	Amorphous
Crystallite size			HRTEM (KIT)	n.a.
Octanol-water partition coefficient			Extraction/ ICP-MS (ICN)	n.a.
Photocatalytic activity			Rhodamin-B Bleaching/UV-B-100<br>10mg/ml in Water (ICN)	none
Porosity		-/- or %	n.a.	n.a.
Pour density		cm3/g	(BJH) Pore Size Distribution and Volume by ASAP2020 (UU)	n.a.
Size distribution:<br>(suspension in water)	Modal value X_M (PDI)	nm	Zetasizer Nano ZS /UCD<br><br>DCS (UCD) (RWtAv / RNumAv)	61.3 (PDI:0.093)<br>NM: 43.7<br>43/ 40.7
	Total concentration	mg/ml		1mg/ml
Size distribution:<br>(Aerosol)	Modal value X_M (σ_{geo})	nm	SMPS TSI, DMA 3071 with CPC 3022A / KIT<br>dispersed by electrospray	50
	Total concentration	mg/cm³ or #/cm³		$4.9x\ 10^4/cm^3$
Solubility in _____		g/l		
Solubility in H_2O		g/l	Extraction/ ICP-MS (ICN)	81.8 g/L
Specific surface area		cm²/g	BET by ASAP2020 (UU)	n.a.
Surface chemistry				
Zeta potential (surface charge)		eV	Zetasizer Nano ZS /UU	-51.7mV<br>(pH 8.72)
Summary of trace analyses			ICP-MS (KIT)	
B		µg/g		5.6
Na		µg/g		54
Mg		µg/g		1.9
Al		µg/g		3.2
K		µg/g		12
Ca		µg/g		14
Fe		µg/g		3.3
Cu		µg/g		12.5
Zn		µg/g		2.8
Ba		µg/g		2.6

**Electron microscopy**<br>Method / technical data of microscope:<br>SOP: See KIT (LEM)<br><br>Magnification: 450.000	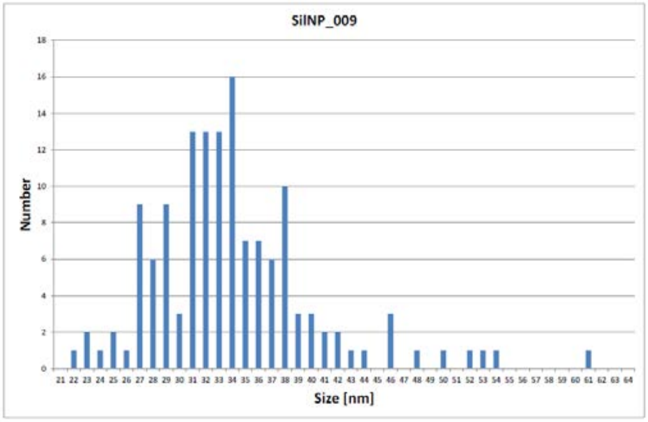<br>▪ The maximum of the size distribution is at about 34 nm<br>▪ A relatively broad distribution (from about 20 nm to 50 nm) is observable<br>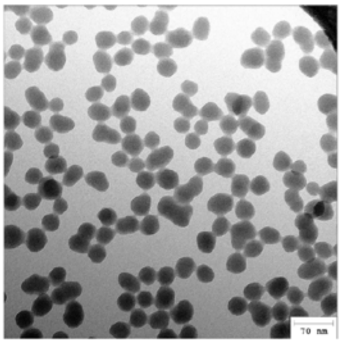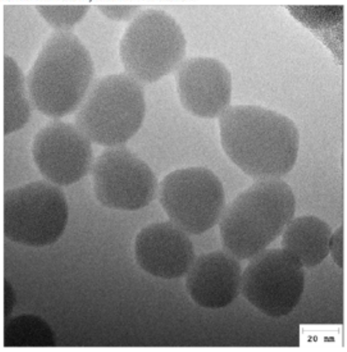<br>Electron Microscopy by KIT (LEM); Diameter : 30-50 nm
FEI Tecna i 120<br>SOP: see UCD	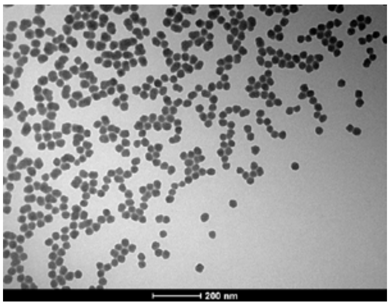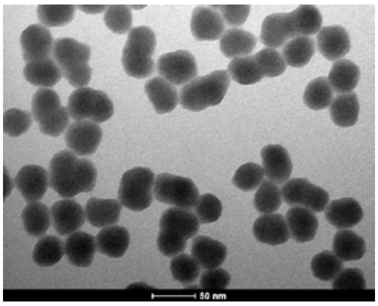<br>Electron Microscopy by UCD<br><br>Av. Diameter (ImageJ) = 41.4 (4.52)nm
Radical formation potential by DCF-Test:<br>(H_2O_2 as positive control) (KIT)	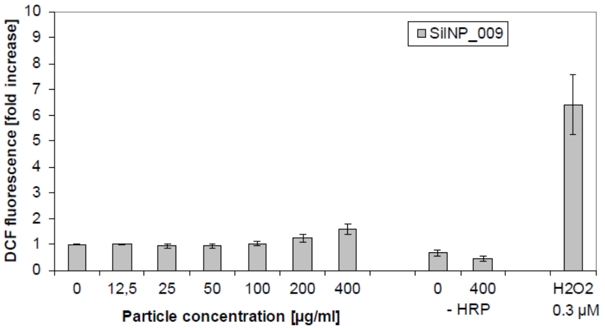<br>Result: SiINP_009 NPs induced a very low increase of DCFH oxidation at high concentrations indicating a negligible potential to produce reactive oxygen species.
**Comments and other relevant physical-chemical properties and material characterisation information**	
**Place, Date Karlsruhe, 29.11.2016**	**Responsible H.-R. Paur, KIT**

**REPRESENTATIVE TEST PARTICLES**<br>Nanomaterial Identification<br>according to OECD<br>ENV/JM/MONO(2009)20/REV pp29.	Research Infrastructure QualityNano
Nanomaterial name: Silica-NP<br>Particle Code: SilNP_010<br>Composition: SiO_2<br>Method of production: Stöber-Synthesis	**Manufacturer /Institute/Date**<br>**Eugene Mahon ; provided by UCD; 10/2011**<br>**Technology Expert:** Eugene Mahon
**Kind of suspension:**	**Suspension** ☒ **Powder** ☐<br>Suspended in: Pure water<br>pH: n.d.<br>stabilizer: none

**Property**		**unit**	**Method / Institute**	**Value**
Agglomeration/aggregation			HRTEM (KIT)	Agglomerated
Crystalline phase			D5000 Diffractometer (UU)<br>PANalytical X´Pert diffractometer (ICN)	Amorphous
Crystallite size			HRTEM (KIT)	n.a.
Octanol-water partition coefficient			Extraction/ ICP-MS (ICN)	n.a.
Photocatalytic activity			Rhodamin-B Bleaching/UV-B-100<br>10mg/ml in Water (ICN)	none
Porosity		-/- or %	n.a.	n.a.
Pour density		cm3/g	(BJH) Pore Size Distribution and Volume by ASAP2020 (UU)	n.a.
Size distribution:<br>(suspension in water)	Modal value X_M (PDI)	nm	Zetasizer Nano ZS /UCD<br><br>DCS (UCD) (RWtAv / RNumAv)	40.1 (PDI:0.174)<br>NM: 20.7<br>26.4/25.0
	Total concentration	mg/ml		1mg/ml
Size distribution:<br>(Aerosol)	Modal value X_M (σ_{geo})	nm	SMPS TSI, DMA 3071 with CPC 3022A / KIT<br>dispersed by electrospray	33 (σ_{geo}=1.00)
	Total concentration	mg/cm³ or #/cm³		2.2x 10^5/cm^3
Solubility in _____		g/l		
Solubility in H_2O		g/l	Extraction/ ICP-MS (ICN)	117.4 g/L
Specific surface area		cm²/g	BET by ASAP2020 (UU)	n.a.
Surface chemistry				
Zeta potential (surface charge)		eV	Zetasizer Nano ZS /UU	-39.9mV<br>(pH 7.69)
Summary of trace analyses			ICP-MS (KIT)	
B		µg/g		5.6
Na		µg/g		47
Mg		µg/g		2
Al		µg/g		2.
K		µg/g		12
Ca		µg/g		15
Fe		µg/g		1.7
Cu		µg/g		11.1

<table>
<tr><td>Zn</td><td>µg/g</td><td></td><td>3.3</td></tr>
<tr><td>Ba</td><td>µg/g</td><td></td><td>2.9</td></tr>
<tr><td>

Electron microscopy

Method / technical data of microscope:

SOP: See KIT (LEM)

Magnification: 450.000

FEI Tecna i 120

SOP: see UCD

</td><td colspan="3">

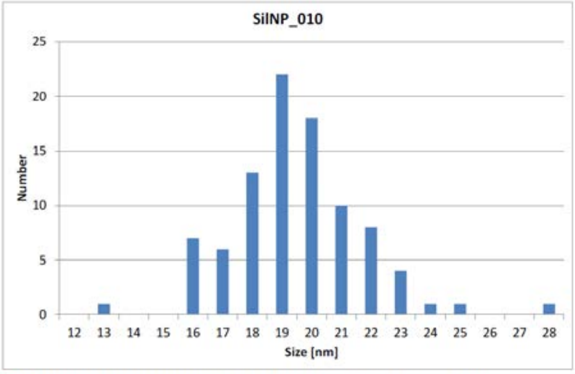

Mean particle size amounts to 18 - 20 nm, the size distribution is very narrow

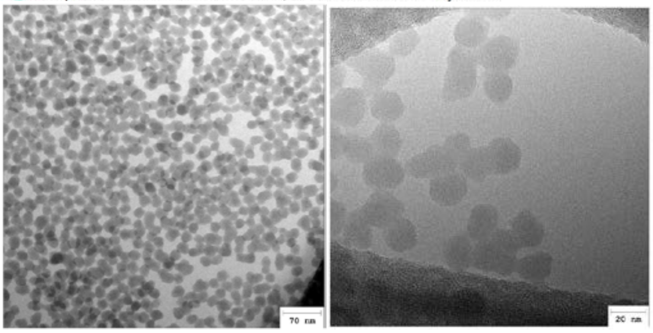

Electron Microscopy by KIT (LEM)

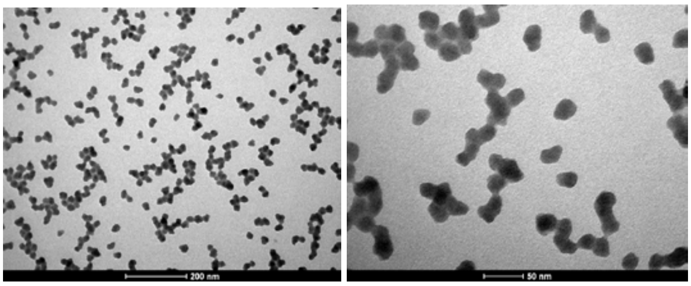

Electron Microscopy by UCD

Av. Diameter (ImageJ) = 30.93 (5.1) nm

</td></tr>
<tr><td>

Radical formation potential by DCF-Test:

(H_2O_2 as positive control) (KIT)

</td><td colspan="3">

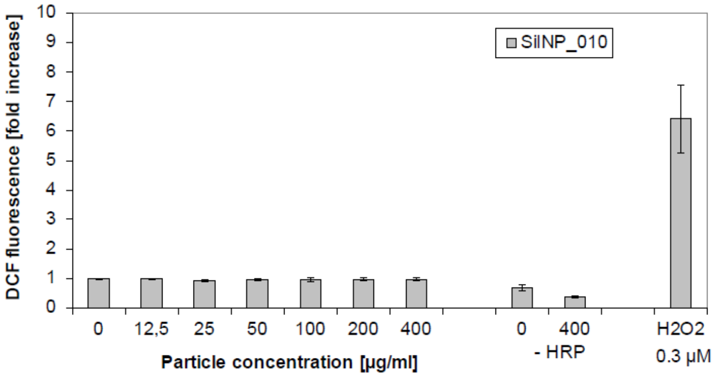

Result: SiINP_010 NPs induced no increase of DCFH oxidation at concentrations between 12.5 and 400 µg/ml.

</td></tr>
<tr><td colspan="4">

Comments and other relevant physical-chemical properties and material characterization information

</td></tr>
<tr><td colspan="2">

Place, Date Karlsruhe, 29.11.2016

</td><td colspan="2">

Responsible H.-R. Paur, KIT

</td></tr>
</table>

Supplemental S3: Physicochemical characterization data sheets of silica NMs from flame synthesis

**REPRESENTATIVE TEST PARTICLES**<br>Nanomaterial Identification<br>according to OECD<br>ENV/JM/MONO(2009)20/REV pp29.	Research Infrastructure QualityNano
Nanomaterial name: Aerosil®200<br>Particle Code: SilNP_001<br>Composition: SiO_2<br>Method of production: Flame synthesis	**Manufacturer /Institute:**<br>**DEGUSSA, provided by KIT – ITC**<br>**Name:** Sonja Mülhopt
**Kind of suspension:**	**Suspension** ☐ **Powder** ☒<br>Suspended in ________<br>pH ________<br>stabilizer ________

**Property**		**unit**	**Method (Institute)**	**Value**
Agglomeration/aggregation			HRTEM (KIT)	aggregated
Crystalline phase		n.a.	D5000 (UU)<br>PANalytical X´Pert diffractometer	amorphous
Crystallite size			HRTEM (KIT)	n.a.
Octanol-water partition coefficient			Extraction/ ICP-MS (ICN)	P_{OW}=0
Photocatalytic activity			Rhodamin-B Bleaching/UV-B-100<br>10mg/ml in Water (ICN)	80% / 2 min
Porosity		cm3/g	(BJH) Pore Size Distribution and Volume by ASAP2020 (UU)	0.43
Pour density		g/l		
Redox potential				
Size distribution (suspension in water)	Modal value X_M (PDI)	nm	Zetasizer Nano ZS (UCD)<br>(after 1h sonification)	142.7<br>(PDI=0.145)
	Total concentration	mg/ml		0.1 mg/ml
Size distribution (aerosol)	Modal value X_M (σ_{geo})	nm	SMPS TSI, DMA 3071 with CPC 3022A (KIT)<br>dispersed by rotating brush generator	127 (σ_{geo}=1.4)
	Total concentration	mg/cm³ or #/cm³		2.0 x 10^4 / cm³
Solubility in _____		g/l		
Solubility in H_2O		g/l	Extraction/ ICP-MS (ICN)	23.7±1.7
Specific surface area		m²/g	BET by ASAP2020 (UU)	196
Surface chemistry				
Zeta potential (surface charge)			Zetasizer Nano ZS (UU)	-22.8±0.8eV<br>(pH 5.00)

Summary of trace analyses		ICP-MS (KIT)	
Ni	µg/g		0.1
Cu	µg/g		0.2

**Electron microscopy**<br>Method / technical data of microscope:<br><br>See KIT-LEM<br><br>Magnification: ________<br><br>FEI Tecna i 120 (SOP: see UCD)	Electron Microscopy by KIT (LEM)<br>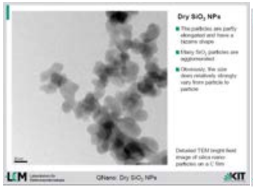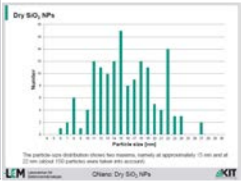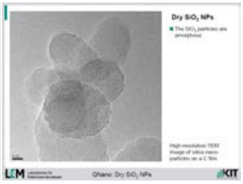<br>Average Size of Primary Particles: 15 ± 10 nm<br><br>Electron Microscopy by KIT (LEM)<br>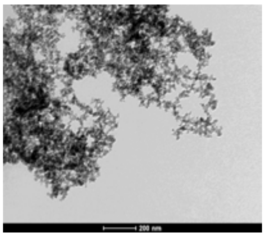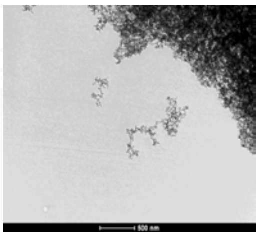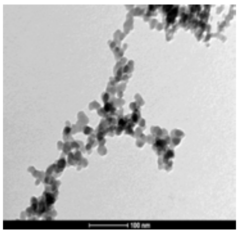<br>Primary particle size looks to around 20 nm from 10 distance measurements using ImageJ.
Radical formation potential by DCF-Test:<br>(H_2O_2 as positive control) (KIT)	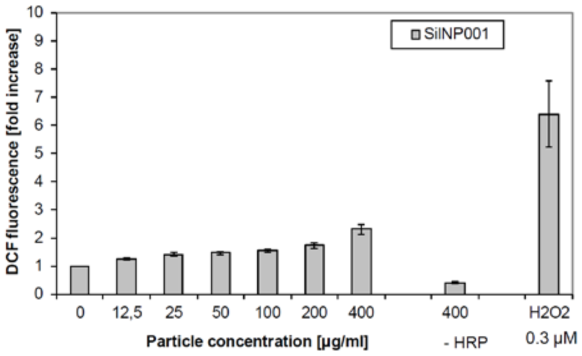<br>Result: SiINP_001 NPs induced a very low increase of DCFH oxidation at high concentrations indicating a negligible potential to produce reactive oxygen species.

Comments and other relevant physical-chemical properties and material characterization information

**Place, Date Karlsruhe, 29.11.2016**	**Responsible H.-R. Paur, KIT**

REPRESENTATIVE TEST PARTICLES
Nanomaterial Identification
according to OECD
ENV/JM/MONO(2009)20/REV pp29.

Research Infrastructure QualityNano

Nanomaterial name	Silica-NP
Particle Code	SilNP_012
Composition	SiO_2
Method of production	Flame-Synthesis

Manufacturer /Institute/Date

Christopher Anderlohr ; provided by KIT; 10/2011

Technology Expert: Dipl.-Ing. Christopher Anderlohr

Kind of suspension:

Suspension ☐ **Powder** ☒

Suspended in

pH ________

stabilizer none______

**Property**		**unit**	**Method / Institute**	**Value**
Agglomeration/aggregation			HRTEM (KIT)	Agglomerated
Crystalline phase			D5000 Diffractometer (UU)<br>PANalytical X´Pert diffractometer (ICN)	Amorphous
Crystallite size			HRTEM (KIT)	n.a.
Octanol-water partition coefficient			Extraction/ ICP-MS (ICN)	P_{OW}=0
Photocatalytic activity			Rhodamin-B Bleaching/UV-B-100<br>10mg/ml in Water (ICN)	none
Porosity		-/- or %	n.a.	non-porous
Pour density		cm3/g	(BJH) Pore Size Distribution and Volume by ASAP2020 (UU)	0.14
Size distribution:<br>(suspension in water)	Modal value X_M (PDI)	nm	Zetasizer Nano ZS /UCD<br>DCS (UCD) (RWtAv / RNumAv)	214.2 (PDI:0.215)/ NM: 125.2<br>n.a.
	Total concentration	mg/ml		1mg/ml
Size distribution:<br>(Aerosol)	Modal value X_M (σ_{geo})	nm	SMPS TSI. DMA 3071 with CPC 3022A / KIT<br>dispersed by electrospray	160 (σ_{geo}=1.59)
	Total concentration	mg/cm³ or #/cm³		n.a.
Solubility in _____		g/l		
Solubility in H_2O after 30 days		mg/ml	Extraction/ ICP-MS (ICN)	6
Specific surface area		cm²/g	BET by ASAP2020 (UU)	72.3
Surface chemistry				
Zeta potential (surface charge)		eV	Zetasizer Nano ZS /UU	-16.2 ± 5.8mV<br>(pH 4.58)

Summary of trace analyses		ICP-MS / KIT	
Na	µg/g		6.6
Mg	µg/g		1.9
Al	µg/g		22.6
K	µg/g		6.6
Ca	µg/g		20.3
Ti	µg/g		0.7
Cr	µg/g		< 1
Mn	µg/g		0.20
Fe	µg/g		12.4
Ni	µg/g		<0.5
Cu	µg/g		0.26
Zn	µg/g		1.6
Pb	µg/g		0.13

Electron microscopy
Method / technical data of microscope:
SOP: See KIT (LEM)

Magnification: 450.000

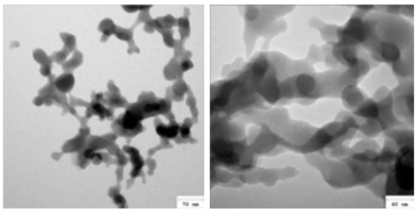

Electron Microscopy by KIT (LEM); Diameter : 10-60 nm

FEI Tecna i 120
SOP: see UCD

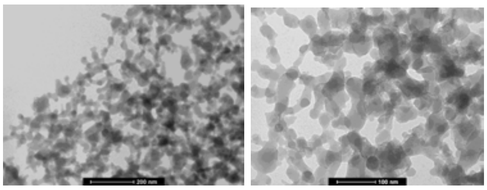

Electron Microscopy by UCD
Av. Diameter (ImageJ) ~49nm (Std. Dev 12.77)

UNIVLEEDS
TEM/ SEM-EDX

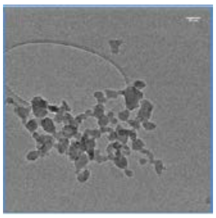

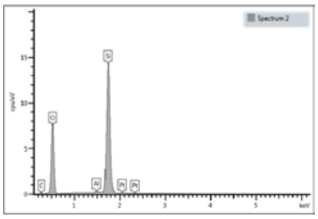

SiINP 0012 Av. Diameter =48.93 nm

Radical formation potential by DCF-Test:
(H_2O_2 as positive control) (KIT)

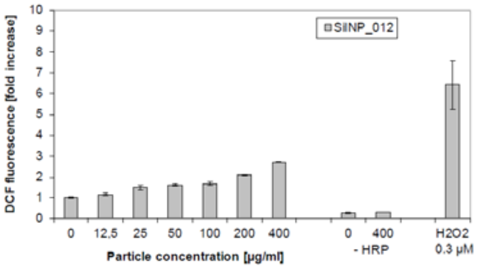

Result: SiINP_012 NPs induced a weak increase of DCFH oxidation at concentrations from 12.5 and 400 µg/ml.

Comments and other relevant physical-chemical properties and material characterization information

**Place, Date Karlsruhe, 29.11.2016**	**Responsible H.-R. Paur. KIT**

**REPRESENTATIVE TEST PARTICLES**<br>Nanomaterial Identification<br>according to OECD<br>ENV/JM/MONO(2009)20/REV pp29.	Research Infrastructure QualityNano
Nanomaterial name Silica-NP<br>Particle Code SiINP_013<br><br>Composition SiO_2<br>Method of production Flame-Synthesis	**Manufacturer /Institute/Date**<br><br>**Christopher Anderlohr ; provided by KIT; 10/2011**<br><br>**Technology Expert:** Dipl.-Ing. Christopher Anderlohr
**Kind of suspension:**	**Suspension** ☐ **Powder** ☒<br>Suspended in<br>pH ____________<br>stabilizer none_________

**Property**		**unit**	**Method / Institute**	**Value**
Agglomeration/aggregation			HRTEM (KIT)	Agglomerated
Crystalline phase			D5000 Diffractometer (UU)<br>PANalytical X´Pert diffractometer (ICN)	Amorphous
Crystallite size			HRTEM (KIT)	n.a.
Octanol-water partition coefficient			Extraction/ ICP-MS (ICN)	P_{OW}=0
Photocatalytic activity			Rhodamin-B Bleaching/UV-B-100<br>10mg/ml in Water (ICN)	none
Porosity		-/- or %	n.a.	non-porous
Pour density		cm3/g	(BJH) Pore Size Distribution and Volume by ASAP2020 (UU)	0.13
Size distribution:<br>(suspension in water)	Modal value X_M (PDI)	nm	Zetasizer Nano ZS /UCD<br><br>DCS (UCD) (RWtAv / RNumAv)	187.2 (PDI:0.18)/ NM: 122.8<br><br>n.a.
	Total concentration	mg/ml		1mg/ml
Size distribution:<br>(Aerosol)	Modal value X_M (σ_{geo})	nm	SMPS TSI, DMA 3071 with CPC 3022A / KIT<br>dispersed by electrospray	166 (σ_{geo}=1.55)
	Total concentration	mg/cm³ or #/cm³		n.a.
Solubility in _____		g/l		
Solubility in H_2O after 30 days		mg/ml	Extraction/ ICP-MS (ICN)	8.75
Specific surface area		cm²/g	BET by ASAP2020 (UU)	77.5
Surface chemistry				
Zeta potential (surface charge)		eV	Zetasizer Nano ZS /UU	-16.2 ± 7.4mV<br>(pH 4.53)

Summary of trace analyses		ICP-MS / KIT	
Na	µg/g		8.5
Mg	µg/g		1.3
Al	µg/g		3.2
K	µg/g		6.0
Ca	µg/g		6.2
Mn	µg/g		0.15
Fe	µg/g		8.3
Cu	µg/g		0.26
Zn	µg/g		1.1

**Electron microscopy**<br>Method / technical data of microscope:<br>SOP: See KIT (LEM)<br><br>Magnification: 450.000	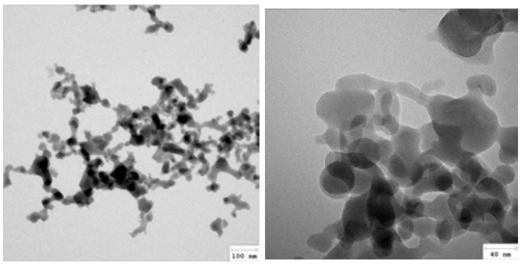<br>Electron Microscopy by KIT (LEM); Diameter : 10-60 nm
FEI Tecna i 120<br>SOP: see UCD	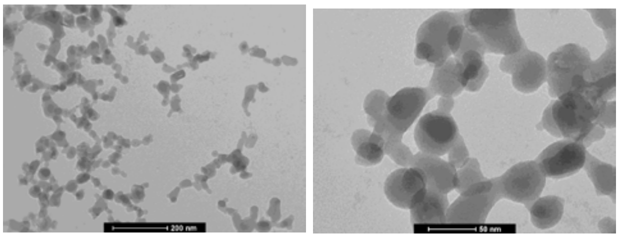<br>Electron Microscopy by UCD<br>Av. Diameter (ImageJ) ≈ 38nm (Std. Dev 9.18)
UNIVLEEDS<br>TEM/ SEM-EDX	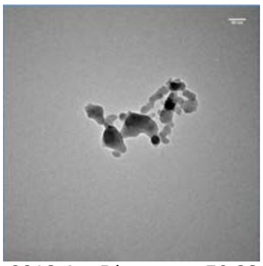 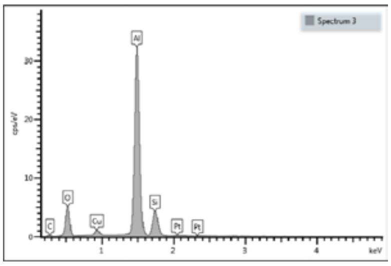<br>SiINP 0013 Av. Diameter =56.83 nm
Radical formation potential by DCF-Test:<br>(H_2O_2 as positive control) (KIT)	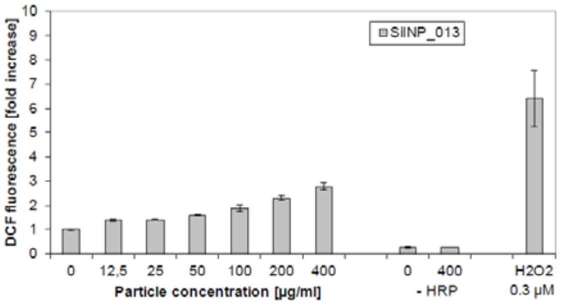<br>Result: SiINP_013 NPs induced a weak increase of DCFH oxidation at concentrations from 12.5 and 400 µg/ml.

Comments and other relevant physical-chemical properties and material characterization information

**Place, Date Karlsruhe, 29.11.2016**	**Responsible H.-R. Paur, KIT**

REPRESENTATIVE TEST PARTICLES
Nanomaterial Identification
according to OECD
ENV/JM/MONO(2009)20/REV pp29.

Research Infrastructure QualityNano

Nanomaterial name	Silica-NP	**Manufacturer /Institute/Date**
Particle Code	SiINP_014	**Christopher Anderlohr ; provided by KIT; 10/2011**
Composition	SiO_2	**Technology Expert:** Dipl.-Ing. Christopher Anderlohr
Method of production	Flame-Synthesis	

Kind of suspension:

Suspension ☐ **Powder** ☒

Suspended in
pH ____________
stabilizer none________

**Property**		**unit**	**Method / Institute**	**Value**
Agglomeration/aggregation			HRTEM (KIT)	Agglomerated
Crystalline phase			D5000 Diffractometer (UU)<br>PANalytical X´Pert diffractometer (ICN)	Amorphous
Crystallite size			HRTEM (KIT)	n.a.
Octanol-water partition coefficient			Extraction/ ICP-MS (ICN)	P_{OW}=0
Photocatalytic activity			Rhodamin-B Bleaching/UV-B-100<br>10mg/ml in Water (ICN)	none
Porosity		-/- or %	n.a.	non-porous
Pour density		cm3/g	(BJH) Pore Size Distribution and Volume by ASAP2020 (UU)	0.13
Size distribution:<br>(suspension in water)	Modal value X_M (PDI)	nm	Zetasizer Nano ZS /UCD	**H_2O**: 217.9 (PDI:0.275)/ NM: 113.1<br>**HNO_3**: 1667 (PDI:0.641)/ NM: 825.6<br>**NaOH**: 344.4 (PDI:0.434)/ NM: 114.1
			DCS (UCD) (RWtAv / RNumAv)	n.a.
	Total concentration	mg/ml		1mg/ml
Size distribution:<br>(Aerosol)	Modal value X_M (σ_{geo})	nm	SMPS TSI, DMA 3071 with CPC 3022A / KIT<br>dispersed by electrospray	32.5 (σ_{geo}=1.27)
	Total concentration	mg/cm³ or #/cm³		1.6x 10^5
Solubility in _____		g/l		
Solubility in H_2O after 30 days		mg/ml	Extraction/ ICP-MS (ICN)	6.5
Specific surface area		cm²/g	BET by ASAP2020 (UU)	76.1
Surface chemistry				
Zeta potential (surface charge)		eV	Zetasizer Nano ZS /UU	-15.5 ± 6.9mV<br>(pH 4.39)

Summary of trace analyses		ICP-MS / KIT	
Na	µg/g		4.5
Mg	µg/g		0.5
Al	µg/g		1.0
Ca	µg/g		6.2
Mn	µg/g		<0.05
Fe	µg/g		8.5
Ni	µg/g		<0.5
Cu	µg/g		0.15
Zn	µg/g		0.6
Pb	µg/g		<0.05

Electron microscopy
Method / technical data of microscope:
SOP: See KIT (LEM)

Magnification: 450.000

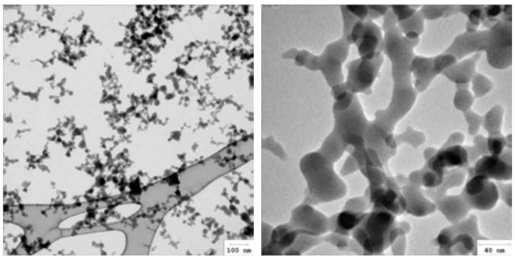

Electron Microscopy by KIT (LEM); Diameter : 10-80 nm

FEI Tecna i 120
SOP: see UCD

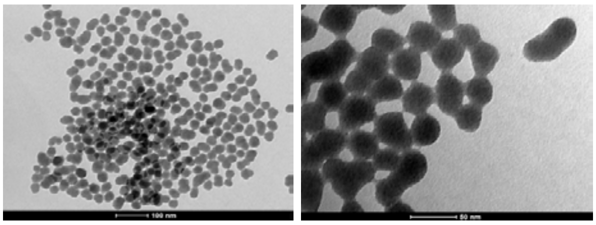

Electron Microscopy by UCD
Av. Diameter (ImageJ) = 40.6nm

UNIVLEEDS
TEM/ SEM-EDX

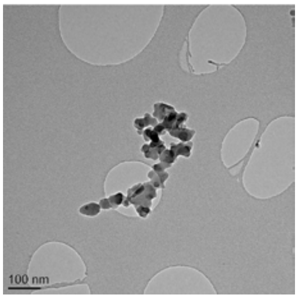

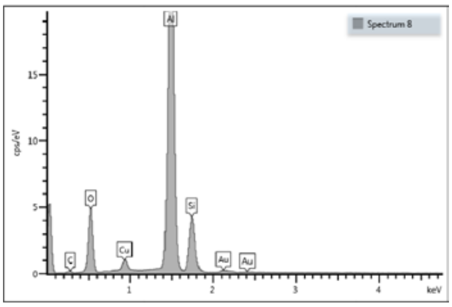

Radical formation potential by DCF-Test:
(H_2O_2 as positive control) (KIT)

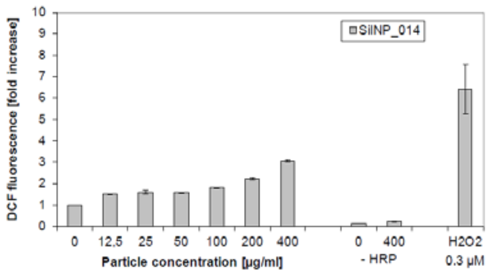

Result: SiINP_014 NPs induced a weak increase of DCFH oxidation at concentrations from 12.5 and 400 µg/ml.

Comments and other relevant physical-chemical properties and material characterization information

**Place, Date Karlsruhe, 29.11.2016**	**Responsible H.-R. Paur, KIT**

**REPRESENTATIVE TEST PARTICLES**<br>Nanomaterial Identification<br>according to OECD<br>ENV/JM/MONO(2009)20/REV pp29.	Research Infrastructure QualityNano
Nanomaterial name: Silica-NP<br>Particle Code: SiINP_015<br><br>Composition: SiO_2<br>Method of production: Flame-Synthesis	**Manufacturer /Institute/Date**<br>**Christopher Anderlohr ; provided by KIT; 10/2011**<br>**Technology Expert:** Dipl.-Ing. Christopher Anderlohr
**Kind of suspension:**	**Suspension** ☐ **Powder** ☒<br>Suspended in<br>pH __________<br>stabilizer none________

**Property**		**unit**	**Method / Institute**	**Value**
Agglomeration/aggregation			HRTEM (KIT)	Agglomerated
Crystalline phase			D5000 Diffractometer (UU)<br>PANalytical X´Pert diffractometer (ICN)	Amorphous
Crystallite size			HRTEM (KIT)	n.a.
Octanol-water partition coefficient			Extraction/ ICP-MS (ICN)	P_{OW}=0
Photocatalytic activity			Rhodamin-B Bleaching/UV-B-100<br>10mg/ml in Water (ICN)	none
Porosity		-/- or %	n.a.	non-porous
Pour density		cm3/g	(BJH) Pore Size Distribution and Volume by ASAP2020 (UU)	0.06
Size distribution:<br>(suspension in water)	Modal value X_M (PDI)	nm	Zetasizer Nano ZS /UCD<br><br>DCS (UCD) (RWtAv / RNumAv)	**H_2O**: 434.1 (PDI: 0.293)/ NM: 98.2<br>**HNO_3**: 1283 (PDI: 0.425)/ NM: 955.75<br>**NaOH**: 384.9 (PDI: 0.261)/ NM: 318.9<br>n.a.
	Total concentration	mg/ml		1mg/ml
Size distribution:<br>(Aerosol)	Modal value X_M (σ_{geo})	nm	SMPS TSI, DMA 3071 with CPC 3022A / KIT<br>dispersed by electrospray	36.3 (σ_{geo}=1.21)
	Total concentration	mg/cm³ or #/cm³		1.2x 10^4
Solubility in _____		g/l		
Solubility in H_2O after 30 days		mg/ml	Extraction/ ICP-MS (ICN)	7
Specific surface area		cm²/g	BET by ASAP2020 (UU)	29.4
Surface chemistry				
Zeta potential (surface charge)		eV	Zetasizer Nano ZS /UU	-19.0 ± 7.7mV<br>(pH 4.34)

Summary of trace analyses		ICP-MS / KIT	
Li	µg/g		<0.1
Na	µg/g		4.3
Mg	µg/g		3.4
Al	µg/g		12
Ca	µg/g		7.8
Mn	µg/g		0.25
Fe	µg/g		6.7
Ni	µg/g		0.7
Cu	µg/g		0.49
Zn	µg/g		1.7
Pb	µg/g		<0.1

Electron microscopy
Method / technical data of microscope:
SOP: See KIT (LEM)

Magnification: 450.000

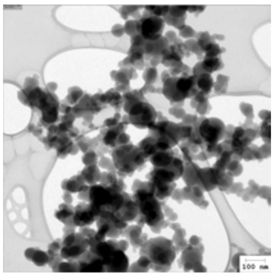

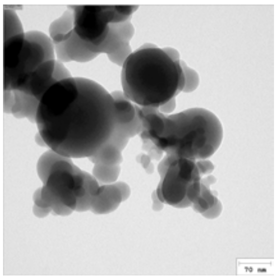

Electron Microscopy by KIT (LEM); Diameter : 15-150 nm

UNIVLEEDS
TEM/ SEM-EDX

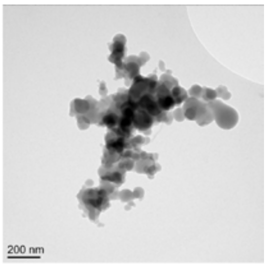

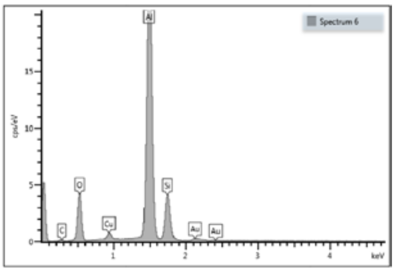

Radical formation potential by DCF-Test:

(H_2O_2 as positive control) (KIT)

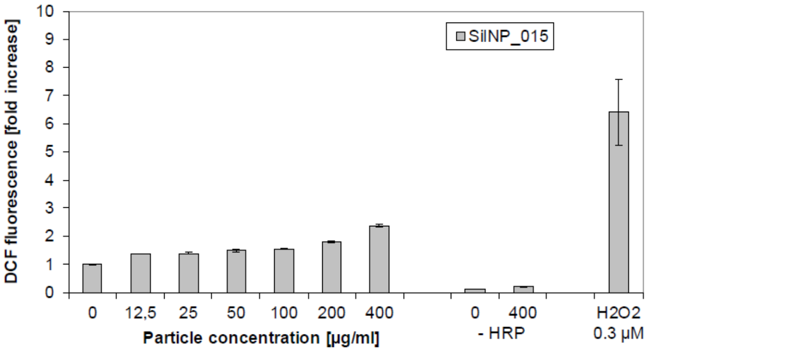

Result: SiINP_015 NPs induced a weak increase of DCFH oxidation at concentrations from 12.5 and 400 µg/ml.

Comments and other relevant physical-chemical properties and material characterization information

**Place, Date Karlsruhe, 29.11.2016**	**Responsible H.-R. Paur, KIT**

**REPRESENTATIVE TEST PARTICLES**<br>Nanomaterial Identification<br>according to OECD<br>ENV/JM/MONO(2009)20/REV pp29.	Research Infrastructure QualityNano
Nanomaterial name: Silica-NP<br>Particle Code: SiINP_016<br><br>Composition: SiO_2<br>Method of production: Flame-Synthesis	**Manufacturer /Institute/Date**<br>**Christopher Anderlohr ; provided by KIT; 10/2011**<br>**Technology Expert:** Christopher Anderlohr
**Kind of suspension:**	**Suspension** ☐ **Powder** ☒<br>Suspended in<br>pH ____________<br>stabilizer none_________

**Property**		**unit**	**Method / Institute**	**Value**
Agglomeration/aggregation			HRTEM (KIT)	Agglomerated
Crystalline phase			D5000 Diffractometer (UU)<br>PANalytical X´Pert diffractometer (ICN)	Amorphous
Crystallite size			HRTEM (KIT)	n.a.
Octanol-water partition coefficient			Extraction/ ICP-MS (ICN)	P_{OW}=0
Photocatalytic activity			Rhodamin-B Bleaching/UV-B-100<br>10mg/ml in Water (ICN)	none
Porosity		-/- or %	n.a.	non-porous
Pour density		cm3/g	(BJH) Pore Size Distribution and Volume by ASAP2020 (UU)	0.07
Size distribution:<br>(suspension in water)	Modal value X_M (PDI)	nm	Zetasizer Nano ZS /UCD	**H_2O**: 253.3 (PDI: 0.276)/ NM: 160.15<br>**HNO_3**: 1486 (PDI: 0.498)/ NM: 893.95<br>**NaOH**: 151 (PDI: 0.348)/ NM: 31.68
			DCS (UCD) (RWtAv / RNumAv)	n.a.
	Total concentration	mg/ml		1mg/ml
Size distribution:<br>(Aerosol)	Modal value X_M (σ_{geo})	nm	SMPS TSI, DMA 3071 with CPC 3022A / KIT<br>dispersed by electrospray	37.7 (σ_{geo}=1.26)
	Total concentration	mg/cm³ or #/cm³		n.a.
Solubility in _____		g/l		
Solubility in H_2O after 30 days		mg/ml	Extraction/ ICP-MS (ICN)	9.5
Specific surface area		cm²/g	BET by ASAP2020 (UU)	31.1
Surface chemistry				
Zeta potential (surface charge)		eV	Zetasizer Nano ZS /UU	-18.1 ± 5.6mV<br>(pH 4.29)

Summary of trace analyses		ICP-MS / KIT	
Li	µg/g		<0.1
Na	µg/g		6.0
Mg	µg/g		2.0
Al	µg/g		2.8
K	µg/g		<2
Ca	µg/g		7.4
Mn	µg/g		<0.05
Fe	µg/g		4.8
Ni	µg/g		0.6
Cu	µg/g		0.49
Zn	µg/g		1.3
**Electron microscopy**<br>Method / technical data of microscope:<br>SOP: See KIT (LEM)<br><br>Magnification: 450.000	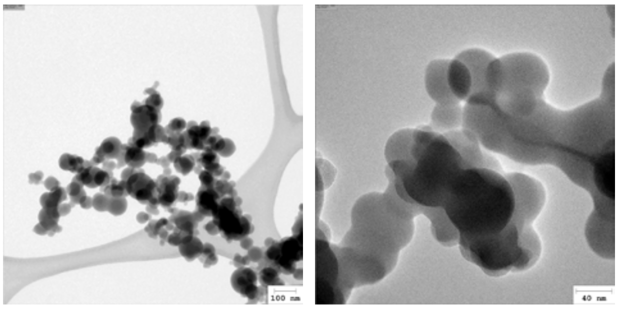<br><br>Electron Microscopy by KIT (LEM); Diameter : 20-120 nm		
UNIVLEEDS<br>TEM/ SEM-EDX	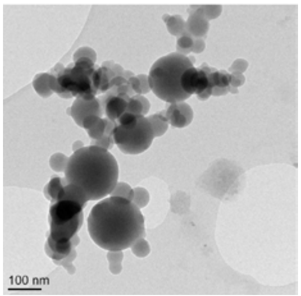<br><br>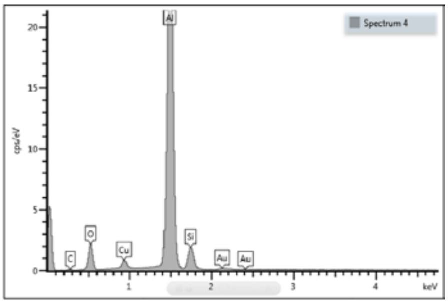 		
Radical formation potential by DCF-Test:<br>(H_2O_2 as positive control) (KIT)	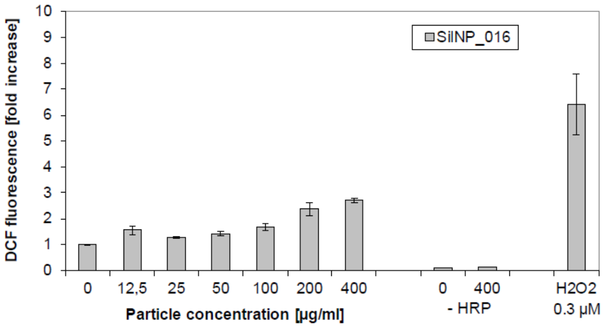<br><br>Result: SiINP_016 NPs induced a weak increase of DCFH oxidation at concentrations from 12.5 and 400 µg/ml.		
**Comments and other relevant physical-chemical properties and material characterization information**			
**Place, Date Karlsruhe, 29.11.2016**	**Responsible H.-R. Paur, KIT**		

**REPRESENTATIVE TEST PARTICLES**<br>Nanomaterial Identification<br>according to OECD<br>ENV/JM/MONO(2009)20/REV pp29.	Research Infrastructure QualityNano
Nanomaterial name: Silica-NP<br>Particle Code: SilNP_017<br>Composition: SiO_2<br>Method of production: Flame-Synthesis	**Manufacturer /Institute/Date**<br>**Christopher Anderlohr ; provided by KIT; 10/2011**<br>**Technology Expert:** Christopher Anderlohr
**Kind of suspension:**	**Suspension** ☐ **Powder** ☒<br>Suspended in<br>pH ____________<br>stabilizer none_________

**Property**		**unit**	**Method / Institute**	**Value**
Agglomeration/aggregation			HRTEM (KIT)	Agglomerated
Crystalline phase			D5000 Diffractometer (UU)<br>PANalytical X´Pert diffractometer (ICN)	Amorphous
Crystallite size			HRTEM (KIT)	n.a.
Octanol-water partition coefficient			Extraction/ ICP-MS (ICN)	P_{OW}=0
Photocatalytic activity			Rhodamin-B Bleaching/UV-B-100<br>10mg/ml in Water (ICN)	none
Porosity		-/- or %	n.a.	non-porous
Pour density		cm3/g	(BJH) Pore Size Distribution and Volume by ASAP2020 (UU)	0.07
Size distribution:<br>(suspension in water)	Modal value X_M (PDI)	nm	Zetasizer Nano ZS /UCD	**H_2O**: 263 (PDI:0.319)/ NM: 191.26<br>**HNO_3**: 1293 (PDI:0.473)/ NM: 818.26<br>**NaOH**: 147.1 (PDI:0.330)/ NM: 34.52
			DCS (UCD) (RWtAv / RNumAv)	n.a.
	Total concentration	mg/ml		1mg/ml
Size distribution:<br>(Aerosol)	Modal value X_M (σ_{geo})	nm	SMPS TSI, DMA 3071 with CPC 3022A / KIT<br>dispersed by electrospray	36.3 (σ_{geo}=1.31)
	Total concentration	mg/cm³ or #/cm³		n.a.
Solubility in _____		g/l		
Solubility in H_2O after 30 days		mg/ml	Extraction/ ICP-MS (ICN)	1.8
Specific surface area		cm²/g	BET by ASAP2020 (UU)	38.8
Surface chemistry				
Zeta potential (surface charge)		eV	Zetasizer Nano ZS /UU	-18.3 ± 6.0mV<br>(pH 4.22)

Summary of trace analyses		ICP-MS / KIT	
Li	µg/g		<0.1
Na	µg/g		5.3
Mg	µg/g		2.1
Al	µg/g		15.3
K	µg/g		<2
Ca	µg/g		9.2
Mn	µg/g		0.12
Fe	µg/g		7.1
Ni	µg/g		<0.5
Cu	µg/g		0.28
Zn	µg/g		2.5

Electron microscopy

Method / technical data of microscope:

SOP: See KIT (LEM)

Magnification: 450.000

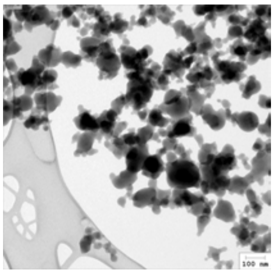

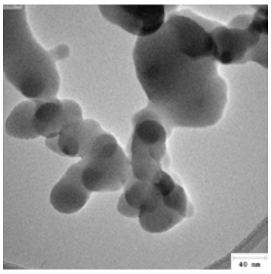

Electron Microscopy by KIT (LEM); Diameter : 20-120 nm

UNILEEDS

TEM/ SEM-EDX

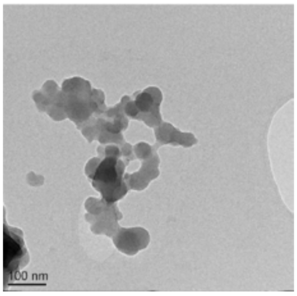

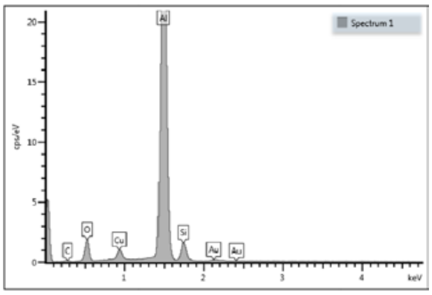

Radical formation potential by DCF-Test:

(H_2O_2 as positive control) (KIT)

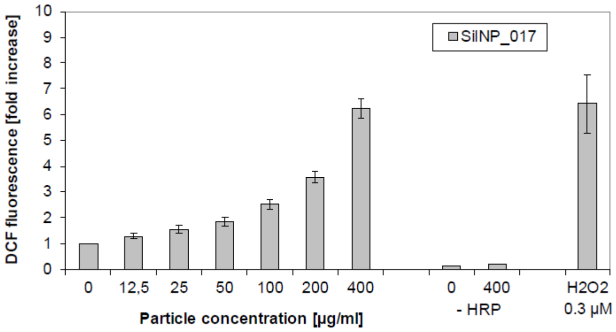

Result: SilNP_017 NPs induced a significant DCFH oxidation at high concentrations indicating a potential to produce reactive oxygen species.

Comments and other relevant physical-chemical properties and material characterisation information

**Place, Date Karlsruhe, 29.11.2016**	**Responsible H.-R. Paur, KIT**

Supplemental S4: Physicochemical characterization data sheets of zinc oxide NMs

**REPRESENTATIVE TEST PARTICLES**<br>Nanomaterial Identification<br>according to OECD<br>ENV/JM/MONO(2009)20/REV pp29.	Research Infrastructure QualityNano
Nanomaterial name: ZnO NPs B1-1<br>Particle Code: ZnO_NP001<br>Composition: ZnO<br>Method of production: B2B Synthesis	**Manufacturer /Institute/Date**<br>**Yunhong Jiang; provided by Univleeds; 03/2012**<br>**Technology Expert:** Yunhong Jiang
**Kind of suspension:**	**Suspension** ☐ **Powder** ☒<br>Suspended in<br>pH ____________<br>stabilizer none_________

**Property**		**unit**	**Method / Institute**	**Value**
Agglomeration/aggregation			HRTEM (KIT)	crystalline
Crystalline phase			D5000 Diffractometer (UU)<br>PANalytical X´Pert diffractometer (ICN)	Amorphous<br>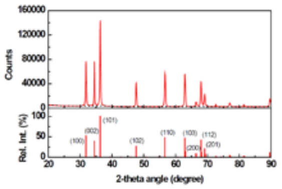
Crystallite size			HRTEM (KIT)	
Octanol-water partition coefficient			Extraction/ ICP-MS (ICN)	P_{OW} = 0
Photocatalytic activity			Rhodamin-B Bleaching/UV-B-100 10mg/ml in Water (ICN)	high
Porosity		-/- or %	n.a.	
Pour density		cm3/g	(BJH) Pore Size Distribution and Volume by ASAP2020 (UU)	n.a.
Size distribution: (suspension in water)	Modal value X_M (PDI)	nm	Zetasizer Nano ZS /UCD<br>DCS (UCD) (RWtAv / RNumAv)	656 (PDI:0.336)/ NM: 426.3<br>n.a.
	Total concentration	mg/ml		1mg/ml
Size distribution: (Aerosol)	Modal value X_M (σ_{geo})	nm	SMPS TSI, DMA 3071 with CPC 3022A / KIT<br>dispersed by electrospray	n.a.
Solubility in _____		g/l		
Solubility in H_2O after 30 days		mg/ml	Extraction/ ICP-MS (ICN)	7
Specific surface area		cm²/g	BET by ASAP2020 (UU)	n.a.
Surface chemistry				
Zeta potential (surface charge)		eV	Zetasizer Nano ZS /UU	+13.0 ± 3.3 mV<br>pH: 6.05

Summary of trace analyses		ICP-MS (KIT)	Assay1	Assay2
Na	µg/g		7.0	6.4
Mg	µg/g		2.5	<2.0
Al	µg/g		3.6	2.9
Ca	µg/g		7.2	6.0
Fe	µg/g		7.7	8.0
Ni	µg/g		1.7	1.9
Cu	µg/g		1.8	1.9
Sr	µg/g		22.0	23.3
Cd	µg/g		3.5	3.7
Ba	µg/g		0.1	0.1
Tl	µg/g		1.5	1.5
Pb	µg/g		11.4	11.3

Electron microscopy

Method / technical data of microscope:

SOP: See KIT (LEM)

Magnification: 450.000

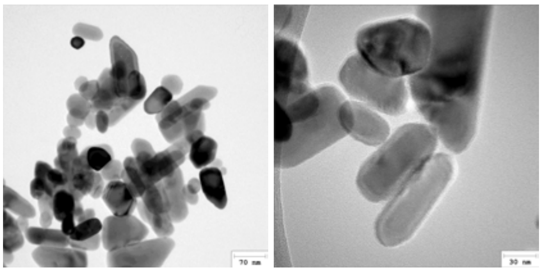

Electron Microscopy by KIT (LEM); Diameter : 30-200 nm

UNIVLEEDS

TEM/ TEM-EDX

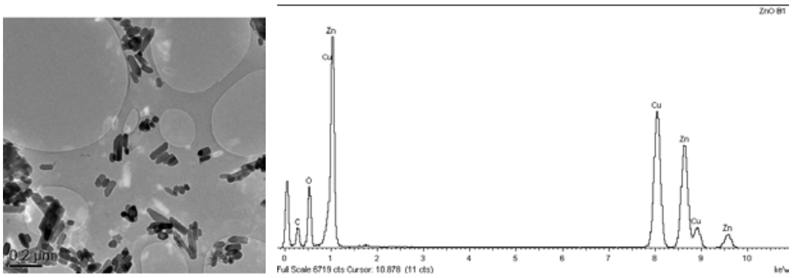

ZnO NPs B1-1

Radical formation potential by DCF-Test:

(H_2O_2 as positive control) (KIT)

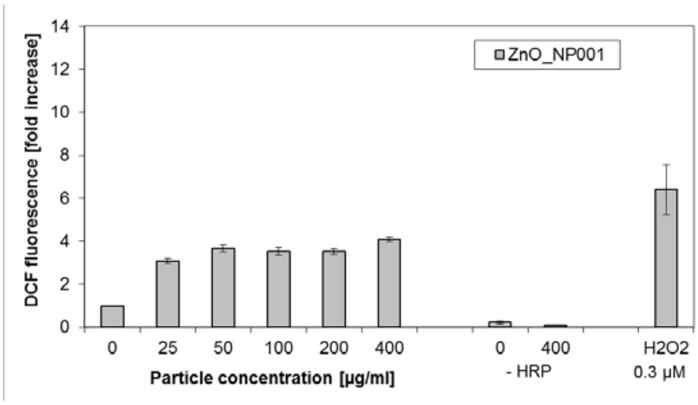

Result: ZnO_NP001 induced a moderate increase of DCFH oxidation at concentrations from 25 to 400 µg/ml.

Comments and other relevant physical-chemical properties and material characterization information

Place, Date Karlsruhe, 29.11.2016	Responsible H.-R. Paur, KIT

REPRESENTATIVE TEST PARTICLES

Nanomaterial Identification
according to OECD
ENV/JM/MONO(2009)20/REV pp29.

Research Infrastructure QualityNano

Nanomaterial name	ZnO NPs B 1-2	**Manufacturer /Institute/Date**
Particle Code	ZnO_NP002	**Yunhong Jiang; provided by Univleeds; 03/2012**
Composition	ZnO	**Technology Expert:** Yunhong Jiang
Method of production	B2B Synthesis	

Kind of suspension:

Suspension ☐ **Powder** ☒

Suspended in
pH ____________
stabilizer none_________

**Property**		**unit**	**Method / Institute**	**Value**
Agglomeration/aggregation			HRTEM (KIT)	crystalline
Crystalline phase			D5000 Diffractometer (UU)<br>PANalytical X´Pert diffractometer (ICN)	Amorphous<br>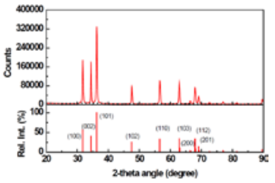
Crystallite size			HRTEM (KIT)	
Octanol-water partition coefficient			Extraction/ ICP-MS (ICN)	P_{OW}=0
Photocatalytic activity			Rhodamin-B Bleaching/UV-B-100<br>10mg/ml in Water (ICN)	high
Porosity		-/- or %	n.a.	
Pour density		cm3/g	(BJH) Pore Size Distribution and Volume by ASAP2020 (UU)	
Size distribution:<br>(suspension in water)	Modal value X_M (PDI)	nm	Zetasizer Nano ZS /UCD<br>UNIVLEEDS<br>DCS (UCD) (RWtAv / RNumAv)	321,9 (PDI:0,293)/ NM: 181,5<br>179,5 (PDI:0,502)
	Total concentration	mg/ml		1mg/ml
Size distribution:<br>(Aerosol)	Modal value X_M (σ_{geo})	nm	SMPS TSI, DMA 3071 with CPC 3022A / KIT<br>dispersed by electrospray	
Solubility in _____		g/l		
Solubility in H_2O after 30 days		mg/ml	Extraction/ ICP-MS (ICN)	4.5
Specific surface area		cm²/g	BET by ASAP2020 (UU)	
Surface chemistry				
Zeta potential (surface charge)		eV	Zetasizer Nano ZS /UU	-10.0 ± 1.6 mV<br>pH: 5.89

Summary of trace analyses		ICP-MS (KIT)	Assay1	Assay2
Na	µg/g		55.1	27.6
Mg	µg/g			2.6
Al	µg/g		5.4	7.6
Ca	µg/g		14.3	14.0
Fe	µg/g		11.4	11.6
Ni	µg/g		2.9	2.4
Cu	µg/g		2.1	2.4
Sr	µg/g		27.4	26.6
Cd	µg/g		4.3	
Ba	µg/g		0.2	0.2
Tl	µg/g		2.9	2.8
Pb	µg/g		16.7	15.7

Electron microscopy
Method / technical data of microscope:
SOP: See KIT (LEM)

Magnification: 450.000

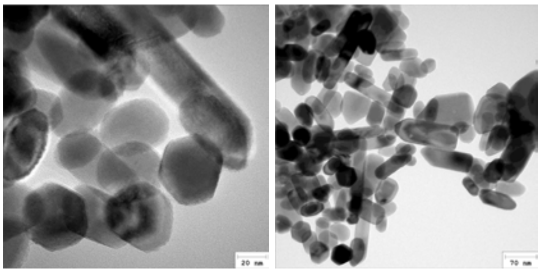

Electron Microscopy by KIT (LEM); Diameter : 30-200 nm

UNIVLEEDS

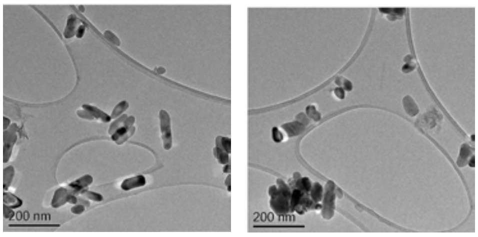

ZnO NPs B 1-2

Radical formation potential by DCF-Test:
(H_2O_2 as positive control) (KIT)

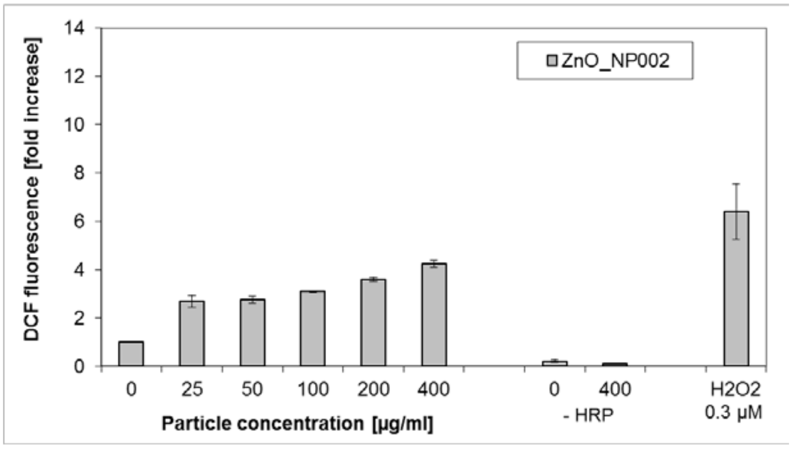

Result: ZnO_NP002 induced a moderate increase of DCFH oxidation at concentrations from 25 to 400 µg/ml.

Comments and other relevant physical-chemical properties and material characterization information

Place, Date Karlsruhe, 06.03.2013	Responsible H.-R. Paur, KIT

REPRESENTATIVE TEST PARTICLES
Nanomaterial Identification
according to OECD
ENV/JM/MONO(2009)20/REV pp29.

Research Infrastructure QualityNano

Nanomaterial name	ZnO NPs B 1-3	**Manufacturer /Institute/Date**	
Particle Code	ZnO_NP003	**Yunhong Jiang; provided by Univleeds;**	**03/2012**
Composition	ZnO	**Technology Expert:** Yunhong Jiang	
Method of production	B2B Synthesis		

Kind of suspension:

Suspension ☐ **Powder** ☒

Suspended in

pH ____________

stabilizer none_________

**Property**		**unit**	**Method / Institute**	**Value**
Agglomeration/aggregation			HRTEM (KIT)	crystalline
Crystalline phase			D5000 Diffractometer (UU)<br>PANalytical X´Pert diffractometer (ICN)	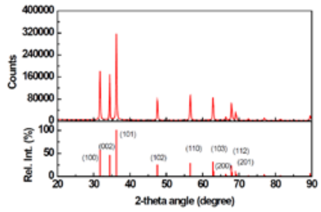
Crystallite size			HRTEM (KIT)	
Octanol-water partition coefficient			Extraction/ ICP-MS (ICN)	P_{OW}=0
Photocatalytic activity			Rhodamin-B Bleaching/UV-B-100<br>10mg/ml in Water (ICN)	high
Porosity		-/- or %	n.a.	
Pour density		cm3/g	(BJH) Pore Size Distribution and Volume by ASAP2020 (UU)	
Size distribution:<br>(suspension in water)	Modal value X_M (PDI)	nm	Zetasizer Nano ZS /UCD<br>UNIVLEEDS<br>DCS (UCD) (RWtAv / RNumAv)	203,8 (PDI:0,208)/ NM: 159,8<br>160,1(PDI:0,564)
	Total concentration	mg/ml		1mg/ml
Size distribution:<br>(Aerosol)	Modal value X_M (σ_{geo})	nm	SMPS TSI, DMA 3071 with CPC 3022A / KIT<br>dispersed by electrospray	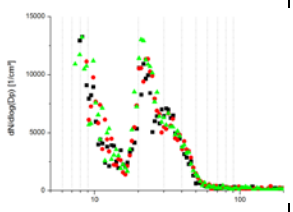
Solubility in _____		g/l		
Solubility in H_2O after 30 days		mg/ml	Extraction/ ICP-MS (ICN)	Ca. 7.4
Specific surface area		cm²/g	BET by ASAP2020 (UU)	
Surface chemistry				
Zeta potential (surface charge)		eV	Zetasizer Nano ZS /UU	-15.6 ± 2.3 mV<br>pH: 5.97

Summary of trace analyses		ICP-MS / KIT	Assay1	Assay2
Na	µg/g		74.7	80.1
Mg	µg/g		21.4	20.8
Al	µg/g		5.0	6.6
K	µg/g		10.4	11.4
Ca	µg/g		135.5	137
Ti	µg/g		0.2	0.1
Mn	µg/g		0.3	<0.2
Fe	µg/g		25.9	8.3
Ni	µg/g		2.5	0.5
Cu	µg/g		3.6	3.8
Sr	µg/g		60.4	61.2
Cd	µg/g		5.5	5.6
Ba	µg/g		0.2	0.2
Tl	µg/g		3.4	3.6
Pb	µg/g		18.7	19.7

Electron microscopy
Method / technical data of microscope:
SOP: See KIT (LEM)

Magnification: 450.000

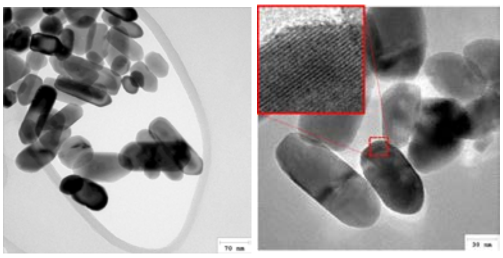

Electron Microscopy by KIT (LEM); Diameter : 30-200 nm

UNIVLEEDS

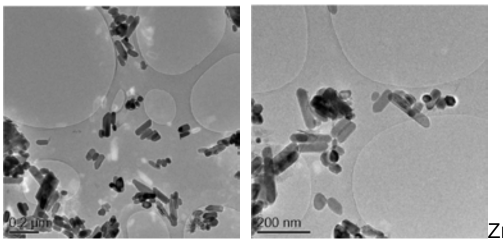

ZnO NPs B 1-3

Radical formation potential by DCF-Test:
(H_2O_2 as positive control) (KIT)

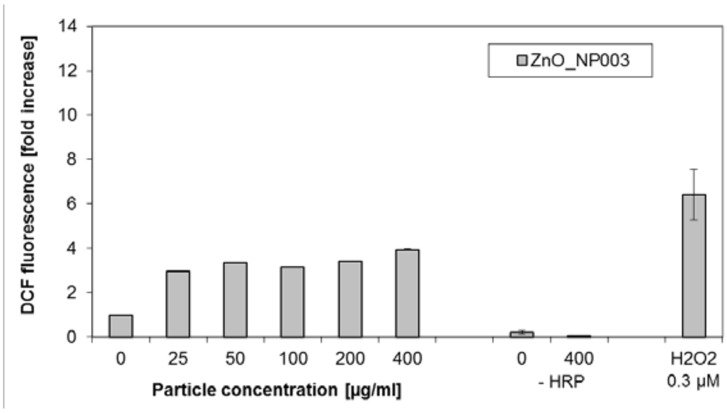

Result: ZnO_NP003 induced a moderate increase of DCFH oxidation at concentrations from 25 to 400 µg/ml.

Comments and other relevant physical-chemical properties and material characterization information

Place, Date Karlsruhe, 06.03.2013

Responsible H.-R. Paur, KIT

REPRESENTATIVE TEST PARTICLES

Nanomaterial Identification
according to OECD
ENV/JM/MONO(2009)20/REV pp29.

Research Infrastructure QualityNano

Nanomaterial name	ZnO NPs B 2-1
Particle Code	ZnO_NP004
Composition	ZnO
Method of production	B2B Synthesis

Manufacturer /Institute/Date

Yunhong Jiang; provided by Univleeds; 03/2012

Technology Expert: Yunhong Jiang

Kind of suspension:

Suspension ☐ **Powder** ☒

Suspended in

pH ____________

stabilizer none_________

Property	unit	Method / Institute	Value
Agglomeration/aggregation		HRTEM (KIT)	crystalline
Crystalline phase		D5000 Diffractometer (UU)<br>PANalytical X´Pert diffractometer (ICN)	Amorphous<br>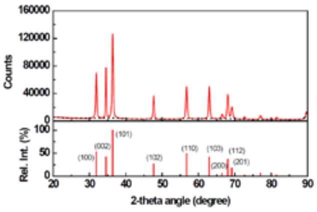
Crystallite size		HRTEM (KIT)	
Octanol-water partition coefficient		Extraction/ ICP-MS (ICN)	P_{OW}=0
Photocatalytic activity		Rhodamin-B Bleaching/UV-B-100<br>10mg/ml in Water (ICN)	high
Porosity	-/- or %	n.a.	
Pour density	cm3/g	(BJH) Pore Size Distribution and Volume by ASAP2020 (UU)	n.a.
Size distribution: (suspension in water) — Modal value X_M (PDI)	nm	Zetasizer Nano ZS /UCD<br>DCS (UCD) (RWtAv / RNumAv)	201.5 (PDI:0.212)/ NM: 117.8
Size distribution: (suspension in water) — Total concentration	mg/ml		1mg/ml
Size distribution: (Aerosol) — Modal value X_M (σ_{geo})	nm	SMPS TSI, DMA 3071 with CPC 3022A / KIT<br>dispersed by electrospray	n.a.
Solubility in _____	g/l		
Solubility in H_2O after 30 days	mg/ml	Extraction/ ICP-MS (ICN)	5
Specific surface area	cm²/g	BET by ASAP2020 (UU)	n.a.
Surface chemistry			
Zeta potential (surface charge)	eV	Zetasizer Nano ZS (UU)	+23.0 ± 4.9 mV<br>pH: 6.23

Summary of trace analyses		ICP-MS (KIT)	
Li	µg/g		0.2
Na	µg/g		16.4
Mg	µg/g		5.5
Al	µg/g		5.5
Ca	µg/g		13.8
Ti	µg/g		3.2
Mn	µg/g		0.2
Fe	µg/g		35.4
Ni	µg/g		0.9
Cu	µg/g		2.8
Sr	µg/g		19.1
Mo	µg/g		1.1
Cd	µg/g		4.4
Ba	µg/g		0.2
Tl	µg/g		2.1
Pb	µg/g		32.5

Electron microscopy

Method / technical data of microscope:

SOP: See KIT (LEM)

Magnification: 450.000

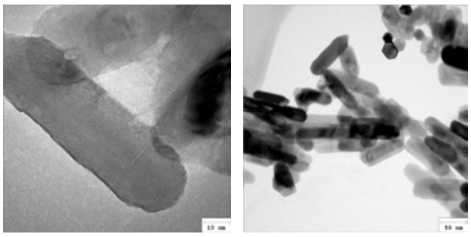

Electron Microscopy by KIT (LEM); Diameter : 30-200 nm

UNIVLEEDS

TEM/ TEM-EDX

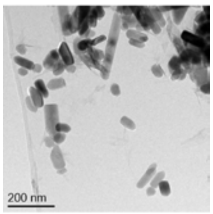

ZnO NPs B 2-1

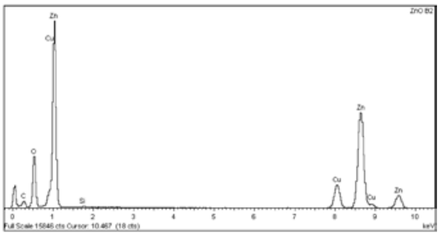

Radical formation potential by DCF-Test:

(H_2O_2 as positive control) (KIT)

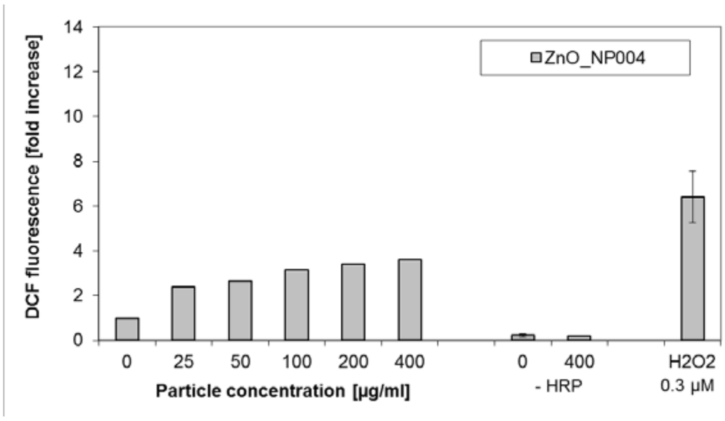

Result: ZnO_NP004 induced a moderate increase of DCFH oxidation at concentrations from 25 to 400 µg/ml.

Comments and other relevant physical-chemical properties and material characterisation information

**Place, Date Karlsruhe, 29.11.2016**	**Responsible H.-R. Paur, KIT**

REPRESENTATIVE TEST PARTICLES
Nanomaterial Identification
according to OECD
ENV/JM/MONO(2009)20/REV pp29.

Research Infrastructure QualityNano

Nanomaterial name	ZnO NPs B 2-2
Particle Code	ZnO_NP005
Composition	ZnO
Method of production	B2B Synthesis

Manufacturer /Institute/Date

Yunhong Jiang; provided by Univleeds; 03/2012

Technology Expert: Yunhong Jiang

Kind of suspension:

Suspension ☐ **Powder** ☒

Suspended in
pH ____________
stabilizer none_________

**Property**		**unit**	**Method / Institute**	**Value**
Agglomeration/aggregation			HRTEM (KIT)	crystalline
Crystalline phase			D5000 Diffractometer (UU)<br>PANalytical X´Pert diffractometer (ICN)	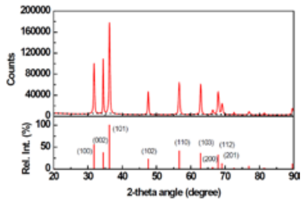
Crystallite size			HRTEM (KIT)	
Octanol-water partition coefficient			Extraction/ ICP-MS (ICN)	P_{OW}=0
Photocatalytic activity			Rhodamin-B Bleaching/UV-B-100<br>10mg/ml in Water (ICN)	high
Porosity		-/- or %	n.a.	
Pour density		cm3/g	(BJH) Pore Size Distribution and Volume by ASAP2020 (UU)	
Size distribution:<br>(suspension in water)	Modal value X_M (PDI)	nm	Zetasizer Nano ZS /UCD<br>UNIVLEEDS<br>DCS (UCD) (RWtAv / RNumAv)	606,2 (PDI:0,308)/ NM: 117,8<br>511,8 (PDI:0,297)
	Total concentration	mg/ml		1mg/ml
Size distribution:<br>(Aerosol)	Modal value X_M (σ_{geo})	nm	SMPS TSI, DMA 3071 with CPC 3022A / KIT<br>dispersed by electrospray	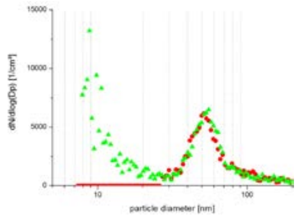
Solubility in _____		g/l		
Solubility in H_2O after 30 days		mg/ml	Extraction/ ICP-MS (ICN)	4.5
Specific surface area		cm²/g	BET by ASAP2020 (UU)	
Surface chemistry				
Zeta potential (surface charge)		eV	Zetasizer Nano ZS (UU)	+20.3 ± 4.2 mV<br>pH: 6.28

Summary of trace analyses		ICP-MS (KIT)	
Na	µg/g		16.3
Mg	µg/g		5.3
Al	µg/g		4.7
Ca	µg/g		14.5
Ti	µg/g		3.2
Mn	µg/g		10.4
Fe	µg/g		1080.0
Ni	µg/g		1.1
Cu	µg/g		3.0
Sr	µg/g		20.1
Mo	µg/g		1.2
Cd	µg/g		4.6
Ba	µg/g		0.2
Tl	µg/g		2.0
Pb	µg/g		35.1

Electron microscopy
Method / technical data of microscope:
SOP: See KIT (LEM)

Magnification: 450.000

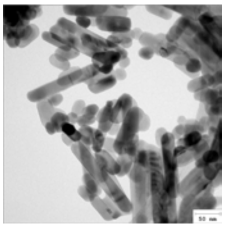

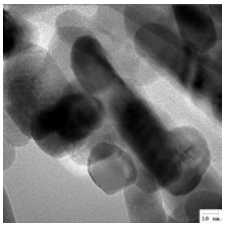

Electron Microscopy by KIT (LEM); Diameter : 30-300 nm

UNIVLEEDS

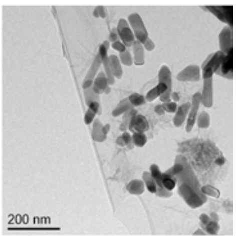

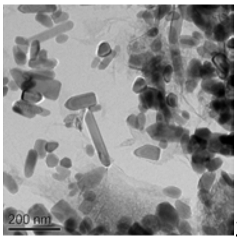

ZnO NPs B 2-2

Radical formation potential by DCF-Test:
(H_2O_2 as positive control) (KIT)

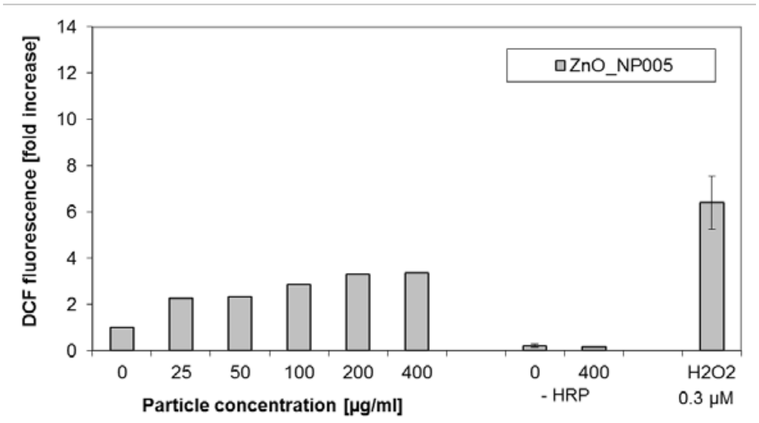

Result: ZnO_NP005 induced a moderate increase of DCFH oxidation at concentrations from 25 to 400 µg/ml.

Comments and other relevant physical-chemical properties and material characterization information

**Place, Date Karlsruhe, 06.03.2013**	**Responsible H.-R. Paur, KIT**

REPRESENTATIVE TEST PARTICLES
Nanomaterial Identification
according to OECD
ENV/JM/MONO(2009)20/REV pp29.

Research Infrastructure QualityNano

Nanomaterial name	ZnO NPs B 2-3	**Manufacturer /Institute/Date**
Particle Code	ZnO_NP006	**Yunhong Jiang; provided by Univleeds; 03/2012**
Composition	ZnO	**Technology Expert:** Yunhong Jiang
Method of production	B2B Synthesis	

Kind of suspension:

Suspension ☐ **Powder** ☒

Suspended in
pH ____________
stabilizer none_________

Property		unit	Method / Institute	Value
Agglomeration/aggregation			HRTEM (KIT)	crystalline
Crystalline phase			D5000 Diffractometer (UU)<br>PANalytical X´Pert diffractometer (ICN)	Amorphous<br>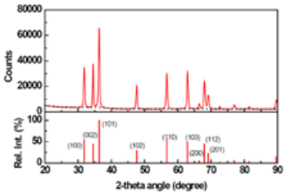
Crystallite size			HRTEM (KIT)	
Octanol-water partition coefficient			Extraction/ ICP-MS (ICN)	P_{OW}=0
Photocatalytic activity			Rhodamin-B Bleaching/UV-B-100<br>10mg/ml in Water (ICN)	high
Porosity		-/- or %	n.a.	
Pour density		cm3/g	(BJH) Pore Size Distribution and Volume by ASAP2020 (UU)	
Size distribution:<br>(suspension in water)	Modal value X_M (PDI)	nm	Zetasizer Nano ZS /UCD<br>UNIVLEEDS<br>DCS (UCD) (RWtAv / RNumAv)	675,2 (PDI:0,305)/ NM: 117,8<br>563,8 (PDI:0,432)
	Total concentration	mg/ml		1mg/ml
Size distribution:<br>(Aerosol)	Modal value X_M (σ_{geo})	nm	SMPS TSI, DMA 3071 with CPC 3022A / KIT<br>dispersed by electrospray	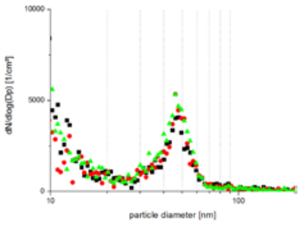
Solubility in _____		g/l		
Solubility in H_2O after 30 days		mg/ml	Extraction/ ICP-MS (ICN)	Ca.5
Specific surface area		cm²/g	BET by ASAP2020 (UU)	
Surface chemistry				
Zeta potential (surface charge)		eV	Zetasizer Nano ZS (UU)	+20.9 ± 3.6 mV<br>pH: 6.26

Summary of trace analyses		ICP-MS (KIT)	Assay1	Assay2
Li	µg/g		0.4	0.2
Na	µg/g		17.7	16.0
Mg	µg/g		12.2	5.4
Al	µg/g		8.7	4.8
Ca	µg/g		16.8	15.2
Ti	µg/g		3.9	2.9
Mn	µg/g			0.2
Fe	µg/g		36.6	34.8
Ni	µg/g		1.0	1.0
Cu	µg/g		2.8	2.9
Sr	µg/g		20.8	20.2
Mo	µg/g		1.1	1.1
Cd	µg/g		4.5	4.6
Ba	µg/g		0.2	0.2
Tl	µg/g		2.2	2.3
Pb	µg/g		35.0	34.2

Electron microscopy
Method / technical data of microscope:
SOP: See KIT (LEM)

Magnification: 450.000

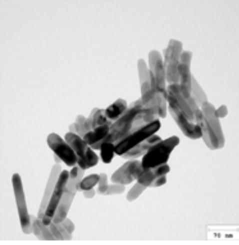
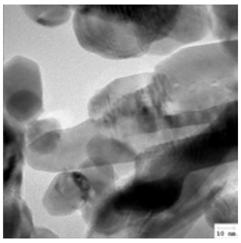

Electron Microscopy by KIT (LEM); Diameter : 30-200 nm

UNIVLEEDS

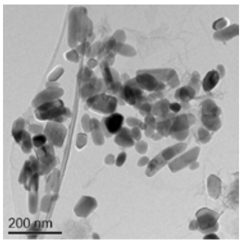

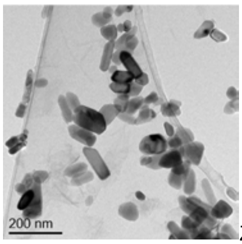

ZnO NPs B 2-3

Radical formation potential by DCF-Test:	Not analyzed.

Comments and other relevant physical-chemical properties and material characterization information

**Place, Date Karlsruhe, 06.03.2013**	**Responsible H.-R. Paur, KIT**

**REPRESENTATIVE TEST PARTICLES**<br>Nanomaterial Identification<br>according to OECD<br>ENV/JM/MONO(2009)20/REV pp29.	Research Infrastructure QualityNano
Nanomaterial name: ZnO NPs B 3-1<br>Particle Code: ZnO_NP007<br>Composition: ZnO<br>Method of production: B2B Synthesis	**Manufacturer /Institute/Date**<br>**Yunhong Jiang; provided by Univleeds; 03/2012**<br>**Technology Expert:** Yunhong Jiang
**Kind of suspension:**	**Suspension** ☐ **Powder** ☒<br>Suspended in<br>pH ____________<br>stabilizer none_________

**Property**		**unit**	**Method / Institute**	**Value**
Agglomeration/aggregation			HRTEM (KIT)	crystalline
Crystalline phase			D5000 Diffractometer (UU)<br>PANalytical X´Pert diffractometer (ICN)	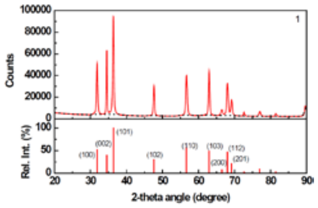
Crystallite size			HRTEM (KIT)	
Octanol-water partition coefficient			Extraction/ ICP-MS (ICN)	P_{OW}=0
Photocatalytic activity			Rhodamin-B Bleaching/UV-B-100<br>10mg/ml in Water (ICN)	high
Porosity		-/- or %	n.a.	
Pour density		cm3/g	(BJH) Pore Size Distribution and Volume by ASAP2020 (UU)	
Size distribution:<br>(suspension in water)	Modal value X_M (PDI)	nm	Zetasizer Nano ZS /UCD<br>UNIVLEEDS<br>DCS (UCD) (RWtAv / RNumAv)	647.9 (PDI:0.313)/ NM: 445.7<br>131.0 PDI(:0.447)
	Total concentration	mg/ml		1mg/ml
Size distribution:<br>(Aerosol)	Modal value X_M (σ_{geo})	nm	SMPS TSI. DMA 3071 with CPC 3022A / KIT<br>dispersed by electrospray	
Solubility in _____		g/l		
Solubility in H_2O after 30 days		mg/ml	Extraction/ ICP-MS (ICN)	7.5
Specific surface area		cm²/g	BET by ASAP2020 (UU)	
Surface chemistry				
Zeta potential (surface charge)		eV	Zetasizer Nano ZS /UU	-5.9 ± 4.2 mV<br>pH: 6.28

Summary of trace analyses		ICP-MS / KIT	
Na	µg/g		27.3
Al	µg/g		4.6
Ca	µg/g		4.8
Ti	µg/g		0.4
Fe	µg/g		30.8
Co	µg/g		0.3
Ni	µg/g		6.6
Cu	µg/g		2.4
Cd	µg/g		<0.1
Pb	µg/g		4.9

Electron microscopy
Method / technical data of microscope:
SOP: See KIT (LEM)

Magnification: 450.000

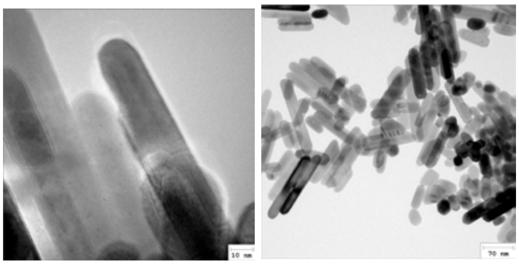

Electron Microscopy by KIT (LEM); Diameter : 30-200 nm

UNIVLEEDS

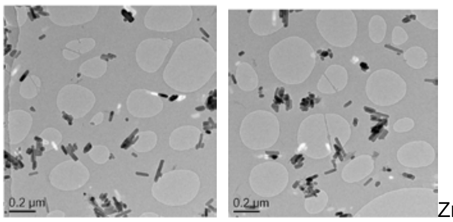

ZnO NPs B 3-1

TEM EDX
UNIVLEEDS

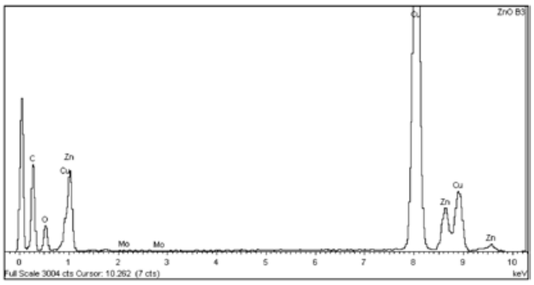

Radical formation potential by DCF-Test:
(H_2O_2 as positive control) (KIT)

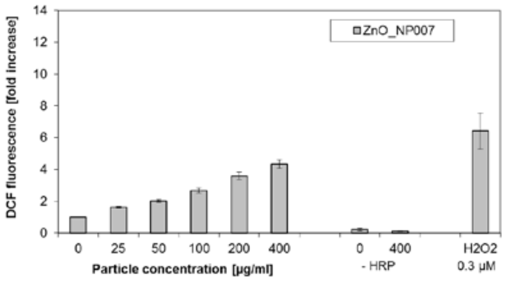

Result: ZnO_NP007 induced a moderate increase of DCFH oxidation at concentrations from 25 to 400 µg/ml.

Comments and other relevant physical-chemical properties and material characterization information

**Place, Date Karlsruhe, 06.03.2013**	**Responsible H.-R. Paur, KIT**

REPRESENTATIVE TEST PARTICLES

Nanomaterial Identification
according to OECD
ENV/JM/MONO(2009)20/REV pp29.

Research Infrastructure QualityNano

Nanomaterial name	ZnO NPs B 3-2
Particle Code	ZnO_NP008
Composition	ZnO
Method of production	B2B Synthesis

Manufacturer /Institute/Date

Yunhong Jiang; provided by Univleeds; 03/2012

Technology Expert: Yunhong Jiang

Kind of suspension:

Suspension ☐ **Powder** ☒

Suspended in

pH ____________

stabilizer none_________

Property	unit	Method / Institute	Value
Agglomeration/aggregation		HRTEM (KIT)	crystalline
Crystalline phase		D5000 Diffractometer (UU)<br>PANalytical X´Pert diffractometer (ICN)	Amorphous<br>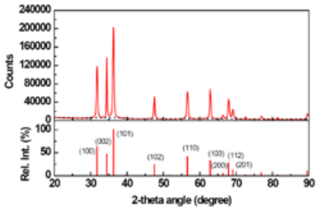
Crystallite size		HRTEM (KIT)	
Octanol-water partition coefficient		Extraction/ ICP-MS (ICN)	P_{OW}=0
Photocatalytic activity		Rhodamin-B Bleaching/UV-B-100<br>10mg/ml in Water (ICN)	high
Porosity	-/- or %	n.a.	
Pour density	cm3/g	(BJH) Pore Size Distribution and Volume by ASAP2020 (UU)	
Size distribution: (suspension in water) — Modal value X_M (PDI)	nm	Zetasizer Nano ZS /UCD<br>UNIVLEEDS<br>DCS (UCD) (RWtAv / RNumAv)	670.1 (PDI: 0.331)<br>/ NM: 343.6<br>130.2 (PDI: 0.492)
Size distribution: (suspension in water) — Total concentration	mg/ml		1mg/ml
Size distribution: (Aerosol) — Modal value X_M (σ_{geo})	nm	SMPS TSI, DMA 3071 with CPC 3022A / KIT<br>dispersed by electrospray	
Solubility in _____	g/l		
Solubility in H_2O after 30 days	mg/ml	Extraction/ ICP-MS (ICN)	6.5
Specific surface area	cm²/g	BET by ASAP2020 (UU)	
Surface chemistry			
Zeta potential (surface charge)	eV	Zetasizer Nano ZS /UU	+24.3 ± 3.3 mV<br>pH: 6.43

Summary of trace analyses		ICP-MS / KIT	
Na	µg/g		27.2
Mg	µg/g		18.1
Al	µg/g		7.3
Ca	µg/g		3.7
Ti	µg/g		0.4
Fe	µg/g		30.7
Co	µg/g		0.3
Ni	µg/g		3.2
Cu	µg/g		2.5
Cd	µg/g		<0.1
Pb	µg/g		4.8

Electron microscopy

Method / technical data of microscope:

SOP: See KIT (LEM)

Magnification: 450.000

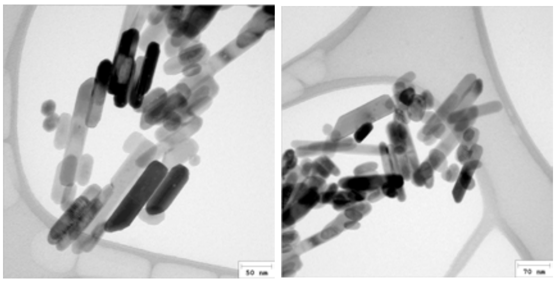

Electron Microscopy by KIT (LEM); Diameter : 30-200 nm

UNIVLEEDS

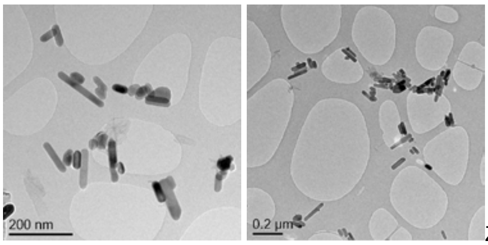

ZnO NPs B 3-2

Radical formation potential by DCF-Test:

(H_2O_2 as positive control) (KIT)

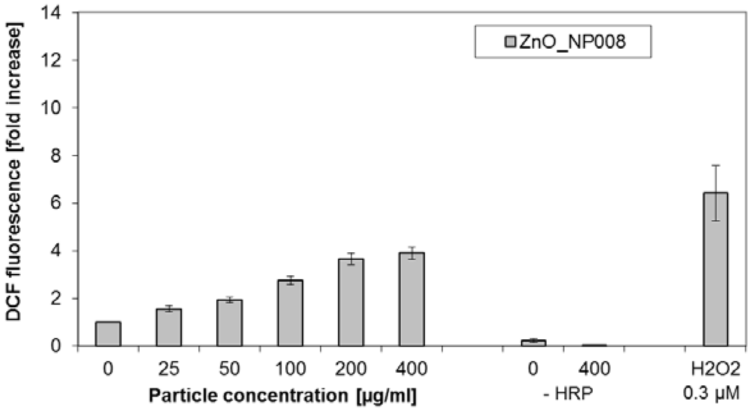

Result: ZnO_NP008 induced a moderate increase of DCFH oxidation at concentrations from 25 to 400 µg/ml.

Comments and other relevant physical-chemical properties and material characterization information

Place, Date Karlsruhe, 06.03.2013	Responsible H.-R. Paur, KIT

REPRESENTATIVE TEST PARTICLES
Nanomaterial Identification
according to OECD
ENV/JM/MONO(2009)20/REV pp29.

Research Infrastructure QualityNano

Nanomaterial name	ZnO NPs B 3-3
Particle Code	ZnO_NP009
Composition	ZnO
Method of production	B2B Synthesis

Manufacturer /Institute/Date

Yunhong Jiang; provided by Univleeds; 03/2012

Technology Expert: Yunhong Jiang

Kind of suspension:

Suspension [ ] **Powder** [x]

Suspended in
pH ____________
stabilizer none_________

Property	unit	Method / Institute	Value
Agglomeration/aggregation		HRTEM (KIT)	crystalline
Crystalline phase		D5000 Diffractometer (UU)<br>PANalytical X´Pert diffractometer (ICN)	Amorphous<br>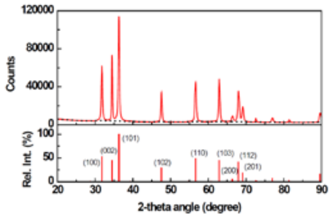
Crystallite size		HRTEM (KIT)	
Octanol-water partition coefficient		Extraction/ ICP-MS (ICN)	P_{OW}=0
Photocatalytic activity		Rhodamin-B Bleaching/UV-B-100<br>10mg/ml in Water (ICN)	high
Porosity	-/- or %	n.a.	
Pour density	cm3/g	(BJH) Pore Size Distribution and Volume by ASAP2020 (UU)	
Size distribution: (suspension in water) — Modal value X_M (PDI)	nm	Zetasizer Nano ZS /UCD<br>UNIVLEEDS<br>DCS (UCD) (RWtAv / RNumAv)	657.7 (PDI:0.311)/ NM: 422.7<br>132.6 (PDI:0.573)
Size distribution: (suspension in water) — Total concentration	mg/ml		1mg/ml
Size distribution: (Aerosol) — Modal value X_M (σ_{geo})	nm	SMPS TSI. DMA 3071 with CPC 3022A / KIT<br>dispersed by electrospray	
Solubility in _____	g/l		
Solubility in H_2O after 30 days	mg/ml	Extraction/ ICP-MS (ICN)	7.5
Specific surface area	cm²/g	BET by ASAP2020 (UU)	
Surface chemistry			
Zeta potential (surface charge)	eV	Zetasizer Nano ZS /UU	+21.7 ± 3.6 mV<br>pH: 6.46

Summary of trace analyses		ICP-MS (KIT)	
Na	µg/g		29.3
Al	µg/g		8.5
Ca	µg/g		3.1
Ti	µg/g		0.5
Fe	µg/g		30.8
Co	µg/g		0.3
Ni	µg/g		5.3
Cu	µg/g		2.7
Cd	µg/g		<0.1
Pb	µg/g		6.4
**Electron microscopy**<br>Method / technical data of microscope:<br>SOP: See KIT (LEM)<br><br>Magnification: 450.000<br><br>UNIVLEEDS	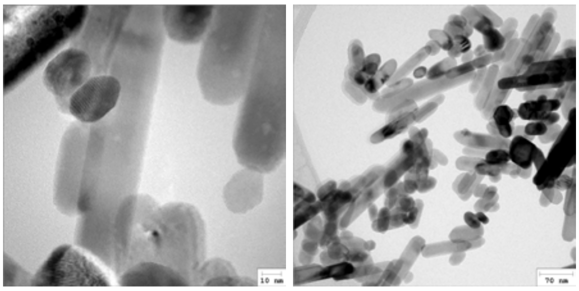<br>Electron Microscopy by KIT (LEM); Diameter : 30-200 nm<br>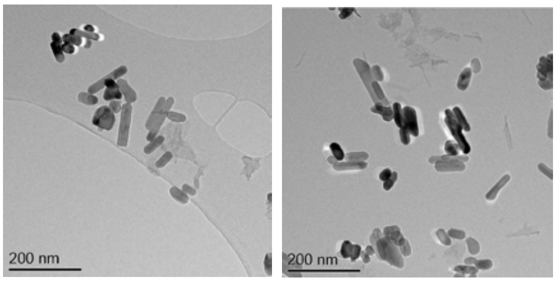<br>ZnO NPs B 3-3		
Radical formation potential by DCF-Test:<br>(H_2O_2 as positive control) (KIT)	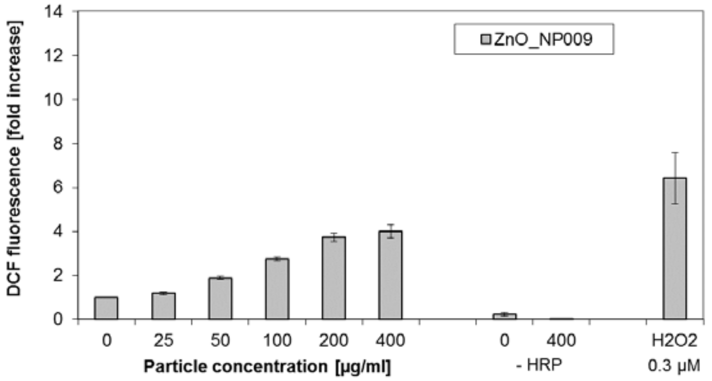<br>Result: ZnO_NP009 induced a moderate increase of DCFH oxidation at concentrations from 25 to 400 µg/ml.		
**Comments and other relevant physical-chemical properties and material characterization information**			
**Place, Date Karlsruhe, 06.03.2013**	**Responsible H.-R. Paur. KIT**		

Supplemental S5: Physicochemical characterization data sheets of titania NMs

**REPRESENTATIVE TEST PARTICLES**<br>Nanomaterial Identification<br>according to OECD<br>ENV/JM/MONO(2009)20/REV pp29.	Research Infrastructure QualityNano
Nanomaterial name: TiO_2-NP<br>Particle Code: TiO_2NP_001<br>Composition: TiO_2<br>Method of production: B2B Sol-Gel synthesis	**Manufacturer /Institute/Date**<br>**Jordi Piella Bagaria; provided by ICN; 09/2012**<br>**Technology Expert:** Jordi Piella Bagaria
**Kind of suspension:**	**Suspension** ☒ **Powder** ☐<br>Suspended in: miliQ-water<br>pH: 3<br>stabilizer: 5 mM TMAOH

**Property**		**unit**	**Method / Institute**	**Value**
Agglomeration/aggregation			HRTEM (KIT)	Crystalline/ Agglomerated
Crystalline phase			D5000 Diffractometer (UU)<br>PANalytical X´Pert diffractometer (ICN)	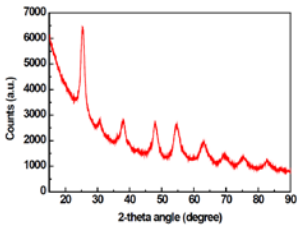
Crystallite size			HRTEM (KIT)	
Octanol-water partition coefficient			Extraction/ ICP-MS (ICN)	P_{OW}=0
Photocatalytic activity			Rhodamin-B Bleaching/UV-B-100 10mg/ml in Water (ICN)	high
Porosity		-/- or %	n.a.	n.a.
Pour density		cm3/g	(BJH) Pore Size Distribution and Volume by ASAP2020 (UU)	n.a.
Size distribution: (suspension in water)	Modal value X_M (PDI)	nm	Zetasizer Nano ZS /UCD<br>DCS (UCD) (RWtAv / RNumAv)	59.08 (PDI:0.163)<br>0.0233/0.0230
	Total concentration	mg/ml		1mg/ml
Size distribution: (Aerosol)	Modal value X_M (σ_{geo})	nm	SMPS TSI, DMA 3071 with CPC 3022A (KIT)<br>dispersed by electrospray	36 (σ_{geo}=1.23)
	Total concentration	mg/cm³ or #/cm³		8.9x 10^3/cm^3
Solubility in _____		g/l		
Solubility in H2O after 30 days		mg/ml	Extraction/ ICP-MS (ICN)	none
Specific surface area		cm²/g	BET by ASAP2020 (UU)	n.a.
Surface chemistry				
Zeta potential (surface charge)		eV	Zetasizer Nano ZS /UU	-40.4 ± 1.4 mV<br>pH (10.32)

Summary of trace analyses		ICP-MS / KIT	
Ba	µg/g		16
Ca	µg/g		215
Fe	µg/g		39
K	µg/g		710
Mg	µg/g		10
Mn	µg/g		0.7
Na	µg/g		7700
Nb	µg/g		1.3
Ni	µg/g		25
Pb	µg/g		0.1
Sn	µg/g		10
Zn	µg/g		144

Electron microscopy
Method / technical data of microscope:
SOP: See KIT (LEM)

magnification: 450.000

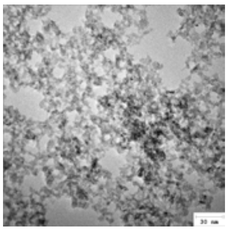

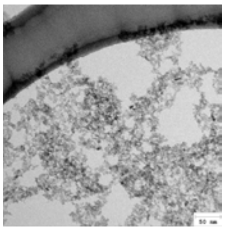

Electron Microscopy by KIT (LEM); Diameter : 10 nm and smaller

UNIVLEEDS
TEM/ SEM

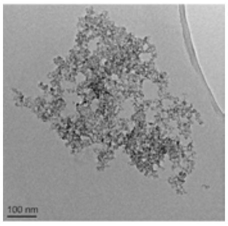

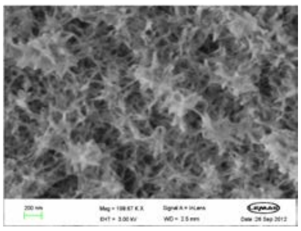

SEM-EDX

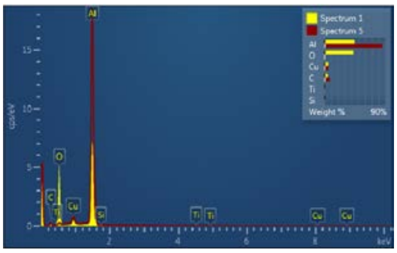

Radical formation potential by DCF-Test:
(H_2O_2 as positive control) (KIT)

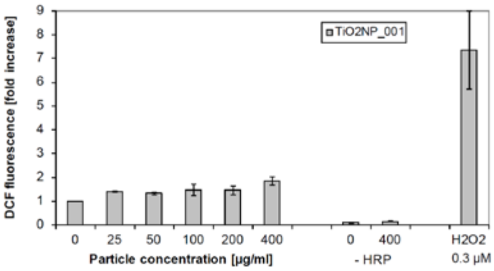

Result: TiO2 NP_001 induced a low increase of DCFH oxidation at high concentration of 400 µg/ml (1.9 fold of nanoparticle free control).

Comments and other relevant physical-chemical properties and material characterization information

Place, Date Karlsruhe, 29.11.2016

Responsible H.-R. Paur, KIT

REPRESENTATIVE TEST PARTICLES
Nanomaterial Identification
according to OECD
ENV/JM/MONO(2009)20/REV pp29.

Research Infrastructure QualityNano

Nanomaterial name	TiO_2-NP
Particle Code	TiO_2NP_002
Composition	TiO_2
Method of production	B2B Sol-Gel synthesis

Manufacturer /Institute/Date

Jordi Piella Bagaria; provided by ICN; 09/2012

Technology Expert: Jordi Piella Bagaria

Kind of suspension:

Suspension ☒ **Powder** ☐

Suspended in	miliQ-water
pH	3
stabilizer	5 mM TMAOH

**Property**		**unit**	**Method / Institute**	**Value**
Agglomeration/aggregation			HRTEM (KIT)	Agglomerated /crystalline
Crystalline phase			D5000 Diffractometer (UU)<br>PANalytical X´Pert diffractometer (ICN)	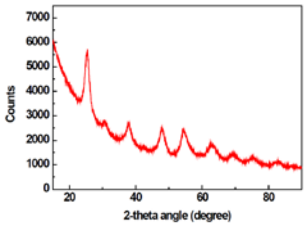
Crystallite size			HRTEM (KIT)	
Octanol-water partition coefficient			Extraction/ ICP-MS (ICN)	P_{OW}=0
Photocatalytic activity			Rhodamin-B Bleaching/UV-B-100<br>10mg/ml in Water (ICN)	high
Porosity		-/- or %	n.a.	n.a.
Pour density		cm3/g	(BJH) Pore Size Distribution and Volume by ASAP2020 (UU)	n.a.
Size distribution:<br>(suspension in water)	Modal value X_M (PDI)	nm	Zetasizer Nano ZS /UCD<br>DCS (UCD) (RWtAv / RNumAv)	61.63 (PDI: 0.157)<br>0.0236/0.0233
	Total concentration	mg/ml		1mg/ml
Size distribution:<br>(Aerosol)	Modal value X_M (σ_{geo})	nm	SMPS TSI, DMA 3071 with CPC 3022A / KIT<br>dispersed by electrospray	33.5 (σ_{geo}=1.32)
	Total concentration	mg/cm³ or #/cm³		1.2x 10^4/cm³
Solubility in _____		g/l		
Solubility in H2O after 30 days		mg/ml	Extraction/ ICP-MS (ICN)	none
Specific surface area		cm²/g	BET by ASAP2020 (UU)	n.a.
Surface chemistry				
Zeta potential (surface charge)		eV	Zetasizer Nano ZS /UU	-43.0 ± 0.4 mV<br>pH(10.27)

Summary of trace analyses		ICP-MS / KIT	
Ba	µg/g		14
Ca	µg/g		68
Cd	µg/g		0.2
K	µg/g		23
Mg	µg/g		2.8
Na	µg/g		101
Nb	µg/g		1.1
Pb	µg/g		0.5
Sn	µg/g		10
V	µg/g		5
Zn	µg/g		7.7

Electron microscopy
Method / technical data of microscope:
SOP: See KIT (LEM)

Magnification: 450.000

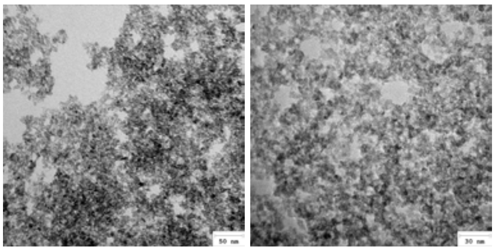

Electron Microscopy by KIT (LEM); Diameter : 10nm or smaller

UNIVLEEDS
TEM/ SEM

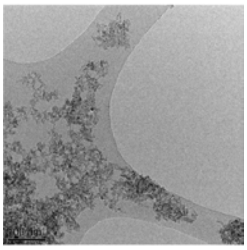

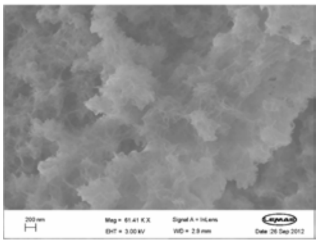

SEM-EDX

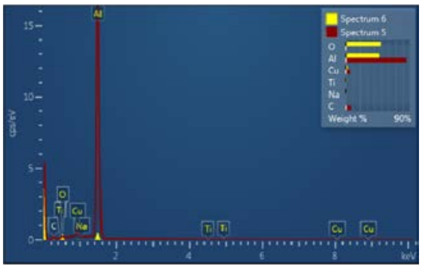

Radical formation potential by DCF-Test:
(H_2O_2 as positive control) (KIT)

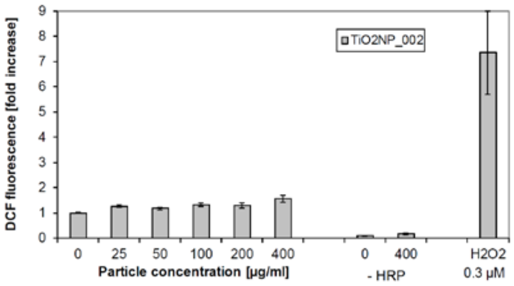

Result: TiO2 NP_002 induced a low increase of DCFH oxidation at high concentration of 400 µg/ml (1.6 fold of nanoparticle free control).

Comments and other relevant physical-chemical properties and material characterization information

**Place, Date Karlsruhe, 29.11.2016**	**Responsible H.-R. Paur, KIT**

REPRESENTATIVE TEST PARTICLES
Nanomaterial Identification
according to OECD
ENV/JM/MONO(2009)20/REV pp29.

Research Infrastructure QualityNano

Nanomaterial name	TiO_2-NP
Particle Code	TiO_2NP_003
Composition	TiO_2
Method of production	B2B Sol-Gel synthesis

Manufacturer /Institute/Date

Jordi Piella Bagaria; provided by ICN; 09/2012

Technology Expert: Jordi Piella Bagaria

Kind of suspension:

Suspension ☒ **Powder** ☐

Suspended in	miliQ-water
pH	5
stabilizer	5 mM TMAOH

**Property**	**unit**	**Method / Institute**	**Value**
Agglomeration/aggregation		HRTEM (KIT)	Agglomerated /crystalline
Crystalline phase		D5000 Diffractometer (UU)<br>PANalytical X´Pert diffractometer (ICN)	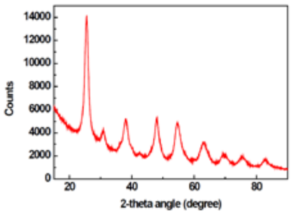
Crystallite size		HRTEM (KIT)	n.a.
Octanol-water partition coefficient		Extraction/ ICP-MS (ICN)	P_{OW}=0
Photocatalytic activity		Rhodamin-B Bleaching/UV-B-100<br>10mg/ml in Water (ICN)	high
Porosity	-/- or %	n.a.	n.a.
Pour density	cm3/g	(BJH) Pore Size Distribution and Volume by ASAP2020 (UU)	n.a.
Size distribution: (suspension in water) — Modal value X_M (PDI)	nm	Zetasizer Nano ZS /UCD<br>DCS (UCD) (RWtAv / RNumAv)	115.6 (PDI:0.174)<br>0.0390/0.0325
Size distribution: (suspension in water) — Total concentration	mg/ml		1mg/ml
Size distribution: (Aerosol) — Modal value X_M (σ_{geo})	nm	SMPS TSI, DMA 3071 with CPC 3022A / KIT<br>dispersed by electrospray	42.9 (σ_{geo}=1.5)
Size distribution: (Aerosol) — Total concentration	mg/cm³ or #/cm³		1.0x 10^4/cm³
Solubility in _____	g/l		
Solubility in H2O after 30 days	mg/ml	Extraction/ ICP-MS (ICN)	none
Specific surface area	cm²/g	BET by ASAP2020 (UU)	n.a.
Surface chemistry			
Zeta potential (surface charge)	eV	Zetasizer Nano ZS /UU	-39.6 ± 0.8 mV pH(9.63)

Summary of trace analyses		ICP-MS / KIT	
Ba	µg/g		12
Ca	µg/g		39
K	µg/g		24
Mg	µg/g		3.0
Na	µg/g		7700
Nb	µg/g		1.3
Ni	µg/g		1.8
Sn	µg/g		10
Zn	µg/g		9.8

Electron microscopy
Method / technical data of microscope:
SOP: See KIT (LEM)

Magnification: 450.000

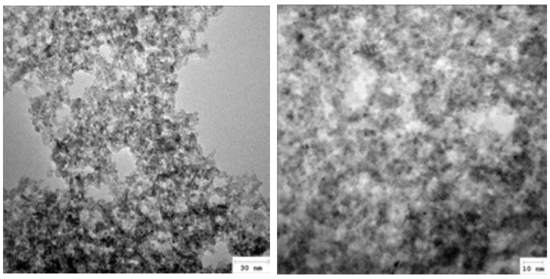

Electron Microscopy by KIT (LEM); Diameter : 10nm or smaller

UNIVLEEDS
TEM/ SEM

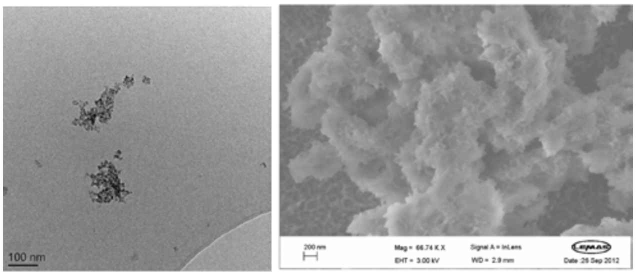

SEM-EDX

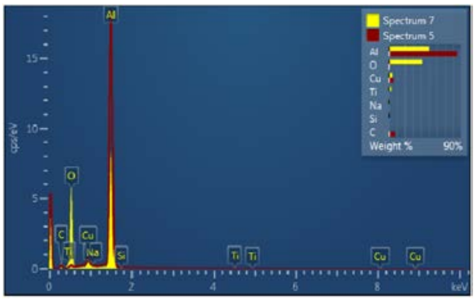

Radical formation potential by DCF-Test:
(H_2O_2 as positive control) (KIT)

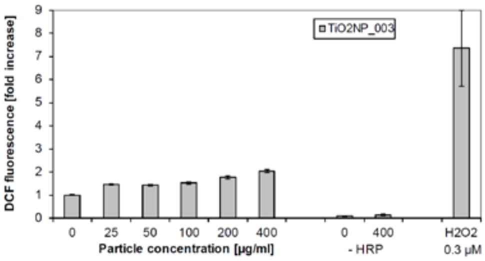

Result: TiO2 NP_003 induced a low increase of DCFH oxidation at high concentration of 400 µg/ml (2.0 fold of nanoparticle free control).

Comments and other relevant physical-chemical properties and material characterization information

**Place, Date Karlsruhe, 29.11.2016**	**Responsible H.-R. Paur, KIT**

**REPRESENTATIVE TEST PARTICLES**<br>Nanomaterial Identification<br>according to OECD<br>ENV/JM/MONO(2009)20/REV pp29.	Research Infrastructure QualityNano
Nanomaterial name: TiO_2-NP<br>Particle Code: TiO_2NP_004<br><br>Composition: TiO_2<br>Method of production: B2B Sol-Gel synthesis	**Manufacturer /Institute/Date**<br>**Jordi Piella Bagaria; provided by ICN; 09/2012**<br>**Technology Expert:** Jordi Piella Bagaria
**Kind of suspension:**	**Suspension** ☒ **Powder** ☐<br>Suspended in: miliQ-water<br>pH: 5<br>stabilizer: 5 mM TMAOH

**Property**		**unit**	**Method / Institute**	**Value**
Agglomeration/aggregation			HRTEM (KIT)	Agglomerated/ crystalline
Crystalline phase			D5000 Diffractometer (UU)<br>PANalytical X´Pert diffractometer (ICN)	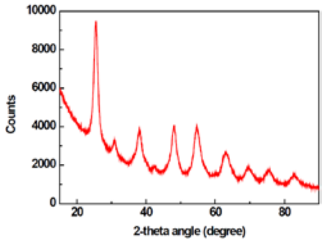
Crystallite size			HRTEM (KIT)	
Octanol-water partition coefficient			Extraction/ ICP-MS (ICN)	P_{OW}=0
Photocatalytic activity			Rhodamin-B Bleaching/UV-B-100<br>10mg/ml in Water (ICN)	high
Porosity		-/- or %	n.a.	n.a.
Pour density		cm3/g	(BJH) Pore Size Distribution and Volume by ASAP2020 (UU)	n.a.
Size distribution:<br>(suspension in water)	Modal value X_M (PDI)	nm	Zetasizer Nano ZS /UCD<br><br>DCS (UCD) (RWtAv / RNumAv)	105.1 (PDI:0.171)<br><br>0.0366/0.0315
	Total concentration	mg/ml		1mg/ml
Size distribution:<br>(Aerosol)	Modal value X_M (σ_{geo})	nm	SMPS TSI, DMA 3071 with CPC 3022A / KIT<br>dispersed by electrospray	50 (σ_{geo}=1.3)
	Total concentration	mg/cm³ or #/cm³		5.5x 10^3/cm^3
Solubility in _____		g/l		
Solubility in H_2O after 30 days		mg/ml	Extraction/ ICP-MS (ICN)	none
Specific surface area		cm²/g	BET by ASAP2020 (UU)	n.a.
Surface chemistry				
Zeta potential (surface charge)		eV	Zetasizer Nano ZS /UU	-40.0 ± 0.2 mV<br>pH(9.93)

Summary of trace analyses		ICP-MS / KIT	
Ba	µg/g		15
Ca	µg/g		30
Fe	µg/g		6
K	µg/g		25
Mg	µg/g		1.8
Na	µg/g		6100
Nb	µg/g		1.2
Ni	µg/g		3.2
Pb	µg/g		0.1
Sn	µg/g		9
Zn	µg/g		8.0

Electron microscopy
Method / technical data of microscope:
SOP: See KIT (LEM)

Magnification: 450.000

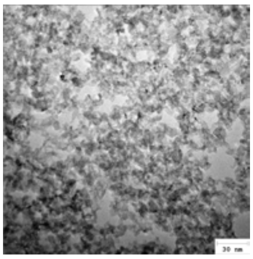
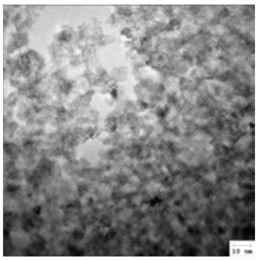

Electron Microscopy by KIT (LEM); Diameter : 10nm or smaller

UNIVLEEDS
TEM/ SEM

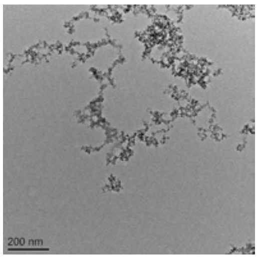

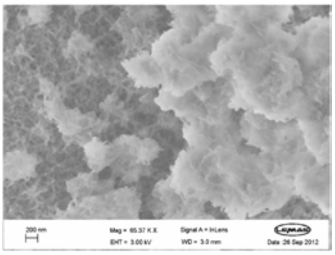

SEM-EDX

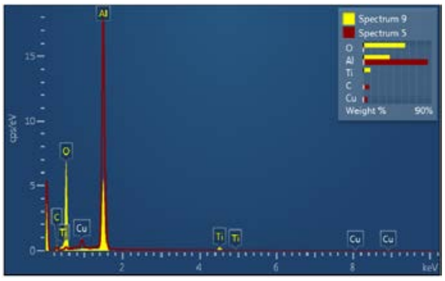

Radical formation potential by DCF-Test:
(H_2O_2 as positive control) (KIT)

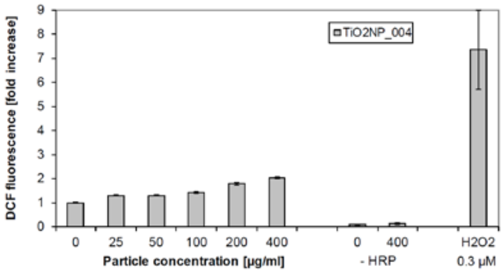

Result: TiO2 NP_004 induced a low increase of DCFH oxidation at high concentration of 400 µg/ml (2.0 fold of nanoparticle free control).

Comments and other relevant physical-chemical properties and material characterization information

Place, Date Karlsruhe, 29.11.2016

Responsible H.-R. Paur, KIT

REPRESENTATIVE TEST PARTICLES
Nanomaterial Identification
according to OECD
ENV/JM/MONO(2009)20/REV pp29.

Research Infrastructure QualityNano

Nanomaterial name	TiO_2-NP
Particle Code	TiO_2NP_005
Composition	TiO_2
Method of production	B2B Sol-Gel synthesis

Manufacturer /Institute/Date

Jordi Piella Bagaria; provided by ICN; 09/2012

Technology Expert: Jordi Piella Bagaria

Kind of suspension:

Suspension ☒ **Powder** ☐

Suspended in	miliQ-water
pH	5
stabilizer	5 mM TMAOH

**Property**		**unit**	**Method / Institute**	**Value**
Agglomeration/aggregation			HRTEM (KIT)	Agglomerated/ crystalline
Crystalline phase			D5000 Diffractometer (UU)<br>PANalytical X´Pert diffractometer (ICN)	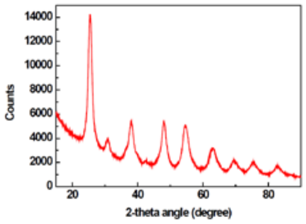
Crystallite size			HRTEM (KIT)	
Octanol-water partition coefficient			Extraction/ ICP-MS (ICN)	P_{OW}=0
Photocatalytic activity			Rhodamin-B Bleaching/UV-B-100<br>10mg/ml in Water (ICN)	high
Porosity		-/- or %	n.a.	n.a.
Pour density		cm3/g	(BJH) Pore Size Distribution and Volume by ASAP2020 (UU)	n.a.
Size distribution: (suspension in water)	Modal value X_M (PDI)	nm	Zetasizer Nano ZS /UCD<br>DCS (UCD) (RWtAv / RNumAv)	151.1 (PDI: 0.214)<br>0.0499/0.0370
	Total concentration	mg/ml		1mg/ml
Size distribution: (Aerosol)	Modal value X_M (σ_{geo})	nm	SMPS TSI, DMA 3071 with CPC 3022A / KIT<br>dispersed by electrospray	43.5 (σ_{geo}=1.39)
	Total concentration	mg/cm^3 or $\#/cm^3$		$2.0x\ 10^4/cm^3$
Solubility in _____		g/l		
Solubility in H2O after 30 days		mg/ml	Extraction/ ICP-MS (ICN)	none
Specific surface area		cm^2/g	BET by ASAP2020 (UU)	n.a.
Surface chemistry				
Zeta potential (surface charge)		eV	Zetasizer Nano ZS /UU	-36.9 ± 0.5 mV<br>pH(10.28)

Summary of trace analyses		ICP-MS / KIT	
Ba	µg/g		17
Ca	µg/g		39
Co	µg/g		0.5
Fe	µg/g		11
K	µg/g		26
Mg	µg/g		1.8
Na	µg/g		6700
Nb	µg/g		1.3
Ni	µg/g		1.9
Sn	µg/g		10
Zn	µg/g		8.8

Electron microscopy
Method / technical data of microscope:
SOP: See KIT (LEM)

Magnification: 450.000

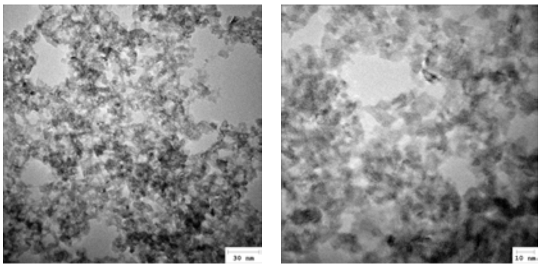

Electron Microscopy by KIT (LEM); Diameter : 10nm or smaller

UNIVLEEDS
TEM/ SEM

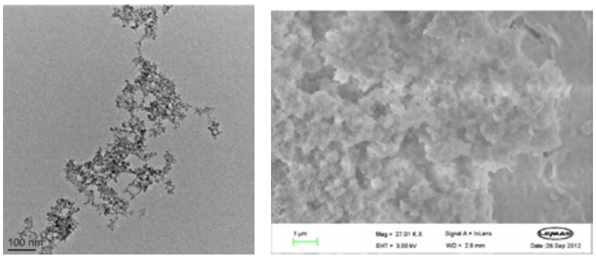

SEM-EDX

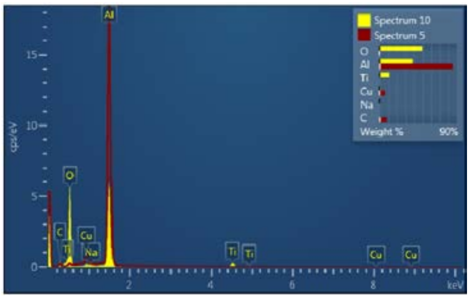

Radical formation potential by DCF-Test:
(H_2O_2 as positive control) (KIT)

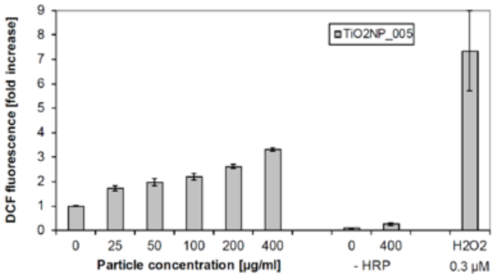

Result: TiO2 NP_005 induced a moderate increase of DCFH oxidation in dependence of concentration (3.3 fold of nanoparticle free control at 400 µg/ml).

Comments and other relevant physical-chemical properties and material characterization information

**Place, Date Karlsruhe, 29.11.2016**	**Responsible H.-R. Paur, KIT**

REPRESENTATIVE TEST PARTICLES
Nanomaterial Identification
according to OECD
ENV/JM/MONO(2009)20/REV pp29.

Nanomaterial name	TiO_2-NP
Particle Code	TiO_2NP_006
Composition	TiO_2
Method of production	B2B Sol-Gel synthesis

Manufacturer /Institute/Date

Jordi Piella Bagaria; provided by ICN; 09/2012

Technology Expert: Jordi Piella Bagaria

Kind of suspension:

Suspension ☒ **Powder** ☐

Suspended in	miliQ-water
pH	5
stabilizer	5 mM TMAOH

**Property**	**unit**	**Method / Institute**	**Value**
Agglomeration/aggregation		HRTEM (KIT)	Agglomerated/ crystalline
Crystalline phase		D5000 Diffractometer (UU)<br>PANalytical X´Pert diffractometer (ICN)	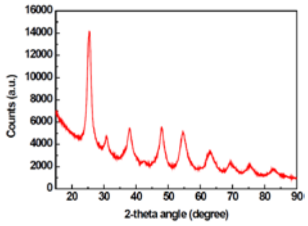
Crystallite size		HRTEM (KIT)	
Octanol-water partition coefficient		Extraction/ ICP-MS (ICN)	P_{OW}=0
Photocatalytic activity		Rhodamin-B Bleaching/UV-B-100<br>10mg/ml in Water (ICN)	high
Porosity	-/- or %	n.a.	n.a.
Pour density	cm3/g	(BJH) Pore Size Distribution and Volume by ASAP2020 (UU)	n.a.
Size distribution: (suspension in water) — Modal value X_M (PDI)	nm	Zetasizer Nano ZS /UCD<br>DCS (UCD) (RWtAv / RNumAv)	217.0 (PDI:0.388)<br>0.0542/0.0386
Size distribution: (suspension in water) — Total concentration	mg/ml		1mg/ml
Size distribution: (Aerosol) — Modal value X_M (σ_{geo})	nm	SMPS TSI, DMA 3071 with CPC 3022A / KIT<br>dispersed by electrospray	56.5 (σ_{geo}=1.47)
Size distribution: (Aerosol) — Total concentration	mg/cm³ or #/cm³		$1.2x\ 10^4/cm^3$
Solubility in _____	g/l		
Solubility in H2O after 30 days	mg/ml	Extraction/ ICP-MS (ICN)	none
Specific surface area	cm²/g	BET by ASAP2020 (UU)	n.a.
Surface chemistry			
Zeta potential (surface charge)	eV	Zetasizer Nano ZS /UU	-33.6 ± 1.2 mV<br>pH(10.10)

Summary of trace analyses		ICP-MS / KIT	
Ba	µg/g		21
Ca	µg/g		80
Fe	µg/g		11
K	µg/g		52
Mg	µg/g		4
Na	µg/g		140
Nb	µg/g		1.1
Pb	µg/g		1.2
Sn	µg/g		11
Zn	µg/g		9.6

Electron microscopy

Method / technical data of microscope:

SOP: See KIT (LEM)

Magnification: 450.000

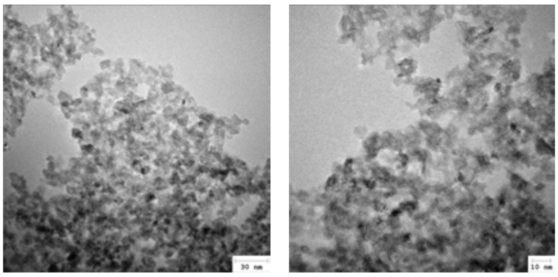

Electron Microscopy by KIT (LEM); Diameter : 10nm or smaller

UNIVLEEDS

TEM/ SEM

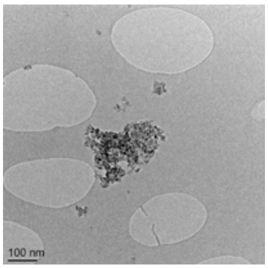

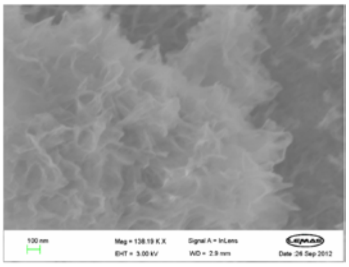

SEM-EDX

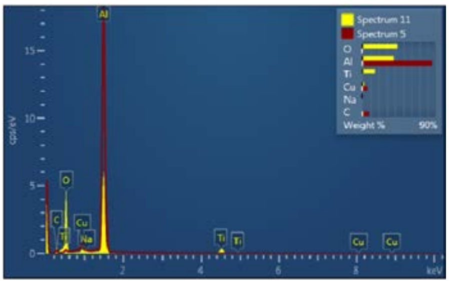

Radical formation potential by DCF-Test:

(H_2O_2 as positive control) (KIT)

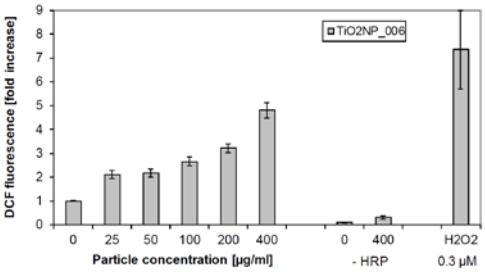

Result: TiO2 NP_006 induced a moderate increase of DCFH oxidation in dependence of concentration (4.8 fold of nanoparticle free control at 400 µg/ml).

Comments and other relevant physical-chemical properties and material characterization information

**Place, Date Karlsruhe, 29.11.2016**	**Responsible H.-R. Paur, KIT**

**REPRESENTATIVE TEST PARTICLES**<br>Nanomaterial Identification<br>according to OECD<br>ENV/JM/MONO(2009)20/REV pp29.	Research Infrastructure QualityNano
Nanomaterial name: TiO_2-NP<br>Particle Code: TiO_2NP_007<br>Composition: TiO_2<br>Method of production: B2B Sol-Gel synthesis	**Manufacturer /Institute/Date**<br>**Jordi Piella Bagaria; provided by ICN; 09/2012**<br>**Technology Expert:** Jordi Piella Bagaria
**Kind of suspension:**	**Suspension** ☒ **Powder** ☐<br>Suspended in: miliQ-water<br>pH: 5<br>stabilizer: 5 mM TMAOH

**Property**		**unit**	**Method / Institute**	**Value**
Agglomeration/aggregation			HRTEM (KIT)	Agglomerated/ crystalline
Crystalline phase			D5000 Diffractometer (UU)<br>PANalytical X´Pert diffractometer (ICN)	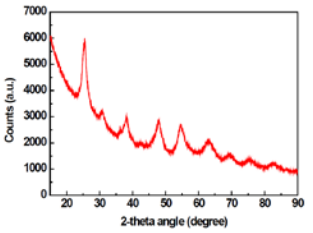
Crystallite size			HRTEM (KIT)	
Octanol-water partition coefficient			Extraction/ ICP-MS (ICN)	P_{OW}=0
Photocatalytic activity			Rhodamin-B Bleaching/UV-B-100<br>10mg/ml in Water (ICN)	medium
Porosity		-/- or %	n.a.	n.a.
Pour density		cm3/g	(BJH) Pore Size Distribution and Volume by ASAP2020 (UU)	n.a.
Size distribution:<br>(suspension in water)	Modal value X_M (PDI)	nm	Zetasizer Nano ZS /UCD<br>DCS (UCD) (RWtAv / RNumAv)	281.3 (PDI:0.409)<br>0.0700/0.0406
	Total concentration	mg/ml		1mg/ml
Size distribution:<br>(Aerosol)	Modal value X_M (σ_{geo})	nm	SMPS TSI, DMA 3071 with CPC 3022A / KIT<br>dispersed by electrospray	59 (σ_{geo}=1.45)
	Total concentration	mg/cm³ or #/cm³		6.0x 10^3/cm^3
Solubility in _____		g/l		
Solubility in H_2O after 30 days		mg/ml	Extraction/ ICP-MS (ICN)	none
Specific surface area		cm²/g	BET by ASAP2020 (UU)	n.a.
Surface chemistry				
Zeta potential (surface charge)		eV	Zetasizer Nano ZS /UU	-37.4 ± 1.4 mV<br>pH(9.13)

Summary of trace analyses		ICP-MS / KIT	
Ba	µg/g		50
Ca	µg/g		130
Fe	µg/g		60
Na	µg/g		22000
Sn	µg/g		9

Electron microscopy
Method / technical data of microscope:
SOP: See KIT (LEM)

Magnification: 450.000

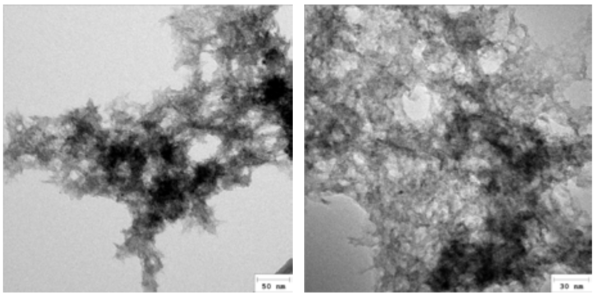

Electron Microscopy by KIT (LEM); Diameter : ca. 5 nm

UNIVLEEDS
TEM/ SEM

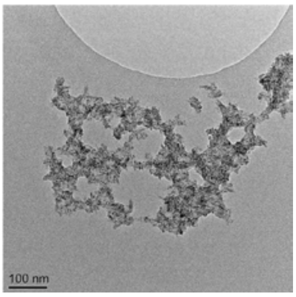

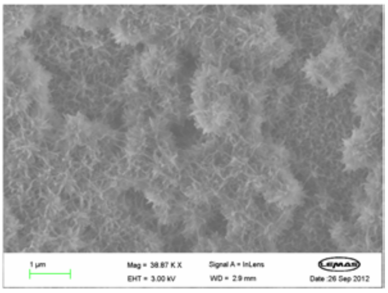

SEM-EDX

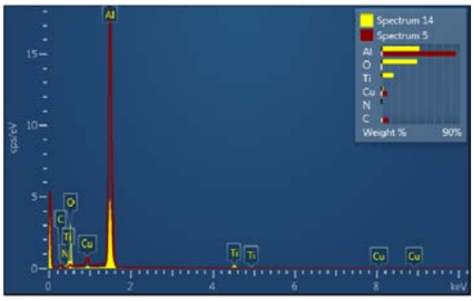

Radical formation potential by DCF-Test:
(H_2O_2 as positive control) (KIT)

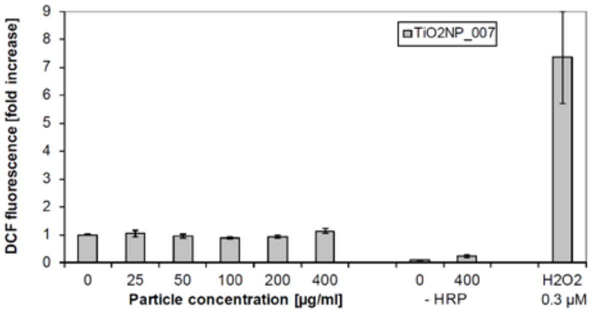

Result: TiO2 NP_007 induced a no increase of DCFH oxidation.

Comments and other relevant physical-chemical properties and material characterization information

**Place, Date Karlsruhe, 29.11.2016**	**Responsible H.-R. Paur, KIT**

REPRESENTATIVE TEST PARTICLES
Nanomaterial Identification
according to OECD
ENV/JM/MONO(2009)20/REV pp29.

Nanomaterial name	TiO_2-NP
Particle Code	TiO_2NP_008
Composition	TiO_2
Method of production	B2B Sol-Gel synthesis

Manufacturer /Institute/Date

Jordi Piella Bagaria; provided by ICN; 09/2012

Technology Expert: Jordi Piella Bagaria

Kind of suspension:

Suspension ☒ **Powder** ☐

Suspended in	miliQ-water
pH	7
stabilizer	5 mM TMAOH

**Property**		**unit**	**Method / Institute**	**Value**
Agglomeration/aggregation			HRTEM (KIT)	Agglomerated/ crystalline
Crystalline phase			D5000 Diffractometer (UU)<br>PANalytical X´Pert diffractometer (ICN)	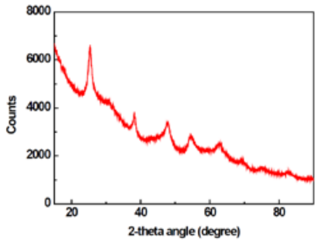
Crystallite size			HRTEM (KIT)	
Octanol-water partition coefficient			Extraction/ ICP-MS (ICN)	P_{OW}=0
Photocatalytic activity			Rhodamin-B Bleaching/UV-B-100 10mg/ml in Water (ICN)	None-medium
Porosity		-/- or %	n.a.	n.a.
Pour density		cm3/g	(BJH) Pore Size Distribution and Volume by ASAP2020 (UU)	n.a.
Size distribution: (suspension in water)	Modal value X_M (PDI)	nm	Zetasizer Nano ZS /UCD<br>DCS (UCD) (RWtAv / RNumAv)	265.3 (PDI:0.287)<br>0.0618/0.0407
	Total concentration	mg/ml		1mg/ml
Size distribution: (Aerosol)	Modal value X_M (σ_{geo})	nm	SMPS TSI, DMA 3071 with CPC 3022A / KIT<br>dispersed by electrospray	70 (σ_{geo}=1.5)
	Total concentration	mg/cm³ or #/cm³		3.7x 10^3/cm^3
Solubility in _____		g/l		
Solubility in H2O after 30 days		mg/ml	Extraction/ ICP-MS (ICN)	none
Specific surface area		cm²/g	BET by ASAP2020 (UU)	n.a.
Surface chemistry				
Zeta potential (surface charge)		eV	Zetasizer Nano ZS /UU	-36.9 ± 0.3 mV<br>pH 10.23

Summary of trace analyses		ICP-MS / KIT	
Ba	µg/g		28
Ca	µg/g		128
Fe	µg/g		25
K	µg/g		129
Na	µg/g		96000
Nb	µg/g		1.3
Ni	µg/g		2.2
Pb	µg/g		0.2
Sn	µg/g		8

Electron microscopy

Method / technical data of microscope:

SOP: See KIT (LEM)

Magnification: 450.000

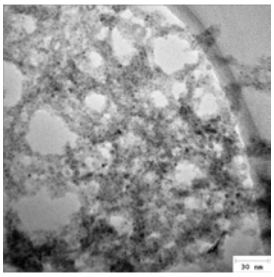

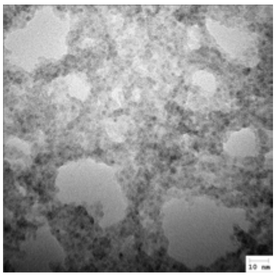

Electron Microscopy by KIT (LEM); Diameter : 5nm and smaller

UNIVLEEDS

TEM/ SEM

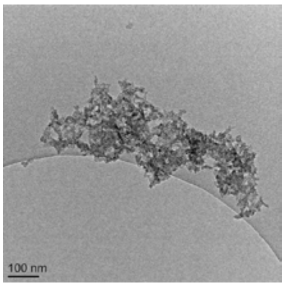

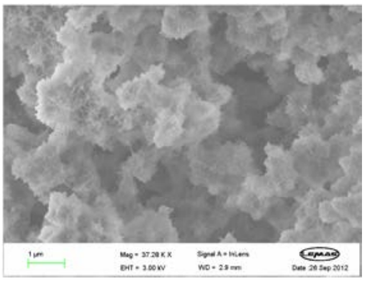

SEM-EDX

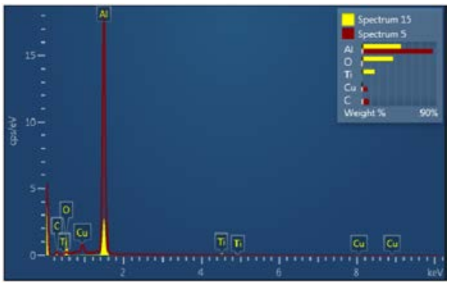

Radical formation potential by DCF-Test:

(H_2O_2 as positive control) (KIT)

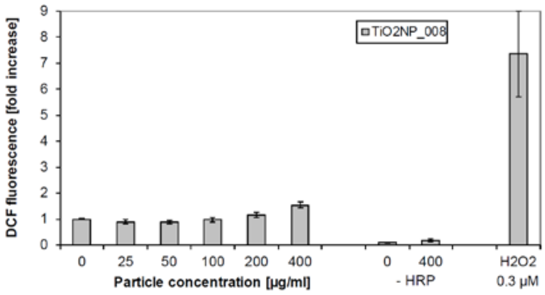

Result: TiO2 NP_008 induced a low increase of DCFH oxidation at high concentration of 400 µg/ml (1.5 fold of nanoparticle free control).

Comments and other relevant physical-chemical properties and material characterization information

**Place, Date Karlsruhe, 29.11.2016**	**Responsible H.-R. Paur, KIT**

Supplemental S6: Physicochemical characterization data sheets of PS-NH_2 NMs

**REPRESENTATIVE TEST PARTICLES**<br>Nanomaterial Identification<br>according to OECD<br>ENV/JM/MONO(2009)20/REV pp29.	Research Infrastructure QualityNano
Nanomaterial name: Latex<br>Particle Code: PSNP_001<br><br>Composition: PS<br>Method of production: Sol-Gel synthesis	**Manufacturer /Institute/Date**<br>**Eugene Mahon ; provided by UCD; 10/2012**<br>**Technology Expert:** Eugene Mahon
**Kind of suspension:**	**Suspension** / **Powder**<br>Suspended in: Pure water<br>pH:<br>stabilizer: none

**Property**		**unit**	**Method / Institute**	**Value**
Agglomeration/aggregation			HRTEM (KIT)	Agglomerated
Crystalline phase			D5000 Diffractometer (UU)<br>PANalytical X´Pert diffractometer (ICN)	Amorphous
Crystallite size			HRTEM (KIT)	n.a.
Octanol-water partition coefficient			Determination of absorbance (ICN)	P_{OW}=0
Photocatalytic activity			Rhodamin-B Bleaching/UV-B-100<br>0.05mg/ml in Water (ICN)	none
Porosity		-/- or %	n.a.	none
Pour density		cm3/g	(BJH) Pore Size Distribution and Volume by ASAP2020 (UU)	none
Size distribution: (suspension in water)	Modal value X_M (PDI)	nm	Zetasizer Nano ZS /UCD<br>DCS (UCD) (RWtAv / RNumAv)	115.9 (PDI:0.029) NM: 94.29<br>n.a.
	Total concentration	mg/ml		10
Size distribution: (Aerosol)	Modal value X_M (σ_{geo})	nm	SMPS TSI, DMA 3071 with CPC 3022A / KIT<br>dispersed by electrospray	120 (σ_{geo}=1.15)
	Total concentration	mg/cm³ or #/cm³		3.4x 10^3/cm^3
Solubility in _____		g/l		
Solubility in H_2O		g/l	Extraction/ ICP-MS (ICN)	n.a.
Specific surface area		m²/g	BET by ASAP2020 (UU)	none
Surface chemistry				
Zeta potential (surface charge)		mV	Zetasizer Nano ZS /UU	+48.7 ± 0.5<br>pH: 6.1

Summary of trace analyses		ICP-MS / KIT	
Na	µg/g		42
Ba	µg/g		2.1
Yb	µg/g		0.01
Ta	µg/g		0.05
W	µg/g		0.05

Electron microscopy
Method / technical data of microscope:
SOP: See KIT (LEM)

Magnification: 450.000

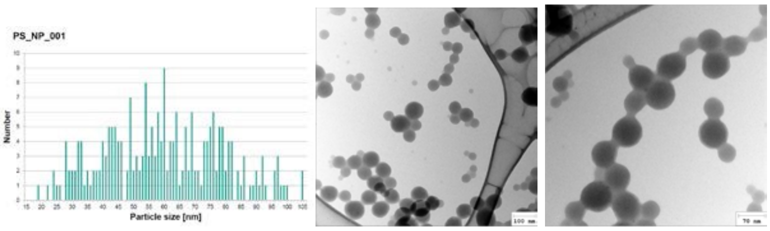

Electron Microscopy by KIT (LEM); Diameter : 10-100 nm

UNIVLEEDS
SEM

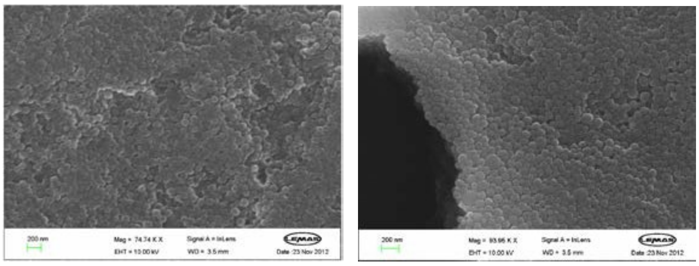

SEM-EDX

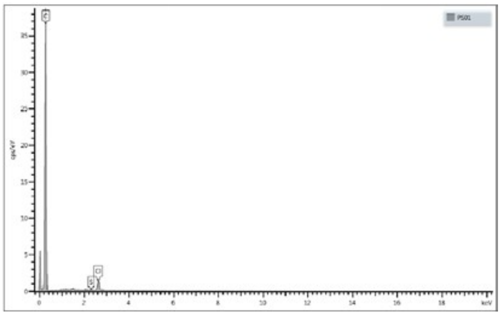

Radical formation potential by DCF-Test:

(H_2O_2 as positive control) (KIT)

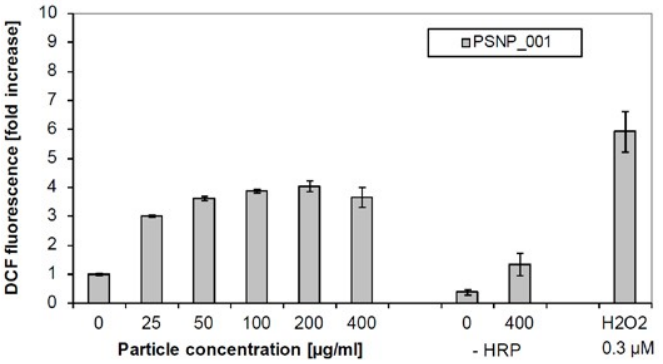

Result: PSNP_001 induced a moderate increase of DCFH oxidation (4.0 fold of nanoparticle free control at 200 µg/ml). At higher concentrations, the DCF fluorescence decreased probably due to adsorption of the dye on the particle surface.

Comments and other relevant physical-chemical properties and material characterization information

**Place, Date Karlsruhe, 29.11.2016**	**Responsible H.-R. Paur, KIT**

**REPRESENTATIVE TEST PARTICLES**<br>Nanomaterial Identification<br>according to OECD<br>ENV/JM/MONO(2009)20/REV pp29.	Research Infrastructure QualityNano
Nanomaterial name: Latex<br>Particle Code: PSNP_002	**Manufacturer /Institute/Date**<br>**Eugene Mahon ; provided by UCD; 10/2012**<br>**Technology Expert:** Eugene Mahon
Composition: PS<br>Method of production: Sol-Gel synthesis	

**Kind of suspension:**	**Suspension**	**Powder**
	Suspended in	Pure water
	pH	
	stabilizer	none

**Property**		**unit**	**Method / Institute**	**Value**
Agglomeration/aggregation			HRTEM (KIT)	Agglomerated
Crystalline phase			D5000 Diffractometer (UU)<br>PANalytical X´Pert diffractometer (ICN)	Amorphous
Crystallite size			HRTEM (KIT)	n.a.
Octanol-water partition coefficient			Determination of absorbance (ICN)	P_{OW}=0
Photocatalytic activity			Rhodamin-B Bleaching/UV-B-100<br>0.05mg/ml in Water (ICN)	none
Porosity		-/- or %	n.a.	none
Pour density		cm3/g	(BJH) Pore Size Distribution and Volume by ASAP2020 (UU)	none
Size distribution: (suspension in water)	Modal value X_M (PDI)	nm	Zetasizer Nano ZS /UCD<br>UNIVLEEDS<br>DCS (UCD) (RWtAv / RNumAv)	119.1 (PDI:0.80) NM: 91.2<br>120.5 (PDI: 0.113)
	Total concentration	mg/ml		10
Size distribution: (Aerosol)	Modal value X_M (σ_{geo})	nm	SMPS TSI. DMA 3071 with CPC 3022A / KIT<br>dispersed by electrospray	106 (σ_{geo}=1.12)
	Total concentration	mg/cm³ or #/cm³		4.4x 10^3/cm^3
Solubility in _____		g/l		
Solubility in H_2O		g/l	Extraction/ ICP-MS (ICN)	
Specific surface area		m²/g	BET by ASAP2020 (UU)	none
Surface chemistry				
Zeta potential (surface charge)		mV	Zetasizer Nano ZS /UU	+44.4 ± 0.3<br>pH: 6.0

<table>
<tr><td>Summary of trace analyses</td><td></td><td>ICP-MS / KIT</td><td></td></tr>
<tr><td>Na</td><td>µg/g</td><td></td><td>103</td></tr>
<tr><td>Mg</td><td>µg/g</td><td></td><td>23</td></tr>
<tr><td>Fe</td><td>µg/g</td><td></td><td>11</td></tr>
<tr><td>W</td><td>µg/g</td><td></td><td>0.07</td></tr>
<tr><td>Electron microscopy
Method / technical data of microscope:
SOP: See KIT (LEM)

Magnification: 450.000

UNIVLEEDS
SEM-scale bar/ SEM

SEM-EDX</td><td colspan="3">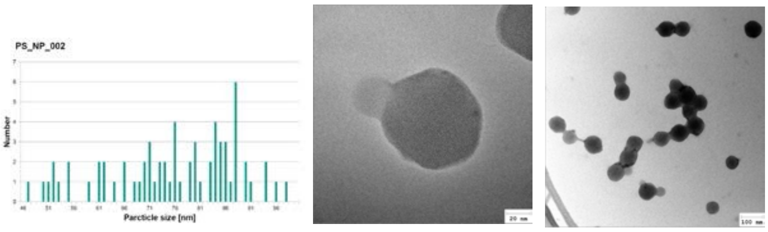

Electron Microscopy by KIT (LEM); Diameter: 40-90 nm
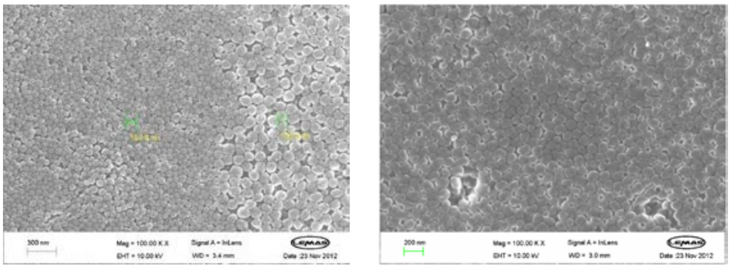
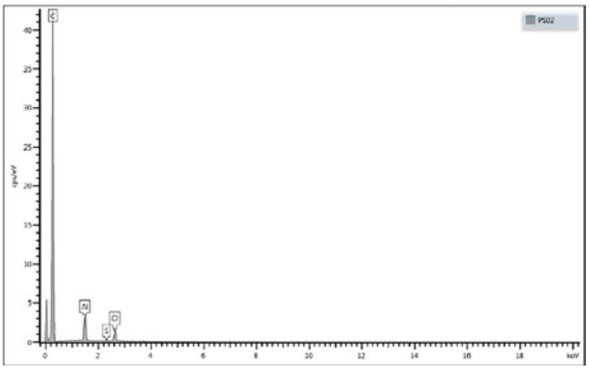
</td></tr>
<tr><td>Radical formation potential by DCF-Test:
(H_2O_2 as positive control) (KIT)</td><td colspan="3">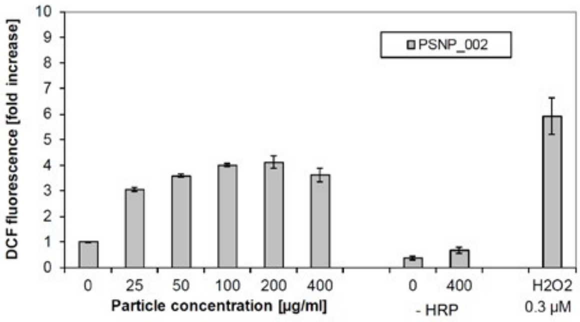

Result: PSNP_002 induced a moderate increase of DCFH oxidation (4.1 fold of nanoparticle free control at 200 µg/ml). At higher concentrations, the DCF fluorescence decreased probably due to adsorption of the dye on the particle surface.</td></tr>
<tr><td colspan="4">Comments and other relevant physical-chemical properties and material characterization information</td></tr>
<tr><td colspan="2">Place, Date Karlsruhe, 09.04.2013</td><td colspan="2">Responsible H.-R. Paur, KIT</td></tr>
</table>

**REPRESENTATIVE TEST PARTICLES**<br>Nanomaterial Identification<br>according to OECD<br>ENV/JM/MONO(2009)20/REV pp29.	Research Infrastructure QualityNano
Nanomaterial name: Latex<br>Particle Code: PSNP_003<br>Composition: PS<br>Method of production: Sol-Gel synthesis	**Manufacturer /Institute/Date**<br>**Eugene Mahon ; provided by UCD; 10/2012**<br>**Technology Expert:** Eugene Mahon
**Kind of suspension:**	**Suspension** / **Powder**<br>Suspended in: Pure water<br>pH:<br>stabilizer: none

**Property**		**unit**	**Method / Institute**	**Value**
Agglomeration/aggregation			HRTEM (KIT)	Agglomerated
Crystalline phase			D5000 Diffractometer (UU)<br>PANalytical X´Pert diffractometer (ICN)	Amorphous
Crystallite size			HRTEM (KIT)	n.a.
Octanol-water partition coefficient			Determination of absorbance (ICN)	P_{OW}=0
Photocatalytic activity			Rhodamin-B Bleaching/UV-B-100<br>0.05mg/ml in Water (ICN)	none
Porosity		-/- or %	n.a.	none
Pour density		cm3/g	(BJH) Pore Size Distribution and Volume by ASAP2020 (UU)	none
Size distribution:<br>(suspension in water)	Modal value X_M (PDI)	nm	Zetasizer Nano ZS /UCD<br>UNIVLEEDS<br>DCS (UCD) (RWtAv / RNumAv)	140.8 (PDI:0.011)<br>NM: 121.0<br>123.4 (PDI: 0.101)
	Total concentration	mg/ml		10
Size distribution:<br>(Aerosol)	Modal value X_M (σ_{geo})	nm	SMPS TSI. DMA 3071 with CPC 3022A / KIT<br>dispersed by electrospray	88.2 (σ_{geo}=1.18)
	Total concentration	mg/cm³ or #/cm³		3.1x 10^4/cm^3
Solubility in _____		g/l		
Solubility in H_2O		g/l	Extraction/ ICP-MS (ICN)	
Specific surface area		m²/g	BET by ASAP2020 (UU)	none
Surface chemistry				
Zeta potential (surface charge)		mV	Zetasizer Nano ZS /UU	+50.9 ± 1.9<br>pH:6.1

Summary of trace analyses		ICP-MS / KIT	
Na	µg/g		113
Cr	µg/g		1.1
Fe	µg/g		17
Ta	µg/g		0.01
W	µg/g		0.08

Electron microscopy
Method / technical data of microscope:
SOP: See KIT (LEM)

Magnification: 450.000

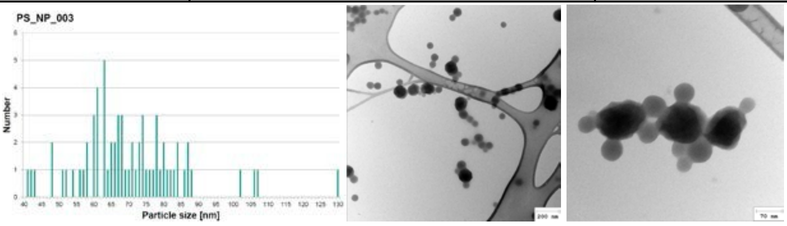

Electron Microscopy by KIT (LEM); Diameter : 25-120 nm

UNIVLEEDS
SEM-scale bar/ SEM

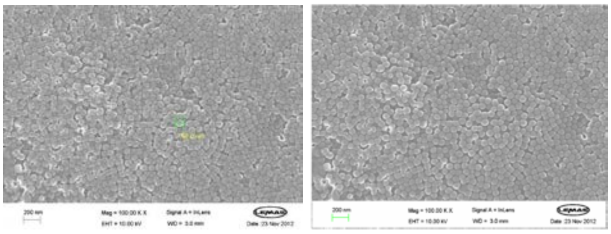

SEM-EDX

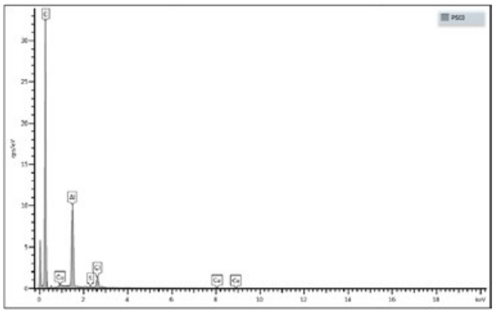

Radical formation potential by DCF-Test:
(H_2O_2 as positive control) (KIT)

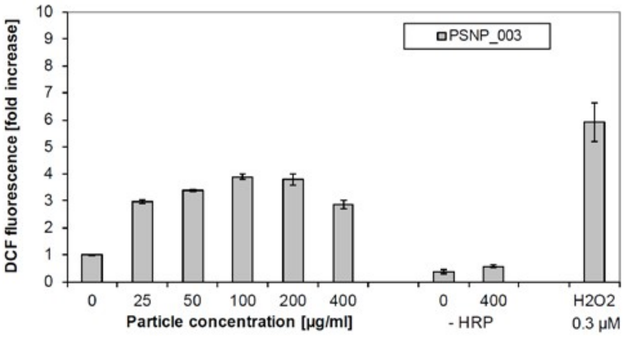

Result: PSNP_003 induced a moderate increase of DCFH oxidation (3.8 fold of nanoparticle free control at 200 µg/ml). At higher concentrations, the DCF fluorescence decreased probably due to adsorption of the dye on the particle surface.

Comments and other relevant physical-chemical properties and material characterization information

**Place. Date Karlsruhe. 09.04.2013**	**Responsible H.-R. Paur. KIT**

REPRESENTATIVE TEST PARTICLES

Nanomaterial Identification
according to OECD
ENV/JM/MONO(2009)20/REV pp29.

Research Infrastructure QualityNano

Nanomaterial name	Latex
Particle Code	PSNP_004
Composition	PS
Method of production	Sol-Gel synthesis

Manufacturer /Institute/Date

Eugene Mahon ; provided by UCD; 10/2012

Technology Expert: Eugene Mahon

Kind of suspension:

**Suspension**	**Powder**
Suspended in	Pure water
pH	
stabilizer	none

**Property**		**unit**	**Method / Institute**	**Value**
Agglomeration/aggregation			HRTEM (KIT)	Agglomerated
Crystalline phase			D5000 Diffractometer (UU)<br>PANalytical X´Pert diffractometer (ICN)	Amorphous
Crystallite size			HRTEM (KIT)	n.a.
Octanol-water partition coefficient			Determination of absorbance (ICN)	P_{OW}=0
Photocatalytic activity			Rhodamin-B Bleaching/UV-B-100<br>0.05mg/ml in Water (ICN)	none
Porosity		-/- or %	n.a.	none
Pour density		cm3/g	(BJH) Pore Size Distribution and Volume by ASAP2020 (UU)	none
Size distribution: (suspension in water)	Modal value X_M (PDI)	nm	Zetasizer Nano ZS /UCD<br>UNIVLEEDS<br>DCS (UCD) (RWtAv / RNumAv)	130.9 (PDI:0.033) NM: 108.4<br>123.8 (PDI:0.049)
	Total concentration	mg/ml		10
Size distribution: (Aerosol)	Modal value X_M (σ_{geo})	nm	SMPS TSI. DMA 3071 with CPC 3022A / KIT<br>dispersed by electrospray	135 (σ_{geo}=1.18)
	Total concentration	mg/cm³ or #/cm³		1.5x 10^4/cm^3
Solubility in _____		g/l		
Solubility in H_2O		g/l	Extraction/ ICP-MS (ICN)	
Specific surface area		m²/g	BET by ASAP2020 (UU)	none
Surface chemistry				
Zeta potential (surface charge)		mV	Zetasizer Nano ZS /UU	+49.7 ± 0.6<br>pH:6.1

Summary of trace analyses		ICP-MS / KIT	
Na	µg/g		93

Electron microscopy

Method / technical data of microscope:

SOP: See KIT (LEM)

Magnification: 450.000

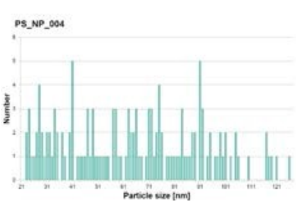

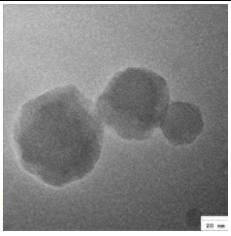

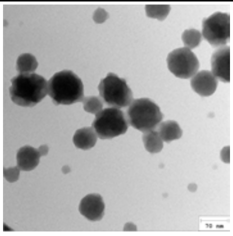

Electron Microscopy by KIT (LEM); Diameter : 15-100 nm

UNIVLEEDS

SEM-scale bar/ SEM

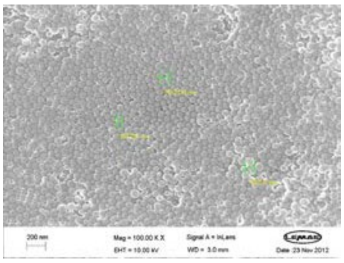

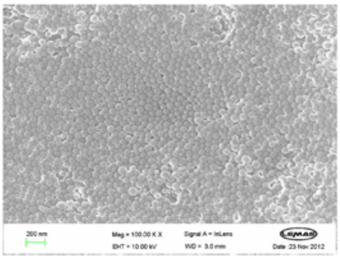

SEM-EDX

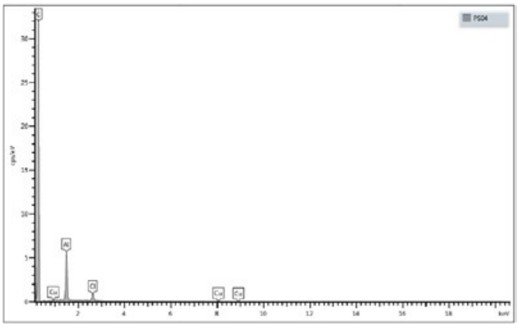

Radical formation potential by DCF-Test:

(H_2O_2 as positive control) (KIT)

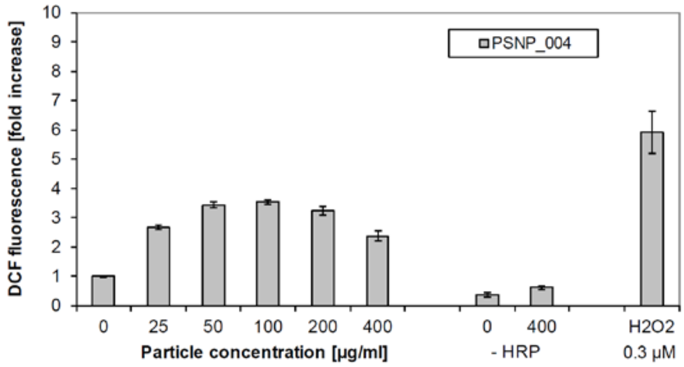

Result: PSNP_004 induced a moderate increase of DCFH oxidation (3.2 fold of nanoparticle free control at 200 µg/ml). At high concentrations, the DCF fluorescence decreased probably due to adsorption of the dye on the particle surface.

Comments and other relevant physical-chemical properties and material characterization information

**Place, Date Karlsruhe, 09.04.2013**	**Responsible H.-R. Paur. KIT**

REPRESENTATIVE TEST PARTICLES<br>Nanomaterial Identification<br>according to OECD<br>ENV/JM/MONO(2009)20/REV pp29.	Research Infrastructure QualityNano
Nanomaterial name: Latex<br>Particle Code: PSNP_005<br><br>Composition: PS<br>Method of production: Sol-Gel synthesis	**Manufacturer /Institute/Date**<br>**Eugene Mahon ; provided by UCD; 10/2012**<br>**Technology Expert:** Eugene Mahon
**Kind of suspension:**	**Suspension** / **Powder**<br>Suspended in: Pure water<br>pH:<br>stabilizer: none

**Property**		**unit**	**Method / Institute**	**Value**
Agglomeration/aggregation			HRTEM (KIT)	Agglomerated
Crystalline phase			D5000 Diffractometer (UU)<br>PANalytical X´Pert diffractometer (ICN)	Amorphous
Crystallite size			HRTEM (KIT)	n.a.
Octanol-water partition coefficient			Determination of absorbance (ICN)	P_{OW}=0
Photocatalytic activity			Rhodamin-B Bleaching/UV-B-100<br>0.05mg/ml in Water (ICN)	none
Porosity		-/- or %	n.a.	none
Pour density		cm3/g	(BJH) Pore Size Distribution and Volume by ASAP2020 (UU)	none
Size distribution:<br>(suspension in water)	Modal value X_M (PDI)	nm	Zetasizer Nano ZS /UCD<br>UNIVLEEDS<br>DCS (UCD) (RWtAv / RNumAv)	105.7 (PDI:0.015)<br>NM: 86.9<br>103.3 (PDI:0.068)
	Total concentration	mg/ml		10
Size distribution:<br>(Aerosol)	Modal value X_M (σ_{geo})	nm	SMPS TSI. DMA 3071 with CPC 3022A / KIT<br>dispersed by electrospray	94 (σ_{geo}=1.15)
	Total concentration	mg/cm³ or #/cm³		2.3x 10^4/cm^3
Solubility in _____		g/l		
Solubility in H_2O		g/l	Extraction/ ICP-MS (ICN)	
Specific surface area		m²/g	BET by ASAP2020 (UU)	none
Surface chemistry				
Zeta potential (surface charge)		mV	Zetasizer Nano ZS /UU	+52.1 ± 0.2<br>pH:6.1

Summary of trace analyses		ICP-MS / KIT	
Na	µg/g		91
Ir	µg/g		0.13

Electron microscopy

Method / technical data of microscope:

SOP: See KIT (LEM)

magnification: 450.000

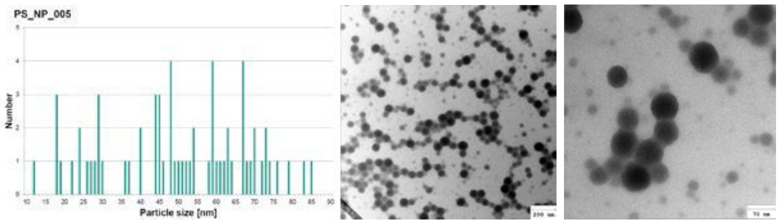

Electron Microscopy by KIT (LEM); Diameter : 10-90 nm

UNIVLEEDS

SEM-scale bar/ SEM

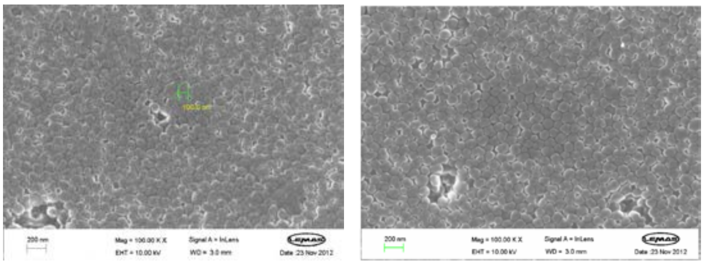

SEM-EDX

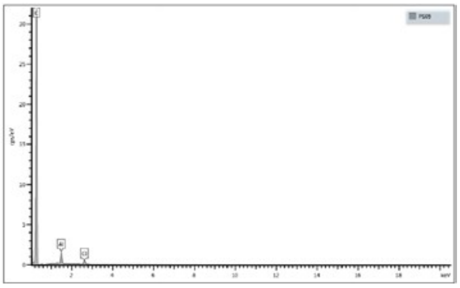

Radical formation potential by DCF-Test:

(H_2O_2 as positive control) (KIT)

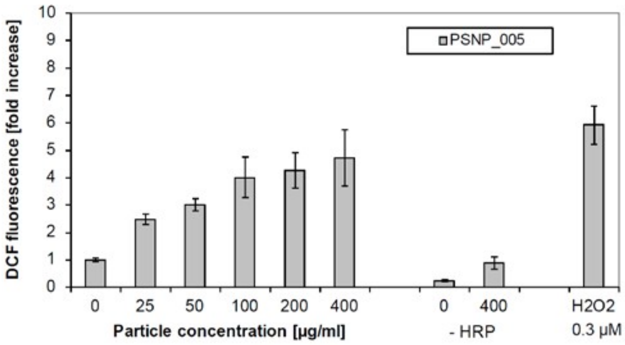

Result: PSNP_005 induced a moderate increase of DCFH oxidation (4.2 fold of nanoparticle free control at 200 µg/ml).

Comments and other relevant physical-chemical properties and material characterization information

**Place, Date Karlsruhe, 09.04.2013**	**Responsible H.-R. Paur, KIT**

**REPRESENTATIVE TEST PARTICLES**<br>Nanomaterial Identification according to OECD ENV/JM/MONO(2009)20/REV pp29.	Research Infrastructure QualityNano
Nanomaterial name: Latex<br>Particle Code: PSNP_006<br>Composition: PS<br>Method of production: Sol-Gel synthesis	**Manufacturer /Institute/Date**<br>**Eugene Mahon ; provided by UCD; 10/2012**<br>**Technology Expert:** Eugene Mahon
**Kind of suspension:**	**Suspension** — Suspended in: Pure water; pH: ; stabilizer: none<br>**Powder**

**Property**		**unit**	**Method / Institute**	**Value**
Agglomeration/aggregation			HRTEM (KIT)	Agglomerated
Crystalline phase			D5000 Diffractometer (UU)<br>PANalytical X´Pert diffractometer (ICN)	Amorphous
Crystallite size			HRTEM (KIT)	n.a.
Octanol-water partition coefficient			Determination of absorbance (ICN)	P_{OW}=0
Photocatalytic activity			Rhodamin-B Bleaching/UV-B-100<br>0.05mg/ml in Water (ICN)	none
Porosity		-/- or %	n.a.	none
Pour density		cm3/g	(BJH) Pore Size Distribution and Volume by ASAP2020 (UU)	none
Size distribution: (suspension in water)	Modal value X_M (PDI)	nm	Zetasizer Nano ZS /UCD<br>UNIVLEEDS<br>DCS (UCD) (RWtAv / RNumAv)	153.7 (PDI:0.041) NM: 132.8<br>144.3 (PDI:0.020)
	Total concentration	mg/ml		10
Size distribution: (Aerosol)	Modal value X_M (σ_{geo})	nm	SMPS TSI. DMA 3071 with CPC 3022A / KIT<br>dispersed by electrospray	168.5 (σ_{geo}=1.09)
	Total concentration	mg/cm³ or #/cm³		1.5x 10^4/cm^3
Solubility in _____		g/l		
Solubility in H_2O		g/l	Extraction/ ICP-MS (ICN)	
Specific surface area		m²/g	BET by ASAP2020 (UU)	none
Surface chemistry				
Zeta potential (surface charge)		mV	Zetasizer Nano ZS /UU	+50.7 ± 1.0<br>pH:6.1

Summary of trace analyses			
Na	µg/g		74
Ir	µg/g		0.12

Electron microscopy

Method / technical data of microscope:

SOP: See KIT (LEM)

Magnification: 450.000

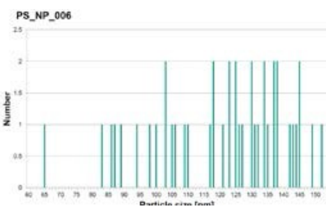

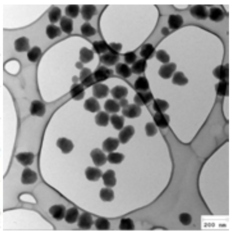

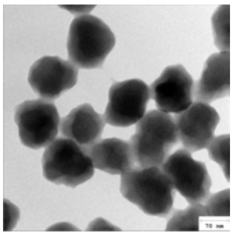

Electron Microscopy by KIT (LEM); Diameter : 30-50 nm

UNIVLEEDS

TEM/ SEM

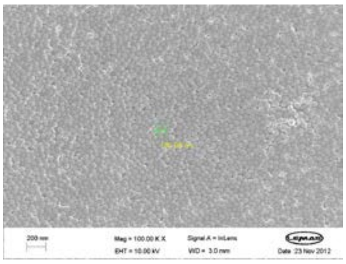

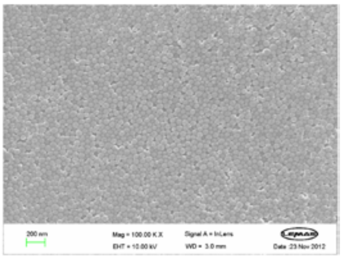

SEM-EDX

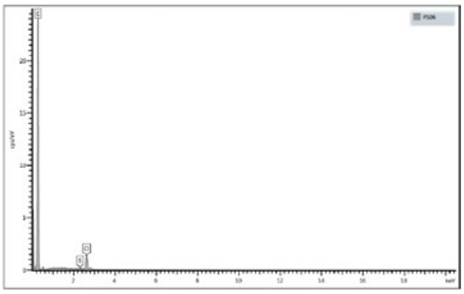

Radical formation potential by DCF-Test:

(H_2O_2 as positive control) (KIT)

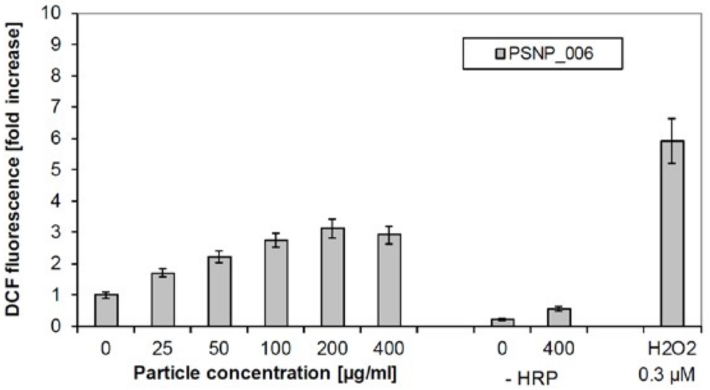

Result: PSNP_006 induced a moderate increase of DCFH oxidation (3.1 fold of nanoparticle free control at 200 µg/ml). At high concentrations, the DCF fluorescence decreased probably due to adsorption of the dye on the particle surface.

Comments and other relevant physical-chemical properties and material characterization information

**Place, Date Karlsruhe, 09.04.2013**	**Responsible H.-R. Paur, KIT**

Supplemental S7: Physicochemical characterization data sheets of ceria NMs

**REPRESENTATIVE TEST PARTICLES**<br>Nanomaterial Identification<br>according to OECD<br>ENV/JM/MONO(2009)20/REV pp29.	Research Infrastructure QualityNano
Nanomaterial name: CeO_2<br>Particle Code: CeO_2NP_001<br><br>Composition: CeO_2<br>Method of production: Sol Gel synthesis	**Manufacturer /Institute/Date**<br>**Jordi Piella Bagaria; provided by ICN; 12/2012**<br>**Technology Expert:** Jordi Piella Bagaria
**Kind of suspension:**	**Suspension** / **Powder**<br>Suspended in: water<br>pH: ______________<br>stabilizer: none

**Property**		**unit**	**Method / Institute**	**Value**
Agglomeration/aggregation			HRTEM (KIT)	Agglomerated and crystalline
Crystalline phase			D5000 Diffractometer (UU)<br>PANalytical<br>X´Pert diffractometer (ICN)	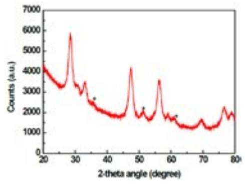
Crystallite size			HRTEM (KIT)	
Octanol-water partition coefficient			Determination of absorbance (ICN)	P_{OW}=0
Photocatalytic activity			Rhodamin-B Bleaching/UV-B-100<br>0.05mg/ml in Water (ICN)	none
Porosity		-/- or %	n.a.	n.a.
Pour density		cm3/g	(BJH) Pore Size Distribution and Volume by ASAP2020 (UU)	n.a.
Size distribution: (suspension in water)	Modal value X_M (PDI)	nm	Zetasizer Nano ZS /UCD<br><br>UNIVLEEDS<br><br>DCS (UCD) (RWtAv / RNumAv)	74.03 (PDI:0.407)<br>NM: 26.31<br><br>105.5 (PDI:0.26)
	Total concentration	mg/ml		3.2
Size distribution: (Aerosol)	Modal value X_M (σ_{geo})	nm	SMPS TSI, DMA 3071 with CPC 3022A / KIT<br>dispersed by electrospray	46 (σ_{geo}=1.13)
	Total concentration	mg/cm³ or #/cm³		8.18 x 10^3/cm^3
Solubility in _____		g/l		
Solubility in H_2O		g/l	Extraction/ ICP-MS (ICN)	
Specific surface area		m²/g	BET by ASAP2020 (UU)	n.a.
Surface chemistry				
Zeta potential (surface charge)		mV	Zetasizer Nano ZS /UU	+49.3 ± 1.3<br>pH: 3.81

Summary of trace analyses		ICP-MS / KIT	CeO	CeO_2
Al	µg/g		27	22
B	µg/g		4	3
Ba	µg/g		8.2	6.7
Ca	µg/g		83	68
Gd	µg/g		19	16
La	µg/g		1.3	1.1
Lu	µg/g		0.4	0.3
Mg	µg/g		1.4	1.2
Mn	µg/g		0.3	0.2
Nd	µg/g		0.8	0.6
Pb	µg/g		0.2	0.1
Pt	µg/g		0.6	0.5
Sm	µg/g		2.0	1.6
Sr	µg/g		0.5	0.4
Ti	µg/g		41	33
Yb	µg/g		2.7	2.2
Zn	µg/g		3	2
Zr	µg/g		0.8	0.7

Electron microscopy

Method / technical data of microscope:

SOP: See KIT (LEM)

Magnification: 450.000

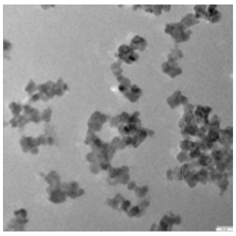

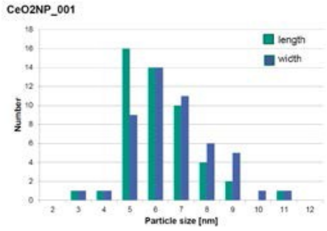

Electron Microscopy by KIT (LEM); Diameter: maximum around 10 nm

UNIVLEEDS

TEM

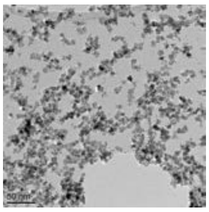

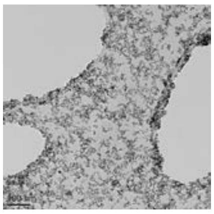

Radical formation potential by DCF-Test:

(H_2O_2 as positive control) (KIT)

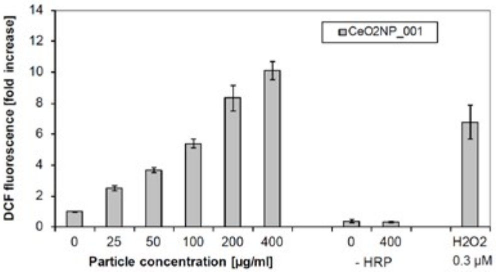

Result: CeO2 NP_001 induced a very high increase of DCFH oxidation at high concentration of 400 µg/ml (10.1 fold of nanoparticle free control).

Comments and other relevant physical-chemical properties and material characterization information

Place, Date Karlsruhe, 08.04.2013	Responsible H.-R. Paur, KIT

REPRESENTATIVE TEST PARTICLES
Nanomaterial Identification
according to OECD
ENV/JM/MONO(2009)20/REV pp29.

Research Infrastructure QualityNano

Nanomaterial name: CeO_2
Particle Code: CeO_2NP_002

Composition: CeO_2
Method of production: Sol Gel synthesis

Manufacturer /Institute/Date

Jordi Piella Bagaria; provided by ICN; 12/2012

Technology Expert: Jordi Piella Bagaria

Kind of suspension:

	**Suspension**	**Powder**
Suspended in		water
pH		______________
stabilizer		none

**Property**		**unit**	**Method / Institute**	**Value**
Agglomeration/aggregation			HRTEM (KIT)	Agglomerated and crystalline
Crystalline phase			D5000 Diffractometer (UU)<br>PANalytical X´Pert diffractometer (ICN)	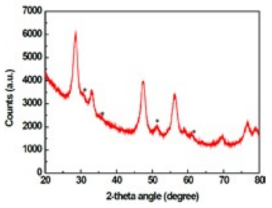
Crystallite size			HRTEM (KIT)	
Octanol-water partition coefficient			Determination of absorbance (ICN)	P_{OW}=0
Photocatalytic activity			Rhodamin-B Bleaching/UV-B-100 0.05mg/ml in Water (ICN)	none
Porosity		-/- or %	n.a.	n.a.
Pour density		cm3/g	(BJH) Pore Size Distribution and Volume by ASAP2020 (UU)	n.a.
Size distribution: (suspension in water)	Modal value X_M (PDI)	nm	Zetasizer Nano ZS /UCD<br>UNIVLEEDS<br>DCS (UCD) (RWtAv / RNumAv)	138 (PDI:0.367) NM: 24.65<br>240.3 (PDI:0.527)
	Total concentration	mg/ml		3.2
Size distribution: (Aerosol)	Modal value X_M (σ_{geo})	nm	SMPS TSI. DMA 3071 with CPC 3022A / KIT<br>dispersed by electrospray	59 (σ_{geo}=1.18)
	Total concentration	mg/cm³ or #/cm³		1.8×10^4/cm^3
Solubility in _____		g/l		
Solubility in H_2O		g/l	Extraction/ ICP-MS (ICN)	
Specific surface area		m²/g	BET by ASAP2020 (UU)	n.a.
Surface chemistry				
Zeta potential (surface charge)		mV	Zetasizer Nano ZS /UU	+48.5 ± 1.6<br>pH: 3.69

Summary of trace analyses		ICP-MS / KIT	CeO	CeO_2
Al	µg/g		27	22
B	µg/g		4	3
Ba	µg/g		8.2	6.7
Ca	µg/g		83	68
Gd	µg/g		19	16
La	µg/g		1.3	1.1
Lu	µg/g		0.4	0.3
Mg	µg/g		1.4	1.2
Mn	µg/g		0.3	0.2
Nd	µg/g		0.8	0.6
Pb	µg/g		0.2	0.1
Pt	µg/g		0.6	0.5
Sm	µg/g		2.0	1.6
Sr	µg/g		0.5	0.4
Ti	µg/g		41	33
Yb	µg/g		2.7	2.2
Zn	µg/g		3	2
Zr	µg/g		0.8	0.7

Electron microscopy

Method / technical data of microscope:

SOP: See KIT (LEM)

Magnification: 450.000

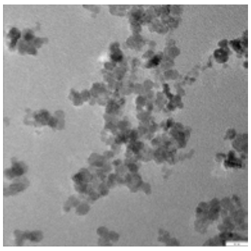

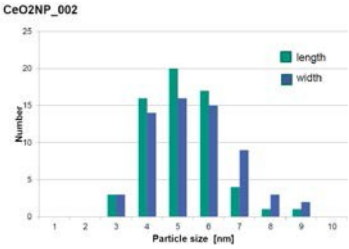

Electron Microscopy by KIT (LEM); Diameter: smaller than 10 nm

UNIVLEEDS

TEM

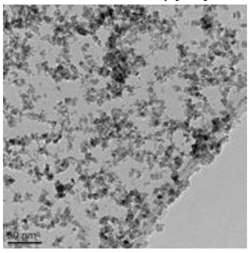

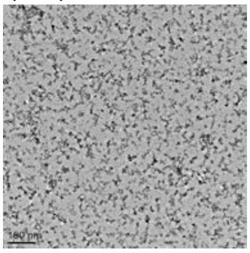

Radical formation potential by DCF-Test:

(H_2O_2 as positive control) (KIT)

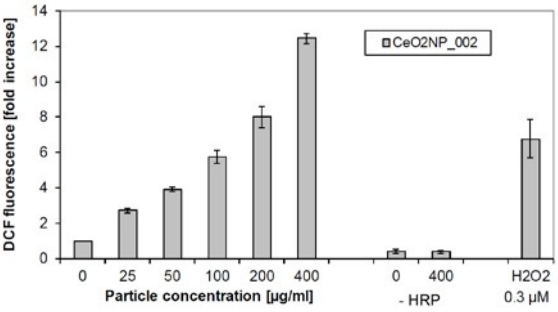

Result: CeO2 NP_002 induced a very high increase of DCFH oxidation at high concentration of 400 µg/ml (12.4 fold of nanoparticle free control).

Comments and other relevant physical-chemical properties and material characterization information

Place, Date Karlsruhe, 08.04.2013	Responsible H.-R. Paur, KIT

**REPRESENTATIVE TEST PARTICLES**<br>Nanomaterial Identification<br>according to OECD<br>ENV/JM/MONO(2009)20/REV pp29.	QualityNano Research Infrastructure
Nanomaterial name CeO_2<br>Particle Code CeO_2NP_003	**Manufacturer /Institute/Date**<br>**Jordi Piella Bagaria; provided by ICN; 12/2012**<br>**Technology Expert:** Jordi Piella Bagaria
Composition CeO_2<br>Method of production Sol Gel synthesis	
**Kind of suspension:**	**Suspension** **Powder**<br>Suspended in: water<br>pH: ____________<br>stabilizer: none

**Property**		**unit**	**Method / Institute**	**Value**
Agglomeration/aggregation			HRTEM (KIT)	Agglomerated and crystalline
Crystalline phase			D5000 Diffractometer (UU)<br>PANalytical X´Pert diffractometer (ICN)	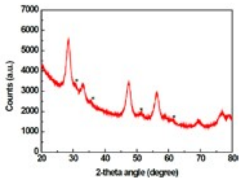
Crystallite size			HRTEM (KIT)	
Octanol-water partition coefficient			Determination of absorbance (ICN)	P_{OW}=0
Photocatalytic activity			Rhodamin-B Bleaching/UV-B-100<br>0.05mg/ml in Water (ICN)	none
Porosity		-/- or %	n.a.	n.a.
Pour density		cm3/g	(BJH) Pore Size Distribution and Volume by ASAP2020 (UU)	n.a.
Size distribution:<br>(suspension in water)	Modal value X_M (PDI)	nm	Zetasizer Nano ZS /UCD<br>UNIVLEEDS<br>DCS (UCD) (RWtAv / RNumAv)	146.1 (PDI:0.423)<br>NM: 25.47<br>147.5 (PDI:0.347)
	Total concentration	mg/ml		3.2
Size distribution:<br>(Aerosol)	Modal value X_M (σ_{geo})	nm	SMPS TSI. DMA 3071 with CPC 3022A / KIT<br>dispersed by electrospray	40.5 (σ_{geo}=1.15)
	Total concentration	mg/cm³ or #/cm³		3.5 x 10^4/cm^3
Solubility in _____		g/l		
Solubility in H_2O		g/l	Extraction/ ICP-MS (ICN)	
Specific surface area		m²/g	BET by ASAP2020 (UU)	n.a.
Surface chemistry				
Zeta potential (surface charge)		mV	Zetasizer Nano ZS /UU	+46.4 ± 0.7<br>pH: 3.66

Summary of trace analyses		ICP-MS / KIT	CeO	CeO_2
Al	µg/g		34	27
B	µg/g		7	5
Ba	µg/g		4.3	3.5
Ca	µg/g		139	113
Gd	µg/g		23	19
La	µg/g		1.7	1.4
Lu	µg/g		0.4	0.4
Mg	µg/g		2.9	2.4
Mn	µg/g		1.3	1.0
Nd	µg/g		0.9	0.7
Ni	µg/g		3.4	2.7
Pb	µg/g		0.3	0.2
Pt	µg/g		0.6	0.5
Sm	µg/g		2.2	1.8
Sr	µg/g		0.6	0.5
Ti	µg/g		1.7	1.4
Yb	µg/g		3.3	2.6
Zn	µg/g		5	4
Zr	µg/g		1.0	0.8

Electron microscopy
Method / technical data of microscope:
SOP: See KIT (LEM)

Magnification: 450.000

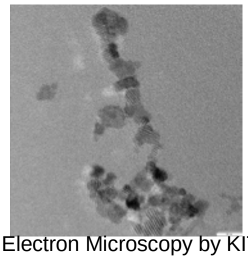

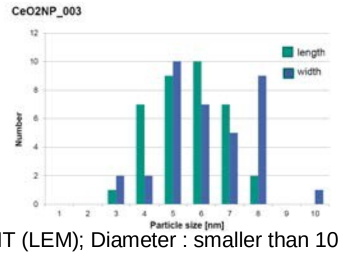

Electron Microscopy by KIT (LEM); Diameter : smaller than 10 nm

UNIVLEEDS
TEM

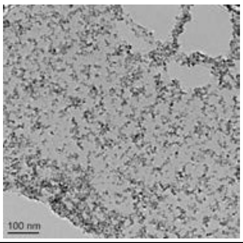

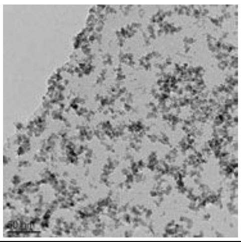

Radical formation potential by DCF-Test:

(H_2O_2 as positive control) (KIT)

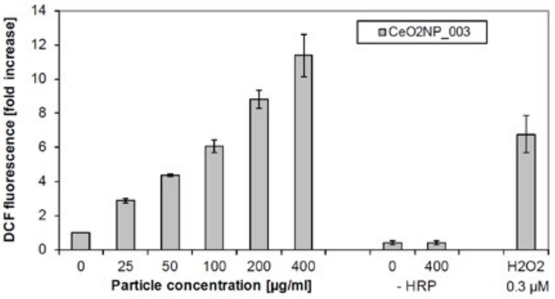

Result: CeO2 NP_003 induced a very high increase of DCFH oxidation at high concentration of 400 µg/ml (11.4 fold of nanoparticle free control).

Comments and other relevant physical-chemical properties and material characterization information

**Place, Date Karlsruhe, 08.04.2013**	**Responsible H.-R. Paur, KIT**

REPRESENTATIVE TEST PARTICLES

Nanomaterial Identification
according to OECD
ENV/JM/MONO(2009)20/REV pp29.

Research Infrastructure QualityNano

Nanomaterial name	CeO_2	**Manufacturer /Institute/Date**
Particle Code	CeO_2NP_004	**Jordi Piella Bagaria; provided by ICN; 12/2012**
		**Technology Expert:** Jordi Piella Bagaria
Composition	CeO_2	
Method of production	Sol Gel synthesis	

Kind of suspension:

**Suspension**	**Powder**
Suspended in	water
pH	______________
stabilizer	none

**Property**		**unit**	**Method / Institute**	**Value**
Agglomeration/aggregation			HRTEM (KIT)	Agglomerated and crystalline
Crystalline phase			D5000 Diffractometer (UU)<br>PANalytical<br>X´Pert diffractometer (ICN)	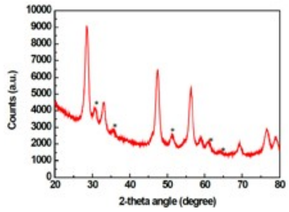
Crystallite size			HRTEM (KIT)	
Octanol-water partition coefficient			Determination of absorbance (ICN)	P_{OW}=0
Photocatalytic activity			Rhodamin-B Bleaching/UV-B-100<br>0,05mg/ml in Water (ICN)	none
Porosity		-/- or %	n.a.	n.a.
Pour density		cm^3/g	(BJH) Pore Size Distribution and Volume by ASAP2020 (UU)	n.a.
Size distribution:<br>(suspension in water)	Modal value X_M (PDI)	nm	Zetasizer Nano ZS /UCD<br><br>UNIVLEEDS<br><br>DCS (UCD) (RWtAv / RNumAv)	63.21 (PDI:0.199)<br>NM: 31.86<br><br>52.7 (PDI:0.172)
	Total concentration	mg/ml		3.2
Size distribution:<br>(Aerosol)	Modal value X_M (σ_{geo})	nm	SMPS TSI. DMA 3071 with CPC 3022A / KIT<br>dispersed by electrospray	74.5 (σ_{geo}=1.25)
	Total concentration	mg/cm^3 or $\#/cm^3$		$3.1 \times 10^4/cm^3$
Solubility in _____		g/l		
Solubility in H_2O		g/l	Extraction/ ICP-MS (ICN)	
Specific surface area		m^2/g	BET by ASAP2020 (UU)	n.a.
Surface chemistry				
Zeta potential (surface charge)		mV	Zetasizer Nano ZS /UU	+39.2 ± 0.5<br>pH: 5.97

Summary of trace analyses		ICP-MS / KIT	CeO	CeO_2
Al	µg/g		13	11
B	µg/g		3	3
Ba	µg/g		1.6	1.3
Ca	µg/g		43	35
Gd	µg/g		24	20
La	µg/g		1.7	1.4
Lu	µg/g		0.4	0.3
Mg	µg/g		2.8	2.3
Nd	µg/g		0.7	0.5
Pb	µg/g		0.1	0.06
Pt	µg/g		0.1	0.05
Sm	µg/g		1.7	1.4
Sr	µg/g		0.5	0.4
Yb	µg/g		3.2	2.6
Zn	µg/g		23	19
Zr	µg/g		1.1	0.9

Electron microscopy

Method / technical data of microscope:

SOP: See KIT (LEM)

Magnification: 450.000

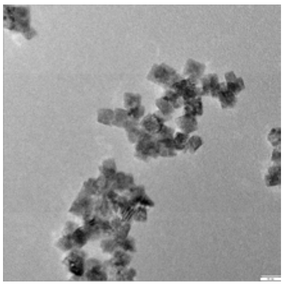

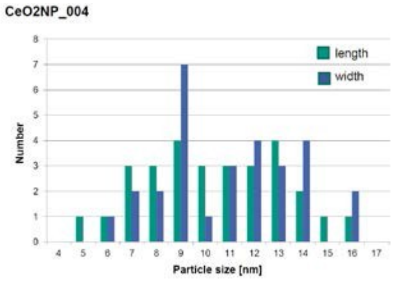

Electron Microscopy by KIT (LEM); Diameter : maximum around 10 nm

UNIVLEEDS

TEM

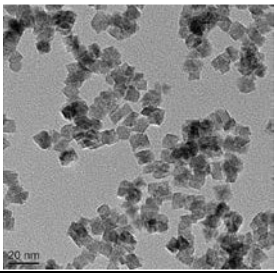

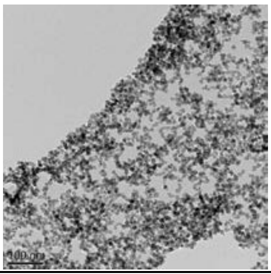

Radical formation potential by DCF-Test:

(H_2O_2 as positive control) (KIT)

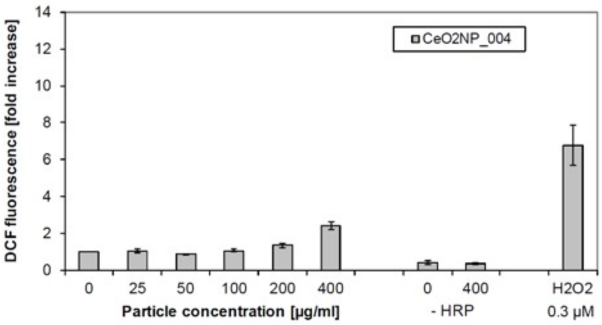

Result: CeO2 NP_004 induced a very low increase of DCFH oxidation at high concentration of 400 µg/ml (2.4 fold of nanoparticle free control).

Comments and other relevant physical-chemical properties and material characterization information

**Place, Date Karlsruhe, 08.04.2013**	**Responsible H.-R. Paur, KIT**

REPRESENTATIVE TEST PARTICLES
Nanomaterial Identification
according to OECD
ENV/JM/MONO(2009)20/REV pp29.

Research Infrastructure QualityNano

Nanomaterial name	CeO_2
Particle Code	CeO_2NP_005
Composition	CeO_2
Method of production	Sol Gel synthesis

Manufacturer /Institute/Date

Jordi Piella Bagaria; provided by ICN; 12/2012

Technology Expert: Jordi Piella Bagaria

Kind of suspension:

	**Suspension**	**Powder**
Suspended in	water	
pH	____________	
stabilizer	none	

**Property**		**unit**	**Method / Institute**	**Value**
Agglomeration/aggregation			HRTEM (KIT)	Agglomerated and crystalline
Crystalline phase			D5000 Diffractometer (UU)<br>PANalytical<br>X´Pert diffractometer (ICN)	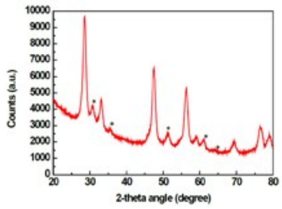
Crystallite size			HRTEM (KIT)	
Octanol-water partition coefficient			Determination of absorbance (ICN)	P_{OW}=0
Photocatalytic activity			Rhodamin-B Bleaching/UV-B-100<br>0,05mg/ml in Water (ICN)	none
Porosity		-/- or %	n.a.	n.a.
Pour density		cm3/g	(BJH) Pore Size Distribution and Volume by ASAP2020 (UU)	n.a.
Size distribution: (suspension in water)	Modal value X_M (PDI)	nm	Zetasizer Nano ZS /UCD<br><br>UNIVLEEDS<br><br>DCS (UCD) (RWtAv / RNumAv)	71.3 (PDI:0.212)<br>NM: 36.4<br><br>56.08 (PDI:0.179)
	Total concentration	mg/ml		3.2
Size distribution: (Aerosol)	Modal value X_M (σ_{geo})	nm	SMPS TSI. DMA 3071 with CPC 3022A / KIT<br>dispersed by electrospray	70.0<br>(σ_{geo}=1.30)
	Total concentration	mg/cm^3 or $\#/cm^3$		$1.0 \times 10^4/cm^3$
Solubility in _____		g/l		
Solubility in H_2O		g/l	Extraction/ ICP-MS (ICN)	
Specific surface area		m^2/g	BET by ASAP2020 (UU)	n.a.
Surface chemistry				
Zeta potential (surface charge)		mV	Zetasizer Nano ZS /UU	+33.5 ± 4.9<br>pH: 6.04

Summary of trace analyses		ICP-MS / KIT	CeO	CeO_2
Al	µg/g		10	8
B	µg/g		3	2
Ba	µg/g		1.5	1.2
Ca	µg/g		37	30
Gd	µg/g		25	20
La	µg/g		1.8	1.4
Lu	µg/g		0.4	0.3
Nd	µg/g		0.7	0.6
Pb	µg/g		0.2	0.2
Pt	µg/g		0.1	0.1
Sm	µg/g		1.7	1.4
Yb	µg/g		3.4	2.8
Zn	µg/g		24	19
Zr	µg/g		1.1	0.9

Electron microscopy
Method / technical data of microscope:
SOP: See KIT (LEM)

Magnification: 450.000

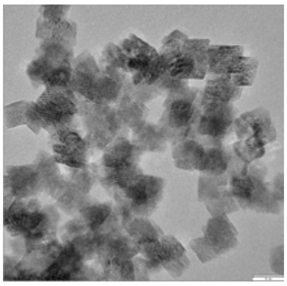

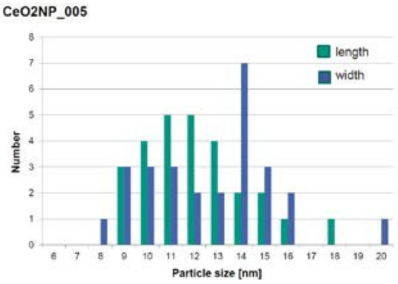

Electron Microscopy by KIT (LEM); Diameter: 8 to 20 nm

UNIVLEEDS
TEM

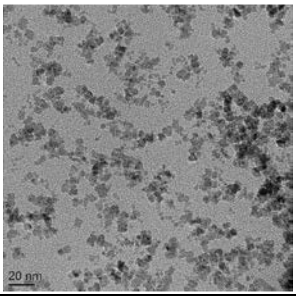

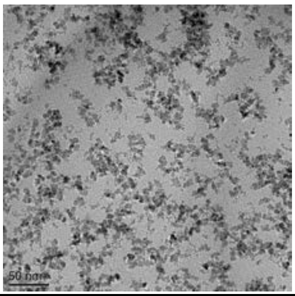

Radical formation potential by DCF-Test:
(H_2O_2 as positive control) (KIT)

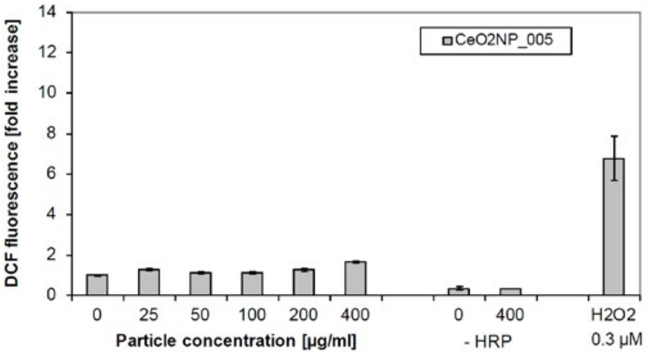

Result: CeO2 NP_005 induced a very low increase of DCFH oxidation at high concentration of 400 µg/ml (1.7 fold of nanoparticle free control).

Comments and other relevant physical-chemical properties and material characterization information

**Place, Date Karlsruhe, 08.04.2013**	**Responsible H.-R. Paur, KIT**

REPRESENTATIVE TEST PARTICLES
Nanomaterial Identification
according to OECD
ENV/JM/MONO(2009)20/REV pp29.

Research Infrastructure QualityNano

Nanomaterial name	CeO_2
Particle Code	CeO_2NP_006
Composition	CeO_2
Method of production	Sol Gel synthesis

Manufacturer /Institute/Date

Jordi Piella Bagaria; provided by ICN; 12/2012

Technology Expert: Jordi Piella Bagaria

Kind of suspension:

**Suspension**	**Powder**
Suspended in	water
pH	______________
stabilizer	none

**Property**	**unit**	**Method / Institute**	**Value**
Agglomeration/aggregation		HRTEM (KIT)	Agglomerated and crystalline
Crystalline phase		D5000 Diffractometer (UU)<br>PANalytical<br>X´Pert diffractometer (ICN)	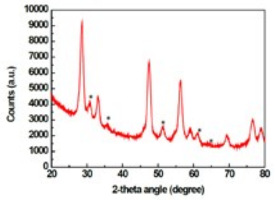
Crystallite size		HRTEM (KIT)	
Octanol-water partition coefficient		Determination of absorbance (ICN)	P_{OW}=0
Photocatalytic activity		Rhodamin-B Bleaching/UV-B-100<br>0,05mg/ml in Water (ICN)	none
Porosity	-/- or %	n.a.	n.a.
Pour density	cm3/g	(BJH) Pore Size Distribution and Volume by ASAP2020 (UU)	n.a.
Size distribution: (suspension in water) — Modal value X_M (PDI)	nm	Zetasizer Nano ZS /UCD<br>UNIVLEEDS<br>DCS (UCD) (RWtAv / RNumAv)	71.6 (PDI:0.187)<br>NM: 30.21<br>63.9 (PDI:0.618)
Size distribution: (suspension in water) — Total concentration	mg/ml		3.2
Size distribution: (Aerosol) — Modal value X_M (σ_{geo})	nm	SMPS TSI. DMA 3071 with CPC 3022A / KIT<br>dispersed by electrospray	64.0 (σ_{geo}=1.40)
Size distribution: (Aerosol) — Total concentration	mg/cm³ or #/cm³		2.2 x 10^4/cm^3
Solubility in _____	g/l		
Solubility in H_2O	g/l	Extraction/ ICP-MS (ICN)	
Specific surface area	m²/g	BET by ASAP2020 (UU)	n.a.
Surface chemistry			
Zeta potential (surface charge)	mV	Zetasizer Nano ZS /UU	+30.5 ± 2.2<br>pH: 6.04

Summary of trace analyses		ICP-MS / KIT	CeO	CeO_2
Al	µg/g		7	6
B	µg/g		2	2
Ba	µg/g		2.5	2.0
Ca	µg/g		70	57
Gd	µg/g		23	19
La	µg/g		1.6	1.3
Lu	µg/g		0.4	0.3
Nd	µg/g		0.6	0.5
Pb	µg/g		0.1	0.1
Pt	µg/g		1.0	0.8
Sm	µg/g		1.4	1.2
Ti	µg/g		3.6	3.0
Yb	µg/g		3.2	2.6
Zn	µg/g		22	18
Zr	µg/g		0.9	0.8

Electron microscopy
Method / technical data of microscope:
SOP: See KIT (LEM)

Magnification: 450.000

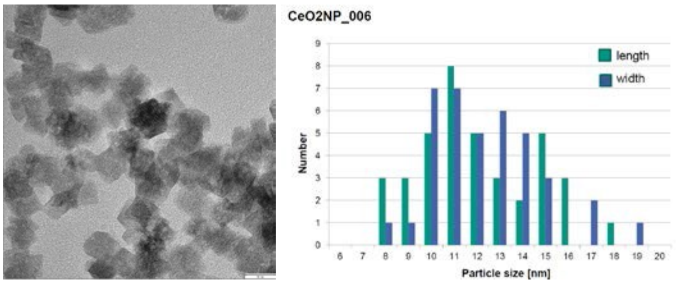

Electron Microscopy by KIT (LEM); Diameter: 8 to 20 nm

UNIVLEEDS
TEM

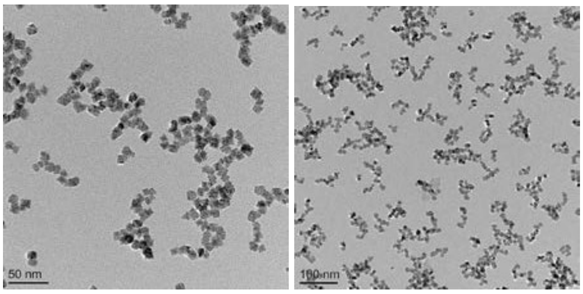

Radical formation potential by DCF-Test:

(H_2O_2 as positive control) (KIT)

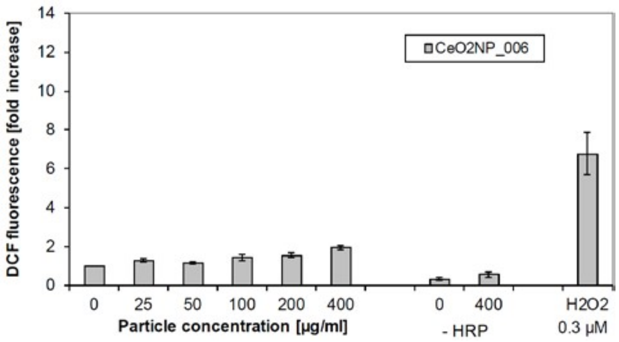

Result: CeO2 NP_006 induced a very low increase of DCFH oxidation at high concentration of 400 µg/ml (2.0 fold of nanoparticle free control).

Comments and other relevant physical-chemical properties and material characterization information

**Place, Date Karlsruhe, 08.04.2013**	**Responsible H.-R. Paur, KIT**

REPRESENTATIVE TEST PARTICLES
Nanomaterial Identification
according to OECD
ENV/JM/MONO(2009)20/REV pp29.

Research Infrastructure QualityNano

Nanomaterial name	CeO_2
Particle Code	CeO_2NP_007
Composition	CeO_2
Method of production	Sol Gel synthesis

Manufacturer /Institute/Date

Jordi Piella Bagaria; provided by ICN; 12/2012

Technology Expert: Jordi Piella Bagaria

Kind of suspension:

**Suspension**	**Powder**
Suspended in	water
pH	____________
stabilizer	none

**Property**		**unit**	**Method / Institute**	**Value**
Agglomeration/aggregation			HRTEM (KIT)	Agglomerated and crystalline
Crystalline phase			D5000 Diffractometer (UU)<br>PANalytical<br>X´Pert diffractometer (ICN)	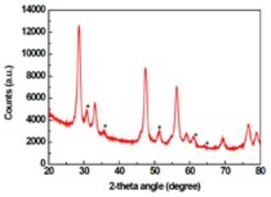
Crystallite size			HRTEM (KIT)	
Octanol-water partition coefficient			Determination of absorbance (ICN)	P_{OW}=0
Photocatalytic activity			Rhodamin-B Bleaching/UV-B-100<br>0,05mg/ml in Water (ICN)	high
Porosity		-/- or %	n.a.	n.a.
Pour density		cm^3/g	(BJH) Pore Size Distribution and Volume by ASAP2020 (UU)	n.a.
Size distribution:<br>(suspension in water)	Modal value X_M (PDI)	nm	Zetasizer Nano ZS /UCD<br>UNIVLEEDS<br>DCS (UCD) (RWtAv / RNumAv)	74.29 (PDI:0.204)<br>NM: 40.26<br>64.79 (PDI:0.166)
	Total concentration	mg/ml		3.2
Size distribution:<br>(Aerosol)	Modal value X_M (σ_{geo})	nm	SMPS TSI. DMA 3071 with CPC 3022A / KIT<br>dispersed by electrospray	71.1 (σ_{geo}=1.18)
	Total concentration	mg/cm^3 or $\#/cm^3$		2.2 x $10^4/cm^3$
Solubility in _____		g/l		
Solubility in H_2O		g/l	Extraction/ ICP-MS (ICN)	
Specific surface area		m^2/g	BET by ASAP2020 (UU)	n.a.
Surface chemistry				
Zeta potential (surface charge)		mV	Zetasizer Nano ZS /UU	+39.3 ± 0.8<br>pH: 6.1

Summary of trace analyses		ICP-MS / KIT	CeO	CeO_2
Al	µg/g		8	7
B	µg/g		5	4
Ba	µg/g		2.3	1.9
Ca	µg/g		53	43
Gd	µg/g		22	18
La	µg/g		1.6	1.3
Lu	µg/g		0.4	0.3
Nd	µg/g		0.6	0.5
Pt	µg/g		0.2	0.1
Sm	µg/g		1.3	1.1
Ti	µg/g		6.0	4.9
Yb	µg/g		2.8	2.3
Zn	µg/g		19	15
Zr	µg/g		0.9	0.8

Electron microscopy
Method / technical data of microscope:
SOP: See KIT (LEM)

Magnification: 450.000

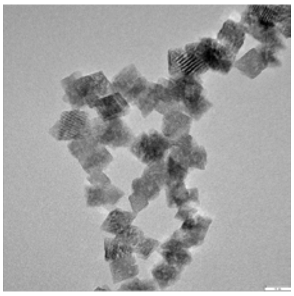

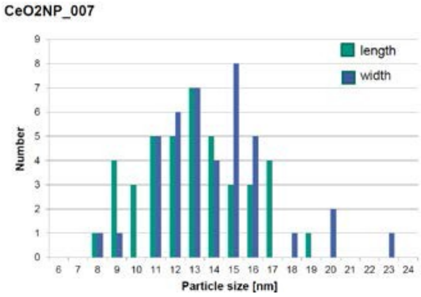

Electron Microscopy by KIT (LEM); Diameter : Rough estimate of particle-size distribution, the maximum of the distribution seems to be between 13 to 15 nm

UNIVLEEDS
TEM

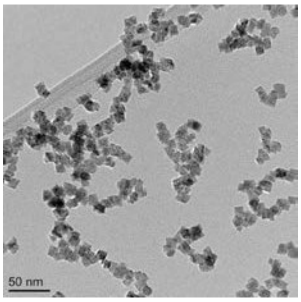

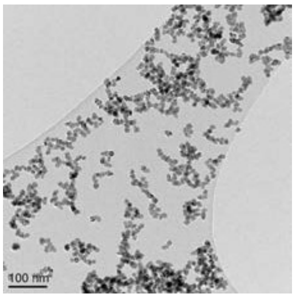

Radical formation potential by DCF-Test:

(H_2O_2 as positive control) (KIT)

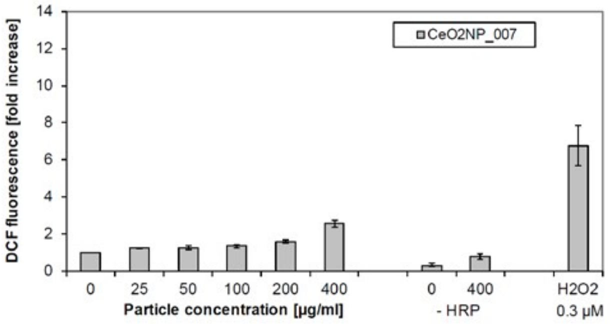

Result: CeO2 NP_007 induced a low increase of DCFH oxidation at high concentration of 400 µg/ml (2.6 fold of nanoparticle free control).

Comments and other relevant physical-chemical properties and material characterization information

**Place, Date Karlsruhe, 08.04.2013**	**Responsible H.-R. Paur, KIT**

**REPRESENTATIVE TEST PARTICLES**<br>Nanomaterial Identification<br>according to OECD<br>ENV/JM/MONO(2009)20/REV pp29.	QualityNano Research Infrastructure
Nanomaterial name: CeO_2<br>Particle Code: CeO_2NP_008	**Manufacturer /Institute/Date**<br>**Jordi Piella Bagaria; provided by ICN; 12/2012**<br>**Technology Expert:** Jordi Piella Bagaria
Composition: CeO_2<br>Method of production: Sol Gel synthesis	
**Kind of suspension:**	**Suspension** / **Powder**<br>Suspended in: water<br>pH: ______________<br>stabilizer: none

**Property**		**unit**	**Method / Institute**	**Value**
Agglomeration/aggregation			HRTEM (KIT)	Agglomerated and crystalline
Crystalline phase			D5000 Diffractometer (UU)<br>PANalytical<br>X´Pert diffractometer (ICN)	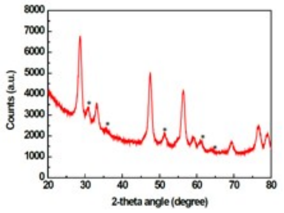
Crystallite size			HRTEM (KIT)	
Octanol-water partition coefficient			Determination of absorbance (ICN)	P_{OW}=0
Photocatalytic activity			Rhodamin-B Bleaching/UV-B-100<br>0.05mg/ml in Water (ICN)	none
Porosity		-/- or %	n.a.	n.a.
Pour density		cm3/g	(BJH) Pore Size Distribution and Volume by ASAP2020 (UU)	n.a.
Size distribution:<br>(suspension in water)	Modal value X_M (PDI)	nm	Zetasizer Nano ZS /UCD<br><br>UNIVLEEDS<br><br>DCS (UCD) (RWtAv / RNumAv)	65.34 (PDI:0.158)<br>NM: 36.9<br><br>63.61 (PDI:0.153)
	Total concentration	mg/ml		3.2
Size distribution:<br>(Aerosol)	Modal value X_M (σ_{geo})	nm	SMPS TSI. DMA 3071 with CPC 3022A / KIT<br>dispersed by electrospray	65.0 (σ_{geo}=1.22)
	Total concentration	mg/cm³ or #/cm³		8.4 x 10^4/cm^3
Solubility in _____		g/l		
Solubility in H_2O		g/l	Extraction/ ICP-MS (ICN)	
Specific surface area		m²/g	BET by ASAP2020 (UU)	n.a.
Surface chemistry				
Zeta potential (surface charge)		mV	Zetasizer Nano ZS /UU	+33.5 ± 3.6<br>pH: 6.04

Summary of trace analyses		ICP-MS / KIT	CeO	CeO_2
Al	µg/g		8	6
B	µg/g		2	2
Ba	µg/g		1.8	1.5
Ca	µg/g		59	48
Gd	µg/g		24	19
La	µg/g		1.8	1.5
Lu	µg/g		0.4	0.3
Nd	µg/g		0.6	0.5
Pt	µg/g		0.1	0.05
Sm	µg/g		1.4	1.1
Sn	µg/g		0.3	0.2
Ti	µg/g		2.8	2.3
Yb	µg/g		3.0	2.4
Zn	µg/g		20	16
Zr	µg/g		1.0	0.8

Electron microscopy
Method / technical data of microscope:
SOP: See KIT (LEM)

magnification: 450.000

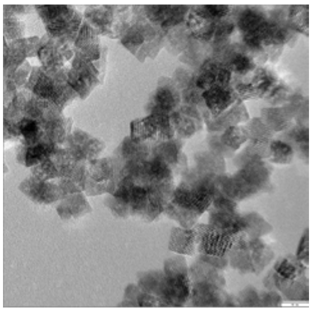

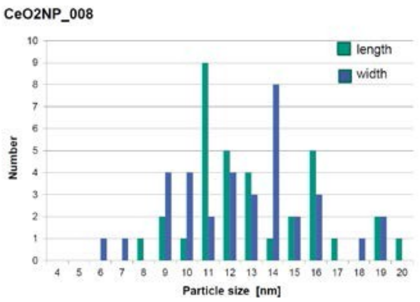

Electron Microscopy by KIT (LEM); Diameter: 5 to 20 nm

UNIVLEEDS
TEM

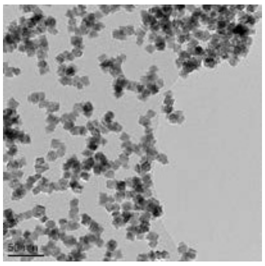

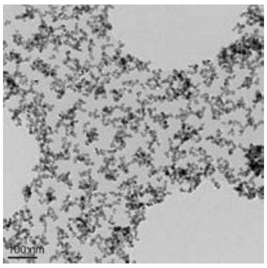

Radical formation potential by DCF-Test:
(H_2O_2 as positive control) (KIT)

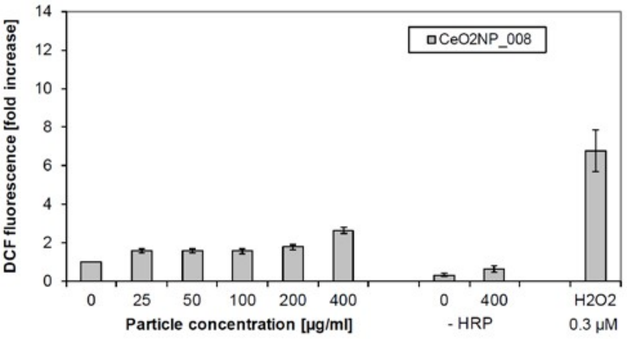

Result: CeO2 NP_008 induced a low increase of DCFH oxidation at high concentration of 400 µg/ml (2.6 fold of nanoparticle free control).

Comments and other relevant physical-chemical properties and material characterization information

**Place, Date Karlsruhe, 08.04.2013**	**Responsible H.-R. Paur. KIT**

**REPRESENTATIVE TEST PARTICLES**<br>Nanomaterial Identification<br>according to OECD<br>ENV/JM/MONO(2009)20/REV pp29.	Research Infrastructure QualityNano
Nanomaterial name CeO_2<br>Particle Code CeO_2NP_009	**Manufacturer /Institute/Date**<br>**Jordi Piella Bagaria; provided by ICN; 12/2012**<br>**Technology Expert:** Jordi Piella Bagaria
Composition CeO_2<br>Method of production Sol Gel synthesis	
**Kind of suspension:**	**Suspension** / **Powder**<br>Suspended in: <u>water</u><br>pH: ____________<br>stabilizer: none

**Property**		**unit**	**Method / Institute**	**Value**
Agglomeration/aggregation			HRTEM (KIT)	Agglomerated and crystalline
Crystalline phase			D5000 Diffractometer (UU)<br>PANalytical<br>X´Pert diffractometer (ICN)	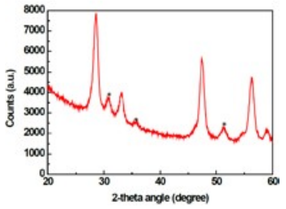
Crystallite size			HRTEM (KIT)	
Octanol-water partition coefficient			Determination of absorbance (ICN)	P_{OW}=0
Photocatalytic activity			Rhodamin-B Bleaching/UV-B-100<br>0.05mg/ml in Water (ICN)	high
Porosity		-/- or %	n.a.	n.a.
Pour density		cm3/g	(BJH) Pore Size Distribution and Volume by ASAP2020 (UU)	n.a.
Size distribution:<br>(suspension in water)	Modal value X_M (PDI)	nm	Zetasizer Nano ZS /UCD<br><br>UNIVLEEDS<br><br>DCS (UCD) (RWtAv / RNumAv)	71.05 (PDI:0.202)<br>NM: 42.14<br><br>56.32 (PDI:0.167)
	Total concentration	mg/ml		3.2
Size distribution:<br>(Aerosol)	Modal value X_M (σ_{geo})	nm	SMPS TSI. DMA 3071 with CPC 3022A / KIT<br>dispersed by electrospray	71.8 (σ_{geo}=1.45)
	Total concentration	mg/cm³ or #/cm³		9.6 x 10^4/cm^3
Solubility in _____		g/l		
Solubility in H_2O		g/l	Extraction/ ICP-MS (ICN)	
Specific surface area		m²/g	BET by ASAP2020 (UU)	n.a.
Surface chemistry				
Zeta potential (surface charge)		mV	Zetasizer Nano ZS /UU	+37.7 ± 0.5<br>pH: 6.1

Summary of trace analyses		ICP-MS / KIT	CeO	CeO_2
Al	µg/g		11	9
B	µg/g		3	2
Ba	µg/g		2.2	1.8
Ca	µg/g		40	33
Gd	µg/g		24	20
La	µg/g		1.9	1.5
Lu	µg/g		0.4	0.3
Nd	µg/g		0.6	0.5
Pt	µg/g		0.5	0.4
Sm	µg/g		1.4	1.2
Ti	µg/g		2.4	2.0
Yb	µg/g		3.2	2.6
Zn	µg/g		20	16
Zr	µg/g		1.0	0.8

Electron microscopy
Method / technical data of microscope:
SOP: See KIT (LEM)

Magnification: 450.000

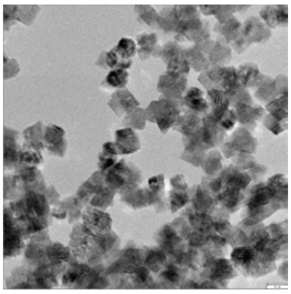

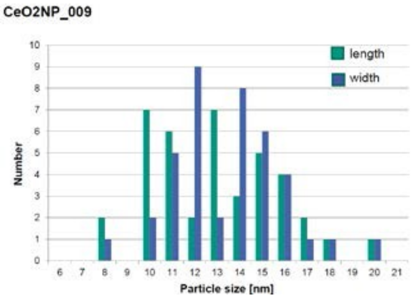

Electron Microscopy by KIT (LEM); Diameter: 8 to 20 nm

UNIVLEEDS
TEM

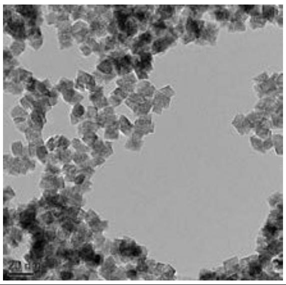

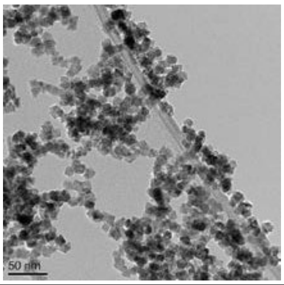

Radical formation potential by DCF-Test:

(H_2O_2 as positive control) (KIT)

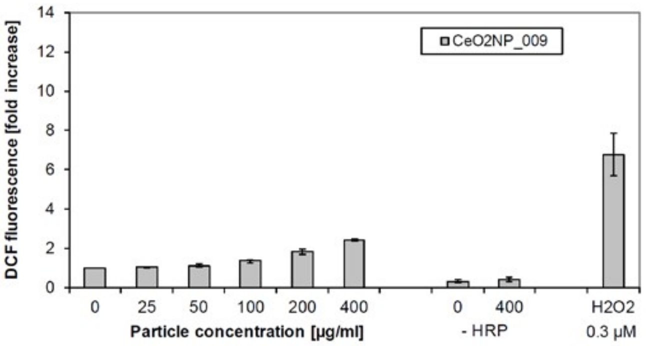

Result: CeO2 NP_009 induced a low increase of DCFH oxidation at high concentration of 400 µg/ml (2.4 fold of nanoparticle free control).

Comments and other relevant physical-chemical properties and material characterization information

**Place, Date Karlsruhe, 08.04.2013**	**Responsible H.-R. Paur, KIT**

Supplemental S8: Comparison of B2B to JRC data

Table S8-1: Comparison of physicochemical data for the B2B Stöber synthesized Silica NM and the JRC precipitated SAS (synthetic amorphous silica)

**Particle Property**	**B2B Stöber Silica NMs**	**NM-200, NM-201, NM-204**
Number of batches	8	3
Trace impurities (Main elements)	Up to 36 μg/g Na<br>Al < detection limit<br>36 to 82 μg/g Ca	> 1 μg/g Na and Al
Dissolution in water	< detection limit	Not analyzed
Crystal structure	Amorphous	Amorphous
Crystal size	--	--
Mobility diameter	33 to 118 nm	Not analyzed
Hydrodynamic diameter (DLS)	25 to 120 nm	NM200: 180 to 240 nm dep. on lab
Zeta potential (surface charge)	-45 to -66 mV (pH 8.4±0.57)	-30 to -40 mV (pH ~8)
Photocatalytic activity	No	Not analyzed
Octanol- water partition coefficient	Not analyzed	Not analyzed
Radical formation	2 – 24.2-fold	< detection limit
Reference		[2]

Table S8-2: Comparison of physicochemical data for the B2B flame synthesized silica NMs and the JRC fumed synthesized amorphous silica (SAS)

**Particle Property**	**B2B Flame Silica NMs**	**NM-202, NM-203**
Number of batches	6	2
Trace impurities (Main elements)	Up to 75 μg/g (Al)	< detection limit
Dissolution in water	6 to 10 wt %	Not analyzed
Crystal structure	Amorphous	Amorphous
Crystal size	--	--
Mobility diameter	32 to 160 nm.	Not analyzed
Hydrodynamic diameter (DLS)	200 to 380 nm	NM200: 147 to 2453nm dep. on lab
Zeta potential (surface charge)	-15 to -18 mV (pH ~4.3)	-30 to -40 mV (pH ~8)
Photocatalytic activity	No	Not analyzed
Octanol- water partition coefficient	< detection limit	Not analyzed
Radical formation	2 - 6.2-fold	< detection limit
Reference		[2]

Table S8-3: Comparison of physicochemical data for the B2B zinc oxide NM and the JRC zinc oxide NM

**Particle Property**	**B2B Zinc oxide NMs**	**NM-110, NM-111, NM-112, NM-113**
Number of batches	9	4
Trace impurities (Main elements)	7 - 77 µg/g Na<br>1.8 – 3.7 µg/g Cu<br>3 – 136 µg/g Ca<br>8 – 1080 µg/g Fe<br>19 – 60 µg/g Sr	3 - 176 µg/g Na<br>3 – 120 µg/g Cu<br>40 – 680 µg/g Ca
Dissolution in water	7 wt %	1 - 400 wt-‰
Crystal structure	Crystalline<br>hexagonal zinc oxide or ‘Wurtzite’	Crystalline
Crystal size	20 to 30 nm	24 to 42 nm
Mobility diameter	25 to 55 nm	278 nm (only NM-110)
Hydrodynamic diameter (DLS)	130 to 900 nm	253 to 508 nm
Zeta potential (surface charge)	-15 to +24 mV<br>(pH ~6)	20.2 – 24.6 mV<br>(pH not indicated)
Photocatalytic activity	High	No
Octanol- water partition coefficient	< detection limit	Not analyzed
Radical formation	3- to 4-fold	Not analyzed
Reference		[1]

Table S8-4: Comparison of physicochemical data for the B2B titania NMs and the JRC titania

**Particle Property**	**Titanium dioxide NMs**	**NM-100, NM-101, NM-102, NM-103, NM-104, NM-105**
Number of batches	8	6
Trace impurities (Main elements)	Up to 96 mg/g Na	> 1 mg/g Na and Al
Dissolution in water	n.d.	Not analyzed
Crystal structure	Crystalline<br>anatase with small fraction of brookite	Crystalline<br>anantase, rutile or mixture (NM-1005)
Crystal size	4.5 to 6 nm	< 100 nm, 57nm, > 80nm depending on lab
Mobility diameter	33 to 70 nm.	Not analyzed
Hydrodynamic diameter (DLS)	60 to 280 nm	113 to 423 nm
Zeta potential (surface charge)	33 to 40 mV<br>(pH ~10)	-40mV to >30 mV<br>depending on pH<br>pH 10: ~ -25 to -40 mV
Photocatalytic activity	Low	Not analyzed
Octanol- water partition coefficient	< detection limit	Not analyzed
Radical formation	1 - 4.8 fold	Not analyzed
Reference		[3]

Table S8-5: Comparison of physicochemical data for the B2B Cerium dioxide NMs and the JRC Cerium dioxide

**Particle Property**	**Cerium dioxide NMs**	**NM-211, NM-212, NM-213**
Number of batches	9	3
Trace impurities (Main elements)	87 to 187 µg/g Ca	100 µg/g Ca
Dissolution in water	< detection limit	1.05 to 5.085 ng/g
Crystal structure	Crystalline: cubic lattice	Crystalline: cubic lattice
Crystal size	5 to 8 nm	10.3 to 33.3 nm (NPL) or 9 to 49 nm (JRC) depending on lab
Mobility diameter	40 to 74 nm.	Not analyzed
Hydrodynamic diameter (DLS)	63 to 146 nm	213 to 349 nm
Zeta potential (surface charge)	30 to 49 mV (pH 3.8 to 6.1)	-7 to +33 mV (pH not indicated)
Photocatalytic activity	No	No or low
Octanol- water partition coefficient	< detection limit	Not analyzed
Radical formation	2 to 12-fold	Not analyzed
Reference		[4]

References

1. Singh, C.; Friedrichs, S.; Levin, M.; Birkedal, R.; Jensen, K.A.; Pojana, G.; Wohlleben, W.; Schulte, S.; Wiench, K.; Turney, T.; et al. NM-Series of Representative Manufactured Nanomaterials: Zinc Oxide NM-110, NM-111, NM-112, NM-113. Characterisation and Test Item Preparation; Publications Office of the European Union: Luxembourg, 2011.
2. Rasmussen, K.; Mech, A.; Mast, J.; Temmerman, P.-J. de; Waegeneers, N.; Steen, F. Van; Doren, E. Van; Jensen, K.A.; Birkedal, R.; Levin, M.; et al. Synthetic amorphous silicon dioxide (NM-200, NM-201, NM-202, NM-203, NM-204). Characterisation and physico-chemical properties; Publications Office of the European Union: Luxembourg, 2013.
3. Rasmussen, K.; Mast, J.; Temmerman, P.-J. de; Verleysen, E.; Waegeneers, N.; Van Steen, F.; Van Doren, E.; Jensen, K.A.; Birkedal, R.; Levin, M.; et al. Titanium dioxide, NM-100, NM-101, NM-102, NM-103, NM-104, NM-105. Characterisation and physico-chemical properties; Publications Office of the European Union: Luxembourg, 2014.
4. Singh, C.; Friedrichs, S.; Ceccone, G.; Gibson, N.; Jensen, K.A.; Levin, M.; Goenaga, H.; Carlander, D.; Rasmussen, K. Cerium Dioxide NM-211, NM-212, NM-213, characterisation and test item preparation. JRC repository: NM-series of representative manufactured nanomaterials; Publications Office of the European Union: Luxembourg, 2014.
